# Supplementary material for: The age profile of respiratory syncytial virus burden in preschool children of low- and middle-income countries: A semi-parametric, meta-regression approach
Source: PLoS Med. 2023 Jul 17;20(7):e1004250. doi: 10.1371/journal.pmed.1004250 (PMC10389726; doi:10.1371/journal.pmed.1004250)
Supplement: S1 Text — Section S1.1. Extraction of the Shi and colleagues and Li and colleagues data. Section S1.1.1. Modifications to incidence data from Shi and colleagues. Section S.1.1.2. Modifications to fatality (hCFR) data from Shi and colleagues. Section S1.2. Descriptive summaries of all data. Section S1.3. Generalized additive mixed models (GAMM). Section S1.4. Outcome Models (OM) I and II. Section S1.7. Characterizing the age profile of RSV burden (mean, median, and the peak age of each outcome). Section S1.6. Burden Models (BM) I and II. Section S1.9. Countries included, World Bank Income Group, population, and life tables. Section S1.3. Generalized additive mixed models (GAMM). Section S1.5.1. Spline specifications and computational considerations. Section S1.5.2. Predictions of the outcome model and uncertainty. Section S1.8. Severe and very severe disease. Table A. Characteristics of the studies included in the main splines and in the supplemental severity splines. Table B. Country income groups and population age <5 in 2020. Fig A. Geographic distribution of the available data. Fig B. Relationship between cases, hospitalizations, and severity from birth to 60 months of age. (PDF) [file pmed.1004250.s001.pdf]

Supporting Information

The age profile of respiratory syncytial virus burden in pre-school children of low- and middle-income countries: A semi-parametric, meta-regression approach

S1. Supplementary Methods

Contents

S1.1 Extraction of the Shi et. al and Li et. al data . . . S1.2
S1.1.1 Modifications to incidence data from Shi et. al . . . S1.2
S1.1.2 Modifications to fatality (hCFR) data from Shi et. al . . . S1.2
S1.2 Descriptive summaries of all data . . . S1.2
S1.3 Generalized additive mixed models (GAMM) . . . S1.5
S1.4 Outcome Models (OM) I & II . . . S1.6
S1.4.1 OM I: community-based studies as a basis for incidence . . . S1.6
S1.4.2 OM II: hospital-based studies as a basis for incidence . . . S1.6
S1.5 Spline Models . . . S1.6
S1.5.1 Spline specifications and computational considerations . . . S1.8
S1.5.2 Predictions of the outcome model and uncertainty . . . S1.9
S1.6 Burden Models (BM) I & II . . . S1.10
S1.6.1 BM I: burden models using OM I and II and population estimates . . . S1.10
S1.6.2 BM II: burden models combining the OM I & II, Shi et al's risk factor model of RSV incidence among all children under 5, and population estimates . . . S1.11
S1.6.3 Burden models: computational considerations and uncertainty calculations . . . S1.11
S1.7 Characterizing the age profile of RSV burden (mean, median, and the peak age of each outcome) S1.13
S1.8 Severe and very severe disease . . . S1.14
S1.8.1 OM II: hospital-based studies as a basis for incidence . . . S1.14
S1.9 Countries included, World Bank Income Group, population, and life tables . . . S1.17

List of Tables

A Characteristics of the studies included for the main splines and the supplemental severity splines. A summary of the study sizes, the observed events of interest (cases, hospitalizations, deaths), and the number of data points (mutually exclusive age groups) present in the data. . . . S1.4
B Country income groups and population age <5 in 2020. . . . S1.17
C Population in countries that were 'developing' according to UNICEF classifications but were high-income countries according to the World Bank's lending groups. These were included in Li et al's analysis but excluded in our analysis. . . . S1.17
D Countries missing from the risk-factor model by Li et al that were LIC/LMIC/UMIC countries in 2020. To make estimates comparable, these studies were also not included in our analysis. . . S1.18

List of Figures

A Geographic distribution of the available data . . . S1.3
B Relationship between cases, hospitalizations, and severity from birth to 59 months of age. . . S1.15

First, we will discuss some small details of how the data was extracted in order to function with our approach. Generalized additive mixed models will be discussed next, and then a detailed explanation of how our model choices led to the outcome models (OMs), the burden models (BMs), and our estimates of the window of vulnerability for RSV infection, hospitalization, and death.

Therefore, a brief overview of generalized additive mixed models, the family of models behind our approach will be given in section S1.3. The explicit mathematical specification behind the relationships depicted in Fig 1 will be stated in section S1.4 and the model specifications for each of the four splines will be given in section S1.5. The specification of the burden models will be discussed in detail in section S1.6. And lastly, how the mean, median, and peak age of infection were calculated will be discussed in section S1.7.

S1.1 Extraction of the Shi et. al and Li et. al data

↩ Return to the Table of Contents.

There were some small modifications to the way the data were presented by Shi et al in order to make it amenable for our approach in R [1, 2]. No modifications were necessary to the Li et al data.

S1.1.1 Modifications to incidence data from Shi et. al

Some data were only reported as estimates and standard deviations, requiring us to back-calculate for the cases and the counts. We assumed that the equation to estimate confidence intervals had been:
$\hat{\lambda} = C/P$
confidence interval of the log rate: $\log(\hat{\lambda}) \pm z_{\alpha/2}/\sqrt{C}$
confidence interval in the natural scale $[\hat{\lambda} \cdot \exp(-z_{\alpha/2}/\sqrt{C}), \hat{\lambda} \cdot \exp(z_{\alpha/2}/\sqrt{C})]$

where

P is the population time observed.
C are the cases observed.
$z_{\alpha/2}$ is the z-score for the 2.5th and 97.5th percentile, usually the value of 1.96.

The above formulation avoids negative confidence bounds – which aligns with the confidence intervals in the Shi et. al data. Therefore, we would back-calculate the cases with the following formula $(-z_{\alpha/2}/\ln(\text{low}_{\text{CI}}/\hat{\lambda}))^2$ and back-calculate the population by $C/\hat{\lambda}$.

S1.1.2 Modifications to fatality (hCFR) data from Shi et. al

When the probability of deaths in the hospital (hCFR) was presented as 0.0005, we calculated a count of 0. When no deaths were given in a category, we assumed that that category was not observed and we placed an NA instead of 0.

S1.2 Descriptive summaries of all data

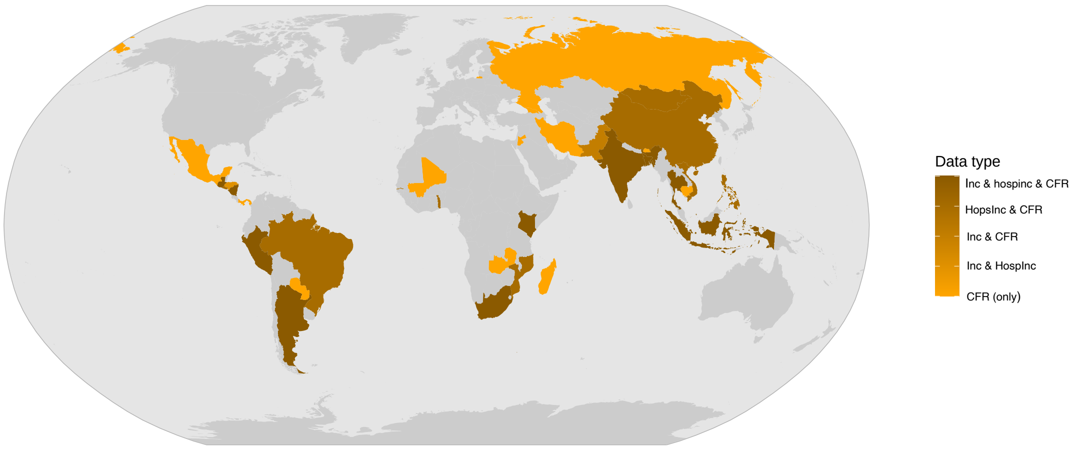

Figure A: **Geographic distribution of the available data.** Countries colored in gray were countries that we did not have data from or were high-income countries and thus excluded from our analysis.

	Study sample				Events				Data points (age groups)			
**Spline**	**Total**	**Mean**	**Median**	**Range**	**Total**	**Mean**	**Median**	**Range**	**Total**	**Mean**	**Median**	**Range**
**Community-based incidence**												
Estimation	43059	2392	933	(132, 19654)	2126	118	88	(8, 422)	100	5.6	5.0	(3, 8)
Validation	14775	7388	7388	(516, 14259)	1362	681	681	(22, 1340)	3	1.5	1.5	(1, 2)
**Hospital-based incidence**												
Estimation	4946063	133677	71160	(199, 934647)	27822	752	191	(3, 5149)	215	5.8	6.0	(3, 8)
Validation	5621386	374759	48831	(875, 3501394)	20712	1381	376	(11, 11299)	22	1.5	1.0	(1, 2)
**Probability of hospitalization (among cases in the community)**												
Estimation	609	87	95	(20, 170)	121	17	4	(3, 65)	37	5.3	5.0	(4, 7)
Validation	626	209	155	(116, 355)	33	11	16	(0, 17)	6	2.0	2.0	(1, 3)
**In-hospital deaths (hCFR)**												
Estimation	27439	473	177	(9, 3590)	347	6	1	(0, 78)	384	6.6	7.0	(3, 8)
Validation	13694	456	294	(24, 2723)	187	6	3	(0, 50)	34	1.1	1.0	(1, 2)
**Probability of severity (among cases in the community)**												
Estimation	35655	2547	1003	(132, 19654)	335	24	15	(3, 82)	77	5.5	5.0	(3, 8)
Validation	4571	914	950	(242, 1972)	72	14	11	(4, 36)	5	1.0	1.0	(1, 1)
**Probability of very severe cases (among cases in the community)**												
Estimation	24410	8137	3071	(1685, 19654)	13	4	4	(4, 5)	21	7.0	7.0	(6, 8)
Validation	4571	914	950	(242, 1972)	29	6	6	(0, 12)	5	1.0	1.0	(1, 1)
**Probability of severity (among cases in the hospital)**												
Estimation	2345004	156334	119535	(1502, 812463)	9954	664	535	(16, 2749)	91	6.1	6.0	(4, 8)
Validation	14264	14264	14264	(14264, 14264)	98	98	98	(98, 98)	2	2.0	2.0	(2, 2)
**Probability of very severe cases (among cases in the hospital)**												
Estimation	2174515	167270	119535	(1502, 812463)	11723	902	537	(30, 3185)	80	6.2	7.0	(4, 8)
Validation	14264	14264	14264	(14264, 14264)	98	98	98	(98, 98)	2	2.0	2.0	(2, 2)

Table A: Characteristics of the studies included for the main splines and the supplemental severity splines. A summary of the study sizes, the observed events of interest (cases, hospitalizations, deaths), and the number of data points (mutually exclusive age groups) present in the data.

S1.3 Generalized additive mixed models (GAMM)

↩ Return to the Table of Contents.

Generalized additive models Unlike fully parametric models in which the predictors are assumed to have a relationship to the outcome that can be characterized by linear combinations of predictors - potentially a strong or unwarranted assumption - generalized additive models do not make the assumption that the relationship between the predictor and the outcome is linear (or a simple transformation of a linear function, such as a log-linear or logit-linear function). [3, 4]

Instead, generalized additive models (GAM) are the sum of polynomials that result in a variety of flexible curves or splines. The shape of a spline is broadly determined by two components: 1) the number of "knots", or places when the curve changes direction or convexity, and 2) the "smoothing" parameter, which constrains the number of grooves and inflections of a curve to achieve both parsimony and more general predictive capacity. [5, 3, 4]

The smoothing parameter is a penalty term on the objective function, given by the second derivative of the model, thus penalizing more complicated curves. The penalty term in the smoothing splines circumvents the need to find the ideal number or location of knots in each regression model. The selection of the smoothing parameter of the penalty term is done through maximum-likelihood [5, 3, 4].

Non-normal outcomes The observed measurements are assumed to arise from probabilistic processes that can be approximated by off-the-shelf distributions (normal, binomial with logit-link, Poisson with log-link), much like the more conventional generalized linear models, and thus the statistical package employs maximum-likelihood methods (or variants of it) to estimate the placement of the knots and the parameters of the polynomials [5, 6].

Mixed-effects framework An extension to GAMs, GAMMs for mixed-effects models, makes it possible to calculate fixed- and random-effects for these models. In our analysis, the impact of age on the outcome of interest (incidence, hospitalizations, deaths) was a fixed-effect, and the random-effect accounted for the differences between studies. This approach is analogous to the "meta-regression frameworks" observed in the literature [7, 8, 9, 10, 11]. In other words, splines in a mixed-effects framework decompose the observed variance into two components: a global trend (by age) using a fixed-effect spline, and a study-specific trend using random-effect splines. Specifically, the random effect component accounts for the difference between the age-related trend observed in each study and the global trend.

S1.4 Outcome Models (OM) I & II

↩ Return to the Table of Contents.

Four splines were constructed to characterize different features of RSV epidemiology in children under the age of 5 years:

I) incidence as measured in community-based studies
II) incidence as measured in hospital-based studies
III) probability of hospitalization (measured in community-based studies)
IV) probability of death among hospitalized patients (measured in hospital-based studies)

As illustrated in Fig 1, the incidence of all cases, hospitalized cases, and deaths (in the hospital and in the community) can be estimated in two ways: 1) from the extant data by taking the incidence from community-based studies and using the probabilities to calculate hospitalizations and hospital-based deaths (OM I); or by taking the incidence from hospital-based studies (as an index of the relative level of disease at each age group) and back-calculating the incidence of disease in the community and calculating the incidence of deaths in the hospital (OM II). It should be noted that the product of incidence and probability yields an incidence.

S1.4.1 OM I: community-based studies as a basis for incidence

For OM I, the basis of incidence calculations come from community-based incidence studies:

$$\begin{aligned}
\text{All incidence} &= \text{community-based incidence spline} \\
\text{Hospitalization incidence} &= \text{community-based incidence spline} \\
&\quad \times \text{hospitalization probability spline} \\
\text{Incidence of hospital-based mortality} &= \text{community-based incidence spline} \\
&\quad \times \text{hospitalization probability spline} \\
&\quad \times \text{hCFR probability spline}
\end{aligned}$$

S1.4.2 OM II: hospital-based studies as a basis for incidence

For OM II, the basis of incidence calculations comes from hospital-based incidence studies:

$$\begin{aligned}
\text{All incidence} &= \frac{\text{hospital-based incidence spline}}{\text{hospitalization probability spline}} \\
\text{Hospitalization incidence} &= \text{hospital-based incidence spline} \\
\text{Incidence of hospital-based mortality} &= \text{hospital-based incidence spline} \\
&\quad \times \text{hCFR probability spline}
\end{aligned}$$

S1.5 Spline Models

Incidence Specifications

For incidence splines (splines I and II, the incidence of RSV based on community- and hospital-based studies, respectively) we assume a Poisson function:

$$\log(\texttt{cases}_i/\texttt{population}_i) = \alpha + \alpha_i + S(\log(\texttt{age})) + S_i(\log(\texttt{age}))$$

Where:

- α is the overall intercept (the probability of hospitalization or death at age 0 in any low- or middle-income country)
- α_i study-specific (i) random effect on the intercept
- $S(\log(\texttt{age}))$ a global trend (fixed-effect) spline for the correlation between the outcome and age, constructed with a thin-plate smooth with a penalty on the null space (shrunk to zero).
- $S_i(\log(\texttt{age}))$ a set of study-specific (i) random-effects splines constructed with a tensor product basis of a thin-plate smooth with a penalty on the null space (shrunk to zero) and a random-effects basis.

Traditionally, the population element is put on the right-hand side as a predictor constrained to have a coefficient equal to one (called an "offset"). This is the result of a property of log functions of ratios:

$$\log(\texttt{cases}_i) - \log(\texttt{population}_i) = \alpha + \alpha_i + S(\log(\texttt{age})) + S_i(\log(\texttt{age}))$$

$$\log(\texttt{cases}_i) = \alpha + \alpha_i + S(\log(\texttt{age})) + S_i(\log(\texttt{age})) + \text{offset}[\log(\texttt{population}_i)]$$

Incidence Splines - Income Group Stratification

We compared the null model above with one in which the age trend differs by World Bank income group classification:

$$\log(\texttt{cases}_i) = \alpha + \alpha_i + \beta_g + S_g(\log(\texttt{age})) + S_i(\log(\texttt{age})) + \text{offset}[\log(\texttt{population}_i)]$$

Where:

β_g	fixed-effect parametric coefficient terms for the indicator variables representing the World Bank income group g.
$S_g(\log(\texttt{age}))$	a trend (fixed-effect) spline for the correlation between the outcome and age, stratified by World Bank income group and constructed with a thin-plate smooth with a penalty on the null space (shrunk to zero).

As an analogy to the more familiar linear regression, the age and the income group interact so that the difference between the outcome across income groups is not uniform across ages, but rather that the relationship of age and the outcome is unique for each set of countries in an income group. It should be noted that the spline-fitting package we employed centered the factor-specific (income-group-specific) splines, and the authors advised that the factor also be added to the model as a main effect. For more information, see the documentation for R command `s()`, part of the `mgcv` package.

Additionally, we tested whether the possibility that the random effects (both on the intercept and on the spline) also differed by income group classification:

$$\log(\texttt{cases}_i) = \alpha + \alpha_{i,g} + \beta_g + S_g(\log(\texttt{age})) + S_{i,g}(\log(\texttt{age})) + \text{offset}[\log(\texttt{population}_i)]$$

There were two reasons that we ran alternative models with an interaction term rather than running separate models using data from each income group. First, our approach made it possible to do model comparisons between the models that did not stratify countries by income group (global models) are those that did (income-group models).

Model selection In order to select between the null model (no income group stratification) and the two models with income group stratification, we examined the χ^2 test of the deviance between models with degrees of freedom equal to the difference in the parameters between the two models. This is done via the likelihood ratio test shown with the command `anova(model1$mer, model2$mer)`.

Probability Splines

For probabilities splines (splines III and IV, the probability of hospitalization and the conditional probability of death among hospitalized cases, respectively), we assume a binomial process as follows:

$$\text{logit}(p_i) = \alpha + \alpha_i + S(\log(\texttt{age})) + S_i(\log(\texttt{age}))$$

Here, too, we compared the null model above with one in which the age trend differs by World Bank income group classification:

$$\text{logit}(p_i) = \alpha + \alpha_i + \beta_g + S_g(\log(\texttt{age})) + S_i(\log(\texttt{age}))$$

Additionally, we also tested the possibility that the random effects (both on the intercept and on the spline) also differed by income group classification:

$$\text{logit}(p_i) = \alpha + \alpha_{i,g} + \beta_g + S_g(\log(\texttt{age})) + S_{i,g}(\log(\texttt{age}))$$

In summary, model parameters are as follows:

α is the overall intercept (the probability of hospitalization or death at age 0 in any low- or middle-income country)

α_i study-specific (i) random effect on the intercept

$\alpha_{i,g}$ study-specific (i) random effect on the intercept stratified by World Bank income group (g)

β_g fixed-effect parametric coefficient terms for the World Bank income group g.

$S(\log(\text{age}))$ a global trend (fixed-effect) spline for the correlation between the outcome and age, constructed with a thin-plate smooth with a penalty on the null space (shrunk to zero).

$S_g(\log(\text{age}))$ a trend (fixed-effect) spline for the correlation between the outcome and age, stratified by World Bank income group and constructed with a thin-plate smooth with a penalty on the null space (shrunk to zero).

$S_i(\log(\text{age}))$ a set of study-specific (i) random-effects splines constructed with a tensor product basis of a thin-plate smooth with a penalty on the null space (shrunk to zero) and a random-effects basis.

$S_{i,g}(\log(\text{age}))$ a set of study-specific (i) random-effects splines stratified by World Bank income group and constructed with a tensor product basis of a thin-plate smooth with a penalty on the null space (shrunk to zero) and a random-effects basis.

offset$[\log(\texttt{population}_i)]$ predictor constrained to have a coefficient equal to one. In these models, it adjusts for the population of the study (i).

Below we describe the specifications for our model and our rationale for these choices.

S1.5.1 Spline specifications and computational considerations

Mapping discrete age data to a continuous number line. The age-specific data in the literature was presented by discrete age groups, but spline models would treat age as a continuous variable. For this reason, the midpoint of the age group was used to map onto the continuous real number line (i.e., observations of an age group of 0-3 months were considered to be the observations of children all aged 1.5 months).

Log-transforming the age variable. Studies in the literature tend to present the incidence of RSV at a relatively high resolution (narrow age bands) for children under the age of 12 or 24 months and for very broad age bands for children over 12 or 24 months of age. Estimating a spline model on such data (like on linear data) would run the risk that the data points of older age groups would have undue leverage over the spline.

Spline bases. We used a "shrinkage" variant of the thin-plate spline (`"ts"` in R), which "shrinks" the spline towards a straight line; in other words, the algorithm encourages shapes that are as close to a straight line as is sensible unless there is strong evidence that an alternative shape is warranted, thus avoiding overly complex curves to describe such diverse settings as the low- and middle-income countries. This was especially important for the probability of death (hCFR) splines, which were sometimes prone to unusual shapes to capture the few deaths that were observed in the data.

A tensor product basis was used to construct the random-effect splines specific to each study. Such a spline was the interaction of thin-plate splines and random-effect splines (spline basis `"re"` in R) in addition to random effect parametric terms that were assumed to be normally distributed (constituting a ridge penalty and specified by `random=~(EconomicSetting|studyno)`) [3, 6].

Number and placement of knots. We avoided choosing the ideal number of knots by using a shrinkage variant of the "shrinkage" version of the thin-plate spline, which places a knot at every data point and then estimates a "smoothing" parameter to avoid "unnecessary" inflections at each knot. See Section S1.3 of this supplement. The shrinkage version of the thin-plate smooth has a penalty on the null space to that the smoothing term is shrunk to zero.

It should be noted that we excluded studies that data for fewer than three age groups, as no age curve would be possible other than a straight line. However, we used these studies as an informal "out-of-sample validation" to visually assess how our splines would have predicted the incidence, hospitalization, and death from studies that were not used to estimate the spline models. The results for out-of-sample validations are in Sections S2-2.1-S2-2.4 in S2 Text.

Model comparison/variable selection. We selected the model according to the likelihood ratio test since the simpler models were nested within the more sophisticated models. The likelihood ratio test uses a χ^2 test

of the deviance with degrees of freedom equal to the difference in the parameters between the two models.

Family for each outcome. As described in section S1.3 of this supplement, we used regressions assuming a Poisson distribution of the outcome (with log-link) for the incidence splines (Splines I and II) and regressions assuming a binomial distribution of the outcome (with logit-link) for the probability splines (Splines III and IV).

As is common practice for Poisson models, overdispersion was checked (via the `check_overdispersion` command of the `performance` R package)[12]. While the community-based incidence splines (Spline I) showed no evidence of over-dispersion, the hospital-based incidence splines (Spline II) did, and so we modeled that spline with a negative binomial family of models - the same form as the Poisson function above but with an additional parameter modeling a larger variance than would be assumed with a Poisson family of models.

S1.5.2 Predictions of the outcome model and uncertainty

↩ Return to the Table of Contents.

We assumed that the posterior predictions of the splines would arise from a product of 1) a matrix of p parameters $\times$ n iterations (5,000) representing the random draws from a multivariate-normal distribution of the coefficients for the polynomial spline and 2) a matrix describing the estimated spline. The product is then exponentiated (for incidence outcomes estimated with a log-link) or transformed with an inverse-logit formula to the range of (0,1) (for probability outcomes estimated with a logit-link). When we combined the 5,000 splines as illustrated in Fig 1 and described in section S1.4 of this supplement, we were able to calculate the credible intervals of each of the outcomes by taking the median, mean, and the 2.5th and the 97.5th percentile of the outcome of interest.

S1.6 Burden Models (BM) I & II

↩ Return to the Table of Contents.

As shown in Fig 1 there are two ways to calculate the burden of disease in one country: 1) for Burden Model I, we apply the country population size to the incidences calculated in Spline Model I and II, and then we aggregate cases into age subgroups as desired; 2) for Burden Model II we take the number of cases under 5 in each country as calculated by Shi's risk-factor model, apply the proportion of cases that occur in each month of age according to our splines, and aggregate cases into age subgroups as desired. Because there are two ways to calculate age-specific incidence in part (A) and two ways to calculate burden in part (B), four sets of burden estimates result.

Shi and colleagues presented two models in their paper: one was the total number of cases that would be seen in each country if the incidence was equal to the mean incidence from the meta-analysis. We have re-calculated the analogous incidence estimates according to our spline-based meta-regression in burden model I (BM I).

A second model presented in the supplement predicted the incidence of RSV per country according to a regression model of RSV incidence with a variety of risk factors for the disease. Their risk-factor model of incidence, however, does not disaggregate by month of age), it only presents the total incidence for all children under 5.

S1.6.1 BM I: burden models using OM I and II and population estimates

In an analogous manner to Shi et al's main analysis, we took the results of our meta-regressions for OM I and OM II, applied an estimate of the population of each month of age for each country, and estimated the number of cases, hospitalizations, and inpatient deaths to each country.

Each country's population structure in children under 5 was assumed to be equal to that calculated by the UN population database, which contains data on the number of children at birth, at age 1, and at age 5 [13]. Therefore, the estimates of cases implicitly take into account the infant mortality in each country.

$$\text{BM I Cases (OM I)}_m^c = \text{All incidence spline (OM I)}_m \times \text{population}_m^c$$

$$\text{BM I Cases (OM II)}_m^c = \text{All incidence spline (OM II)}_m \times \text{population}_m^c$$

Age subgroup estimates are equal to the following:
Cases, hospitalizations, deaths within the hospital at 0-5.9 months old:

$$\text{BM I Cases (OM I)}_{0-5.9 \text{ months}}^c = \sum_0^5 \text{All incidence spline (OM I)}_m \times \text{population}_m^c dm$$

$$\text{BM I Cases (OM II)}_{0-5.9 \text{ months}}^c = \sum_0^5 \text{All incidence spline (OM II)}_m \times \text{population}_m^c dm$$

Cases, hospitalizations, deaths within the hospital at 6-11.9 months old:

$$\text{BM I Cases (OM I)}_{6-11.9 \text{ months}}^c = \sum_6^{11} \text{All incidence spline (OM I)}_m \times \text{population}_m^c dm$$

$$\text{BM I Cases (OM II)}_{6-11.9 \text{ months}}^c = \sum_6^{11} \text{All incidence spline (OM II)}_m \times \text{population}_m^c dm$$

Cases, hospitalizations, deaths within the hospital at 12-59.9 months old:

$$\text{BM I Cases (OM I)}_{12-59.9 \text{ months}}^c = \sum_{12}^{59} \text{All incidence spline (OM I)}_m \times \text{population}_m^c dm$$

$$\text{BM I Cases (OM II)}^{c}_{12-59.9\text{ months}} = \sum_{12}^{59} \text{All incidence spline (OM II)}_m \times \text{population}^{c}_{m} dm$$

To calculate cases, hospitalizations, deaths within the hospital for age groups 0-5.9 months old, 6-11.9 months old, and 12-59.9 months old, see below, section S1.6.3.

S1.6.2 BM II: burden models combining the OM I & II, Shi et al's risk factor model of RSV incidence among all children under 5, and population estimates

We have also made age-specific incidence estimates that integrated the estimates of RSV incidence for children under 5 in Shi's country-level risk factor model. It should be noted that our estimate of total cases under 5 was not different than theirs, but we re-apportioned cases within the age groups such that their sum would equal Shi's estimate of cases. Our basis for apportioning cases to different age groups was our outcome models, as illustrated in Fig 1.

Like in BM I, we have two ways of apportioning cases, according to OM I and OM II, so therefore there are two versions of BM II as well:

$$\text{BM II Cases(OM I)}^{c}_{m} = \text{Risk-factor-model Incidence}^{c}_{0\text{-}59\text{ months}} \times \text{Proportion of cases(OM I)}_m \times \text{population}^{c}_{m}$$

$$\text{BM II Cases(OM II)}^{c}_{m} = \text{Risk-factor-model Incidence}^{c}_{0\text{-}59\text{ months}} \times \text{Proportion of cases(OM II)}_m \times \text{population}^{c}_{m}$$

Where:

- c is a specific country
- m is the age in months
- Risk-factor-model $\text{Incidence}^{c}_{0\text{-}59\text{ months}}$ is the incidence of all children from 0-59 months of age in Shi's risk factor model of incidence for children of that age group.
- Proportion of cases in each month of age, according to OM I:

$$\text{Proportion of cases (OM I)}_m = \frac{\text{All incidence spline (OM I)}_m \times \text{population}^{c}_{m}}{\sum_m \text{All incidence spline (OM I)}_m \times \text{population}^{c}_{m} dm}$$

- Proportion of cases in each month of age, same as above, but according to OM II instead of OM I:

$$\text{Proportion of cases (OM II)}_m = \frac{\text{All incidence spline (OM II)}_m \times \text{population}^{c}_{m}}{\sum_m \text{All incidence spline (OM II)}_m \times \text{population}^{c}_{m} dm}$$

- $\text{population}^{c}_{m}$ is the population in country c at m months of age

To calculate cases, hospitalizations, and deaths within the hospital for age groups 0-5.9 months old, 6-11.9 months old, and 12-59.9 months old, we aggregated each of these outcomes across as described for BM I, as detailed in section S1.6.1 of this supplement.

Furthermore, in addition to presenting the age-specific case estimates, we presented the number of hospitalizations and inpatient deaths that would result from a model that assumes the underlying community incidence of RSV is equivalent to Shi's et al's risk factor model.

S1.6.3 Burden models: computational considerations and uncertainty calculations

Approximating integrals. To calculate the number of cases, hospitalizations, and inpatient deaths in each age month, we approximated the integrals with respect to m, rather than calculating it analytically. We had to take this approach because we had to take into account that our splines were predicted at discrete ages rather than as a distinct mathematical expression that could be integrated. However, we judged that calculating incidence, hospitalization, and death at age intervals of 0.1 months (600 intervals between ages 0-59.9) gave us a sufficient resolution to approximate the integrals shown above.

Uncertainty in BM I. In order to calculate the uncertainty intervals of the cases, hospitalizations, and inpatient deaths described in the burden models for each country and groups of countries, we took the uncertainty in each of the outcome models, calculated as described in section S1.5.2 of this supplement. For BM I, the uncertainty in the burden estimates arose completely from the outcome models, as we fixed the population of

each country such that no uncertainty was derived from the size of the population at risk. We considered this a sensible choice as the populations for most countries are known with a high degree of accuracy compared to the uncertainty in RSV outcomes.

Uncertainty in BM II. For BM II, there is uncertainty from two domains: 1) the outcomes models, from which the uncertainty estimation is described in section S1.5.2 of this supplement, and 2) the uncertainty in the estimates of RSV cases in the community from Shi and colleagues' country-level risk factor model. To match the 5,000 samples of our splines, we drew 5,000 samples from a log-normal distribution described by the log-mean of the incidence and the standard deviation on the log-scale that would result in the 95% confidence intervals that were reported. We applied the sample vector of cases from the risk-factor model to each sample of our splines as described in section S1.6.2 of this supplement, resulting in a sample vector for each of the outcomes of BM II. We could then take the median and 95% credible intervals of the sample vectors of BM II to report in the results.

S1.7 Characterizing the age profile of RSV burden (mean, median, and the peak age of each outcome)

↩ Return to the Table of Contents.

For each iteration, we calculate the mean age of cases per iteration by taking a vector of the ages and weighting by the proportion of cases that occurs at each age. The median age per iteration was done by calculating the age at which half of all cases would have already occurred (usually close to the mean age, except when there is a long right tail in the distribution of the cases.) The peak age was calculated by taking the age at which incidence is highest in each iteration of the outcome models. For all measures (mean, median, and peak age) we also performed the analogous approach for hospitalizations and inpatient deaths. Then the 95% credible intervals for each of the mean, median, and peak ages are calculated by taking each of these measures in each iteration and calculating the median and 2.5% and 97.5% percentiles of each.

S1.8 Severe and very severe disease

↩ Return to the Table of Contents.

'Severe' or 'very severe' RSV disease is defined by the WHO Integrated Management of Childhood Illnesses (IMCI) [14].

- **RSV-associated severe ALRI**: cough or difficulty in breathing with chest wall indrawing and laboratory-confirmed RSV; children aged <2 months, increased RR (>60 breaths/ min) OR chest wall indrawing and laboratory-confirmed RSV
- **RSV-associated very severe ALRI**: cough or difficulty in breathing with one danger sign (cyanosis, difficulty in breastfeeding or drinking, vomiting everything, convulsions, lethargy, or unconsciousness, head nodding)
- **Hospitalised RSV-associated severe ALRI**: hospitalized ALRI cases with hypoxemia (as defined below) and laboratory-confirmed RSV
- **Hospitalised RSV-associated very severe ALRI**: hospitalized ALRI with one danger sign (cyanosis, difficulty in breastfeeding or drinking, vomiting everything, convulsions, lethargy, or unconsciousness, head nodding) OR proxies for very severe disease – mechanical ventilation OR ICU admission.

To explore the window of protection against severe and very severe RSV disease, we devised a model that connects to the model of disease depicted in Fig 1. The model integrating severity is shown in Fig B of this supplement. As can be seen here, the connection between severe disease and death is not clear, and therefore we have not integrated severity into our main model. This is because severity is not always a pre-condition to hospitalization, and hospitalization is not necessarily an obligation in the context of severe cases; a physician's overall impression of the child plays a role, and in some contexts, wealth, access, and space also play a role on whether a severe case is hospitalized.

Another important note is that although very severe disease could be calculated conditional on the severe disease of community-based surveillance, severe and very severe disease was not related in the studies of hospital-based surveillance. Therefore, among studies of hospital-based surveillance, the very severe probability is calculated conditional on hospitalization, but not on (non-very) severe disease. The reason for this is that severe hospitalized disease includes hypoxemia, but very severe disease could include other 'danger signs' as defined by the WHO IMCI (see above definitions and Shi et al's supplement page 85 [15]).

For OM I, the basis of incidence calculations come from community-based incidence studies:

$$
\begin{aligned}
\text{All incidence} &= \text{community-based incidence spline}\\
\text{All incidence - severe cases} &= \text{community-based incidence spline}\\
&\quad \times \text{probability of severe RSV spline}\\
\text{All incidence - very severe cases} &= \text{community-based incidence spline}\\
&\quad \times \text{probability of severe RSV spline}\\
&\quad \times \text{probability of very severe RSV spline}\\
\text{Hospitalization incidence} &= \text{community-based incidence spline}\\
&\quad \times \text{hospitalization probability spline}\\
\text{Hospitalization incidence - severe cases} &= \text{community-based incidence spline}\\
&\quad \times \text{hospitalization probability spline}\\
&\quad \times \text{probability of severe RSV among the hospitalized spline}\\
\text{Hospitalization incidence - very severe cases} &= \text{community-based incidence spline}\\
&\quad \times \text{hospitalization probability spline}\\
&\quad \times \text{probability of very severe RSV among the hospitalized spline}
\end{aligned}
$$

S1.8.1 OM II: hospital-based studies as a basis for incidence

For OM II, the basis of incidence calculations comes from hospital-based incidence studies:

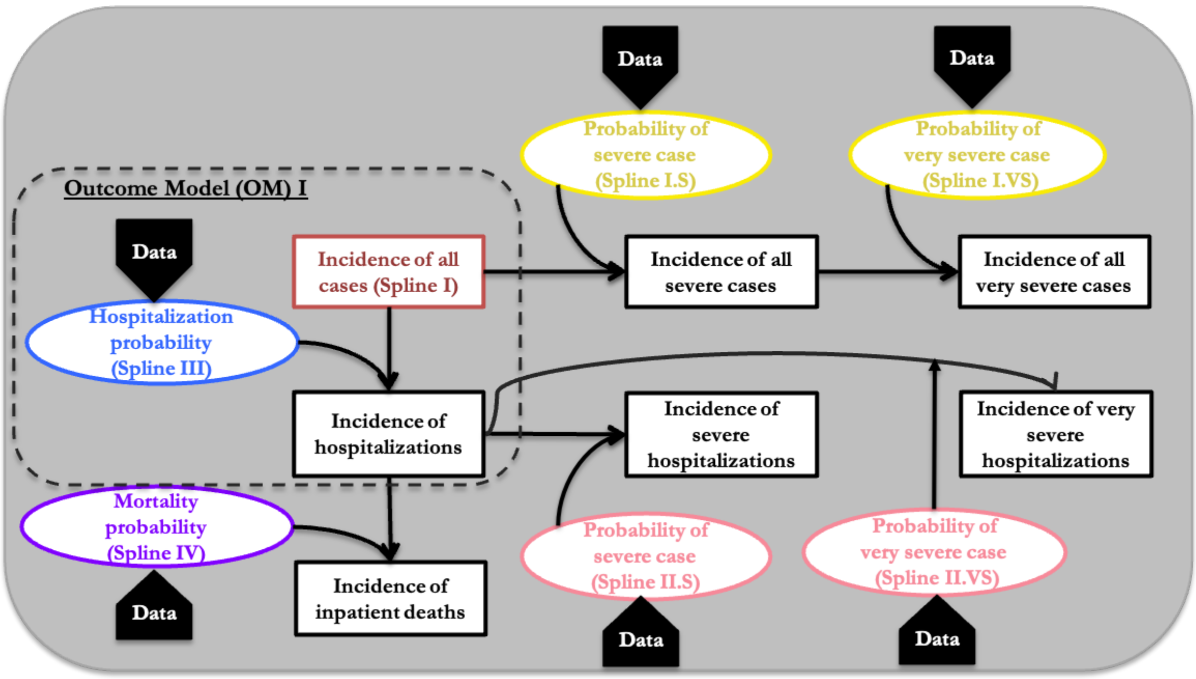

Figure B: **Relationship between cases, hospitalizations, and severity in children from birth to 59 months of age.** The boxes show incidence and the ovals show the probability of progressing from a case in the community to a case in the hospital, or from a case in the hospital to a fatal case, and the probability that community or hospital cases are severe or very severe. It should be noted that the product of incidence and probability yields an incidence. The colored boxes show the splines (splines I-IV) that we estimated from data in the literature, and the black boxes show incidence derived as products of our splines. In OM I, we use the incidence of community-acquired RSV cases (spline I) and the probabilities of hospitalization (spline III) and we derive the incidence of hospitalizations. In OM II (see figure below) we use the incidence of RSV cases from hospital-based studies (spline II) and the probabilities of hospitalization to back-calculate the community-based incidence; the severe and very severe cases were then calculated in a manner analogous to that in OM I. From these spline models, shown in Fig B and C in S3 Text we also estimated the peak, median, and mean age of infection, shown Fig D in S3 Text.

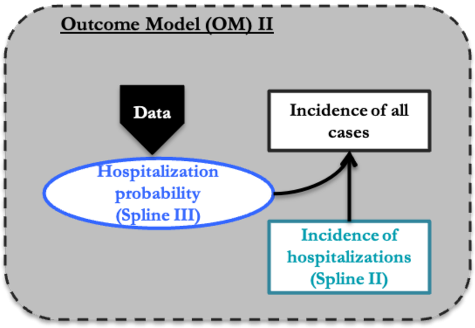

$$\text{All incidence} = \frac{\text{hospital-based incidence spline}}{\text{hospitalization probability spline}}$$

$$\text{All incidence} = \frac{\text{hospital-based incidence spline}}{\text{hospitalization probability spline}} \times \text{probability of severe RSV spline}$$

$$\text{All incidence} = \frac{\text{hospital-based incidence spline}}{\text{hospitalization probability spline}} \times \text{probability of severe RSV} \times \text{probability of very severe RSV spline}$$

$$\begin{aligned}
\text{Hospitalization incidence} &= \text{hospital-based incidence spline} \\
\text{Hospitalization incidence} &= \text{hospital-based incidence spline} \\
&\times \text{probability of severe RSV among the hospitalized spline} \\
\text{Hospitalization incidence} &= \text{hospital-based incidence spline} \\
&\times \text{probability of very severe RSV among the hospitalized spline}
\end{aligned}$$

S1.9 Countries included, World Bank Income Group, population, and life tables

↩ Return to the Table of Contents.

We gathered the income groups for 2020 from the World Bank [16], the population and the population and the life table information from the UN Population Prospects interfacing via the `wpp2019` R package (version 1.1-1) [13, 17].

Income Group	Countries	Age 0-5m	Age 6-11m	Age 12-59m	Population
LIC	27	10,724,862	10,493,726	79,974,656	102,833,033
LMIC	53	34,374,937	33,838,946	261,805,123	335,421,725
UMIC	41	16,005,002	15,920,633	125,996,828	160,540,706
**Total**	**121**	**61,088,015**	**60,242,855**	**467,802,550**	**598,795,464**

Table B: Country income groups and population age <5 in 2020.

Countries that were in Li's risk factor model but are HIC and excluded from our analysis. The difference of 8.3M constitutes less than a 1.5% difference in population, so we do not believe it drives the differences in our results.

ISO3	Country	Population 2020	RSV cases
ATG	Antigua and Barbuda	7,355	239
BHS	Bahamas, The	27,064	1,274
BHR	Bahrain	108,054	5,488
BRB	Barbados	15,120	733
BRN	Brunei Darussalam	31,672	1,598
CHL	Chile	1,162,223	56,448
KOR	Korea, Rep.	1,897,011	91,147
KWT	Kuwait	289,689	14,811
OMN	Oman	453,666	22,331
QAT	Qatar	135,162	6,427
SAU	Saudi Arabia	2,978,337	150,348
SYC	Seychelles	7,967	534
SGP	Singapore	258,176	1,261
TTO	Trinidad and Tobago	88,366	4,577
ARE	United Arab Emirates	499,138	24,639
URY	Uruguay	236,656	11,554
	Total	8,195,656	393,409

Table C: Population in countries that were 'developing' according to UNICEF classifications but were high-income countries according to the World Bank's lending groups. These were included in Li et al's analysis but excluded in our analysis.

Countries that were LIC, LMIC, or UMIC but were not in Li's risk factor analysis. For comparability, we've excluded them from our analysis as well. However, this is a 5% difference in the population of interest and is largely concentrated in UMICs.

ISO3	Country name	Income Group	Population, 2020
ALB	Albania	UM	166,278
BLR	Belarus	UM	548,121
BIH	Bosnia & Herzegovina	UM	133,254
BGR	Bulgaria	UM	312,730
MKD	North Macedonia	UM	111,883
MDA	Moldova	UM	202,520
MNE	Montenegro	UM	36,906
ROU	Romania	UM	939,606
RUS	Russia	UM	9,271,691
SRB	Serbia	UM	419,057
UKR	Ukraine	LM	2,113,850
PSE	Palestinian Territories	LM	692,913
		Total	14,948,809

Table D: Countries missing from the risk-factor model by Li et al that were LIC/LMIC/UMIC countries in 2020. To make estimates comparable, these studies were also not included in our analysis.

References

[1] Nair H. Global, regional and national disease burden estimates of acute lower respiratory infections due to respiratory syncytial virus in young children in 2015, 1995-2015 [dataset]; 2017. Available from: https://datashare.ed.ac.uk/handle/10283/2115 [cited 20-08-2022].

[2] Li Y, Nair H. Global, regional, and national disease burden estimates of acute lower respiratory infections due to respiratory syncytial virus in young children in 2019: a systematic analysis, 2017-2020 [dataset]; 2021. Available from: https://datashare.ed.ac.uk/handle/10283/4025 [cited 20-08-2022].

[3] Wood SN. Generalized Additive Models. Chapman and Hall/CRC; 2017. Available from: https://linkinghub.elsevier.com/retrieve/pii/S0140673617309388https://www.taylorfrancis.com/books/9781498728348.

[4] James G, Witten D, Hastie T, Tibshirani R. An Introduction to Statistical Learning with Applications in R. Springer Texts in Statistics. New York, NY: Springer New York; 2013. Available from: http://link.springer.com/10.1007/978-1-4614-7138-7.

[5] Wood SN, Pya N, Säfken B. Smoothing Parameter and Model Selection for General Smooth Models. Journal of the American Statistical Association. 2016;111(516):1548–1563. doi:10.1080/01621459.2016.1180986.

[6] Wood SN. {mgcv}: Mixed GAM Computation Vehicle with Automatic Smoothness Estimation; 2022. Available from: https://cran.r-project.org/package=mgcv [cited 01-08-2022].

[7] Troeger C, Blacker B, Khalil IA, Rao PC, Cao J, Zimsen SRM, et al. Estimates of the global, regional, and national morbidity, mortality, and aetiologies of lower respiratory infections in 195 countries, 1990–2016: a systematic analysis for the Global Burden of Disease Study 2016. The Lancet Infectious Diseases. 2018;18(11):1191–1210. doi:10.1016/S1473-3099(18)30310-4.

[8] Flaxman AD, Vos T, Murray CJ. An Integrative Metaregression Framework for Descriptive Epidemiology. University of Washington Press; 2015.

[9] Stijnen T, Hamza TH, Özdemir P. Random effects meta-analysis of event outcome in the framework of the generalized linear mixed model with applications in sparse data. Statistics in Medicine. 2010;29(29):3046–3067. doi:10.1002/sim.4040.

[10] Viechtbauer W. Conducting Meta-Analyses in R with the metafor Package. Journal of Statistical Software. 2010;36(3):1–48. doi:10.18637/jss.v036.i03.

[11] Antillón M, Warren JL, Crawford FW, Weinberger DM, Kürüm E, Pak GD, et al. The burden of typhoid fever in low- and middle-income countries: A meta-regression approach. PLOS Neglected Tropical Diseases. 2017;11(2):e0005376. doi:10.1371/journal.pntd.0005376.

[12] Lüdecke D, Ben-Shachar MS, Patil I, Waggoner P, Makowski D. {performance}: An {R} Package for Assessment, Comparison and Testing of Statistical Models. Journal of Open Source Software. 2021;6(60):3139. doi:10.21105/joss.03139.

[13] United Nations Population Division. World Population Prospects 2019; 2020. Available from: https://population.un.org/wpp/ [cited 01-02-2020].

[14] World Health Organization. Integrated Management of Childhood Illness (IMCI): Chart Booklet. March. WHO Press; 2014.

[15] Shi T, McAllister DA, O'Brien KL, Simoes EAF, Madhi SA, Gessner BD, et al. Global, regional, and national disease burden estimates of acute lower respiratory infections due to respiratory syncytial virus in young children in 2015: a systematic review and modelling study. The Lancet. 2017;390(10098):946–958. doi:10.1016/S0140-6736(17)30938-8.

[16] World Bank. The World by Income and Region; 2017. Available from: https://datatopics.worldbank.org/world-development-indicators/the-world-by-income-and-region.html [cited 11-01-2022].

[17] United Nations Population Division. wpp2019: World Population Prospects 2019; 2020. Available from: https://cran.r-project.org/package=wpp2019 [cited 01-02-2020].
