# Supplementary material for: The age profile of respiratory syncytial virus burden in preschool children of low- and middle-income countries: A semi-parametric, meta-regression approach
Source: PLoS Med. 2023 Jul 17;20(7):e1004250. doi: 10.1371/journal.pmed.1004250 (PMC10389726; doi:10.1371/journal.pmed.1004250)
Supplement: S2 Text — Section 2–1.1. Fit-vs-observed: Community-based incidence. Section 2–1.2. Fit-vs-observed: Hospital-based incidence. Section 2–1.3. Fit-vs-observed: Probability of hospitalization among cases in the community. Section 2–1.4. Fit-vs-observed: Probability of death among hospitalized cases. Section 2–2.1. Out-of-sample validation: Community-based incidence. Section 2–2.2. Out-of-sample validation: Hospital-based incidence. Section 2–2.3. Out-of-sample validation: Probability of hospitalization among cases in the community. Section 2–2.4. Out-of-sample validation: Probability of death among hospitalized cases. (PDF) [file pmed.1004250.s002.pdf]

Supporting Information

The age profile of respiratory syncytial virus burden in pre-school children of low- and middle-income countries: A semi-parametric, meta-regression approach

S2. Supplementary Results: Spline Validation

Contents

S2-1 Spline model predictions versus observations: within-sample validation S2-3
- S2-1.1 Fit-vs-observed: Community-based incidence S2-3
- S2-1.2 Fit-vs-observed: Hospital-based incidence S2-5
- S2-1.3 Fit-vs-observed: Probability of hospitalization among cases in the community S2-10
- S2-1.4 Fit-vs-observed: Probability of death among hospitalized cases S2-11

S2-2 Spline model predictions versus observations: out-of-sample validation. S2-18
- S2-2.1 Out-of-sample validation: Community-based incidence S2-18
- S2-2.2 Out-of-sample validation: Hospital-based incidence S2-19
- S2-2.3 Out-of-sample validation: Probability of hospitalization among cases in the community S2-22
- S2-2.4 Out-of-sample validation: Probability of death among hospitalized cases S2-23

S2-3 Spline model predictions versus observations: within-sample validation for severity outcomes . S2-30
- S2-3.1 Fit-vs-observed: Probability of severe cases among community-based incidence studies S2-30
- S2-3.2 Fit-vs-observed: Probability of very severe cases among community-based incidence studies S2-32
- S2-3.3 Fit-vs-observed: Probability of severe cases among hospital-based incidence studies . . S2-33
- S2-3.4 Fit-vs-observed: Probability of very severe cases among hospital-based incidence studiesS2-35

List of Figures

- A Spline model predictions versus observed estimates of community-based incidence in lower-middle-income countries (LMICs) S2-3
- B Spline model predictions versus observed estimates of community-based incidence S2-4
- C Spline model predictions versus observed estimates of hospital-based incidence in LIC settings S2-5
- D Spline model predictions versus observed estimates of hospital-based incidence in LMIC settingsS2-6
- E Spline model predictions versus observed estimates of hospital-based incidence in LMIC settingsS2-7
- F Spline model predictions versus observed estimates of hospital-based incidence in UMIC settingsS2-8
- G Spline model predictions versus observed estimates of hospital-based incidence in UMIC settingsS2-9
- H Spline model predictions versus observed estimates of the probability of hospitalization among cases in the community (all income groups) S2-10
- I Spline model predictions versus observed estimates of the probability of death among hospitalized cases in LIC settings S2-11
- J Spline model predictions versus observed estimates of the probability of death among hospitalized cases in LMIC settings S2-12
- K Spline model predictions versus observed estimates of the probability of death among hospitalized cases in LMIC settings S2-13
- L Spline model predictions versus observed estimates of the probability of death among hospitalized cases in LMIC settings S2-14
- M Spline model predictions versus observed estimates of the probability of death among hospitalized cases in UMIC settings S2-15
- N Spline model predictions versus observed estimates of the probability of death among hospitalized cases in UMIC settings S2-16
- O Spline model predictions versus observed estimates of the probability of death among hospitalized cases in UMIC settings S2-17
- P Out-of-sample validation: Spline model predictions versus observed estimates of community-based incidence in settings that were not used to construct the incidence spline. S2-18

Q Out-of-sample validation: Spline model predictions versus hospital-based incidence in LIC settings S2-19
R Out-of-sample validation: Spline model predictions versus observed estimates of hospital-based incidence in LMIC settings . . . S2-20
S Out-of-sample validation: Spline model predictions versus observed estimates of hospital-based incidence in UMIC settings . . . S2-21
T Out-of-sample validation: Spline model predictions versus observed estimates of the probability of hospitalization among cases in the community (all income groups) . . . S2-22
U Out-of-sample validation: Spline model predictions versus observed estimates of the probability of death among hospitalized cases in LIC settings that were not used to construct the probability spline . . . S2-23
V Out-of-sample validation: Spline model predictions versus observed estimates of the probability of death among hospitalized cases in LMIC settings that were not used to construct the probability spline . . . S2-24
W Out-of-sample validation: Spline model predictions versus observed estimates of the probability of death among hospitalized cases in LMIC settings that were not used to construct the probability spline . . . S2-25
X Out-of-sample validation: Spline model predictions versus observed estimates of the probability of death among hospitalized cases in LMIC settings that were not used to construct the probability spline . . . S2-26
Y Out-of-sample validation: Spline model predictions versus observed estimates of the probability of death among hospitalized cases in LMIC settings that were not used to construct the probability spline . . . S2-27
Z Out-of-sample validation: Spline model predictions versus observed estimates of the probability of death among hospitalized cases in UMIC settings that were not used to construct the probability spline . . . S2-28
AA Out-of-sample validation: Spline model predictions versus observed estimates of the probability of death among hospitalized cases in UMIC settings that were not used to construct the probability spline . . . S2-29
AB Spline model predictions versus observed estimates of the probability of severity among cases in community-based studies . . . S2-30
AC Spline model predictions versus observed estimates of the probability of severity among cases in community-based studies . . . S2-31
AD Spline model predictions versus observed estimates of the probability of very severe cases among cases in community-based studies . . . S2-32
AE Spline model predictions versus observed estimates of the probability of severe cases among cases in hospital-based studies . . . S2-33
AF Spline model predictions versus observed estimates of the probability of severity among cases in hospital-based studies . . . S2-34
AG Spline model predictions versus observed estimates of the probability of very severe cases among cases in hospital-based studies in LICs . . . S2-35
AH Spline model predictions versus observed estimates of the probability of very severe cases among cases in hospital-based studies in LMICs . . . S2-36
AI Spline model predictions versus observed estimates of the probability of very severe cases among cases in hospital-based studies in UMICs . . . S2-37

S2-1 Spline model predictions versus observations: within-sample validation

S2-1.1 Fit-vs-observed: Community-based incidence

↩ Return to the Table of Contents.

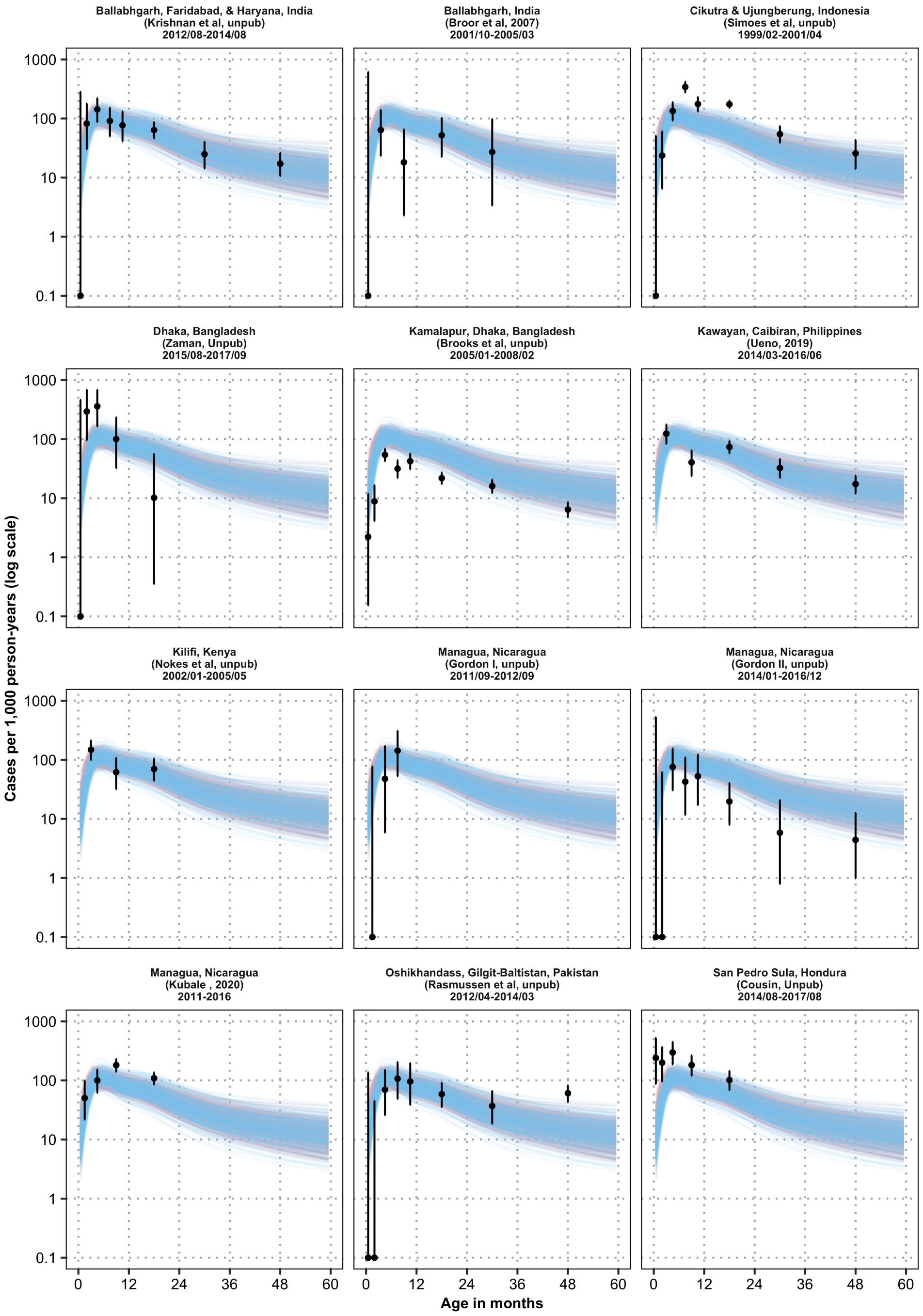

Figure A: Spline model predictions versus observed estimates of community-based incidence in lower-middle-income countries (LMICs). Each of the lines represents a random model prediction. The observed incidence estimated from each age group is placed at the midpoint of the age group, and the bars represent the 95% confidence interval of the incidence in that age group.

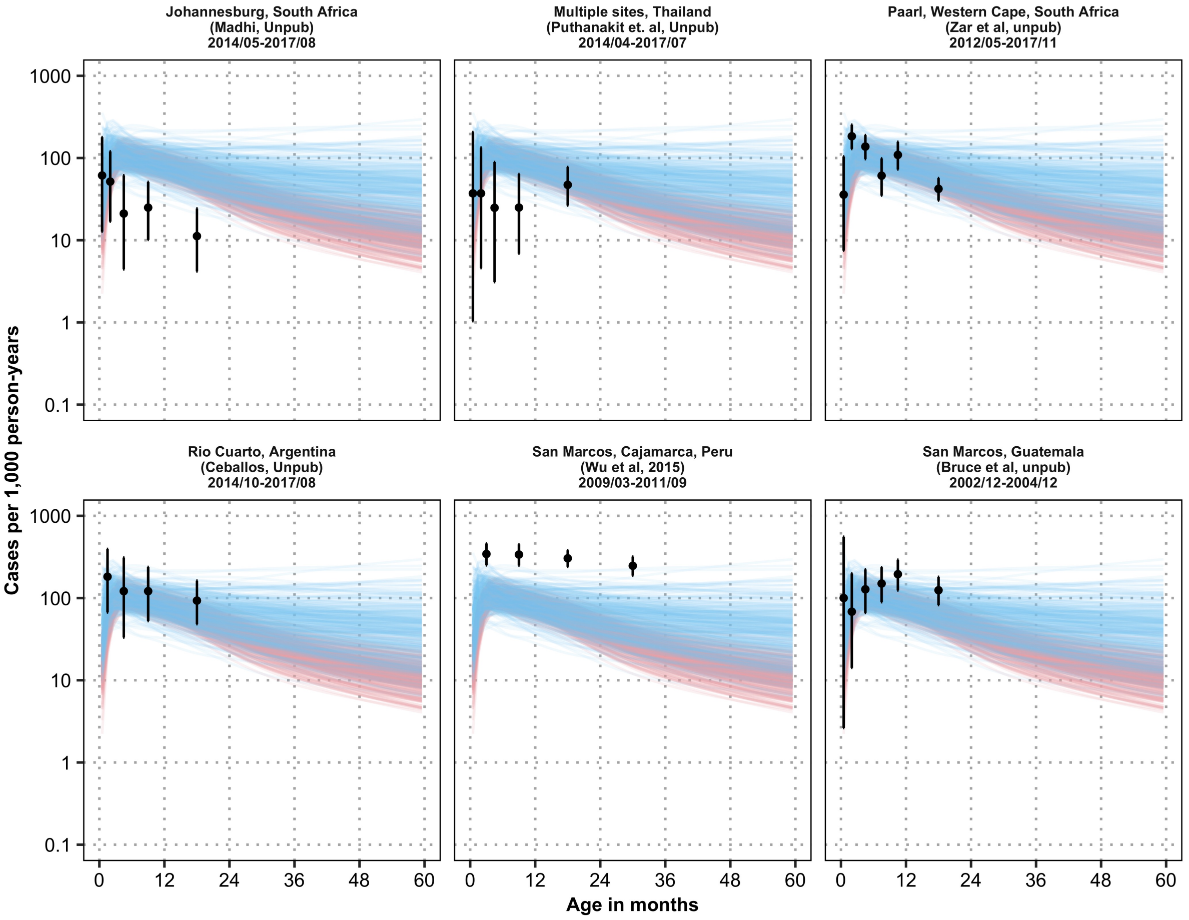

Figure B: Spline model predictions versus observed estimates of community-based incidence. Each of the lines represents a random model prediction. The observed incidence estimated from each age group is placed at the midpoint of the age group, and the bars represent the 95% confidence interval of the incidence in that age group.

S2-1.2 Fit-vs-observed: Hospital-based incidence

↩ Return to the Table of Contents.

Low-income countries (LIC)

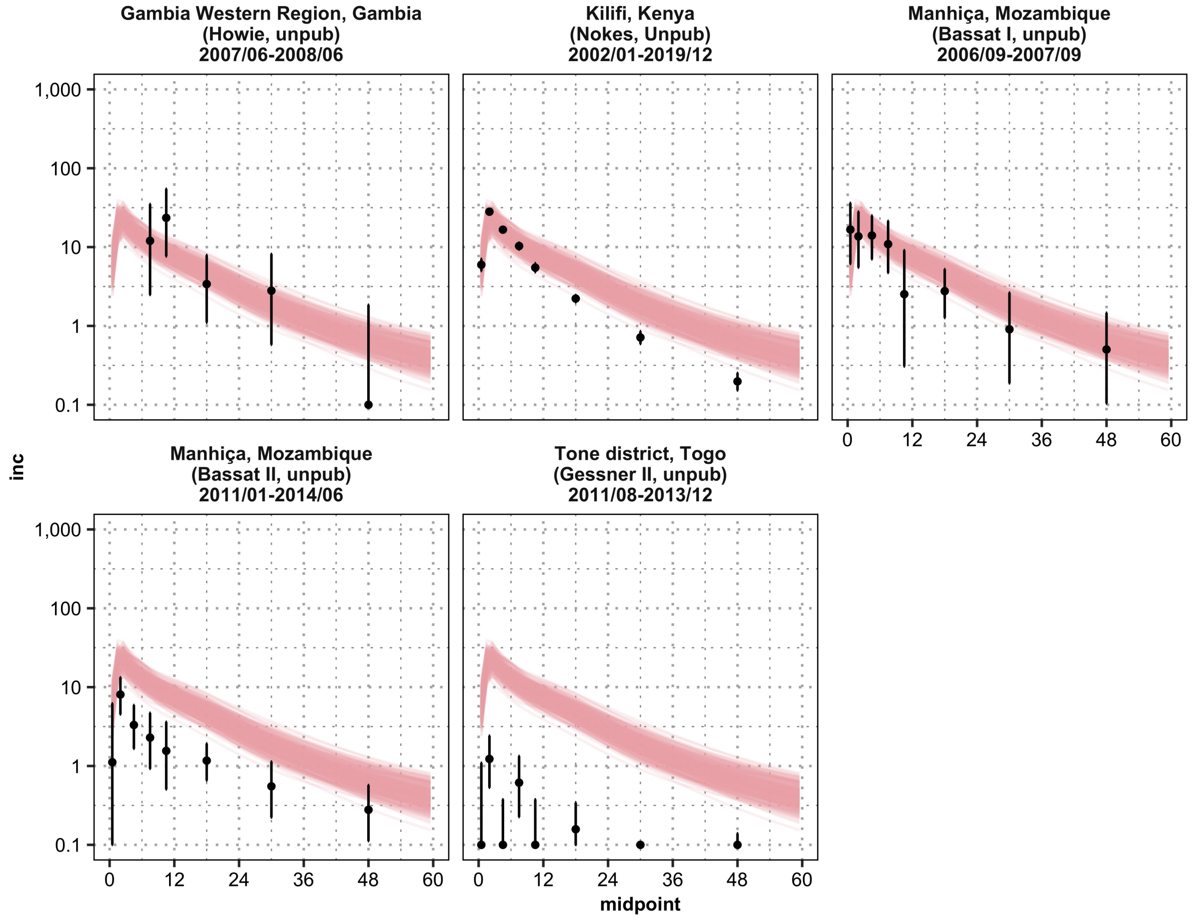

Figure C: Spline model predictions versus observed estimates of hospital-based incidence in LIC settings. Each of the lines represents a random model prediction. The pink lines represent predictions from the model estimated without a predictor for the country-level income group and the blue lines represent a model with the income group as a predictor. The observed incidence estimated from each age group is placed at the midpoint of the age group, and the bars represent the 95% confidence interval of the incidence in that age group.

Lower middle-income countries (LMIC)

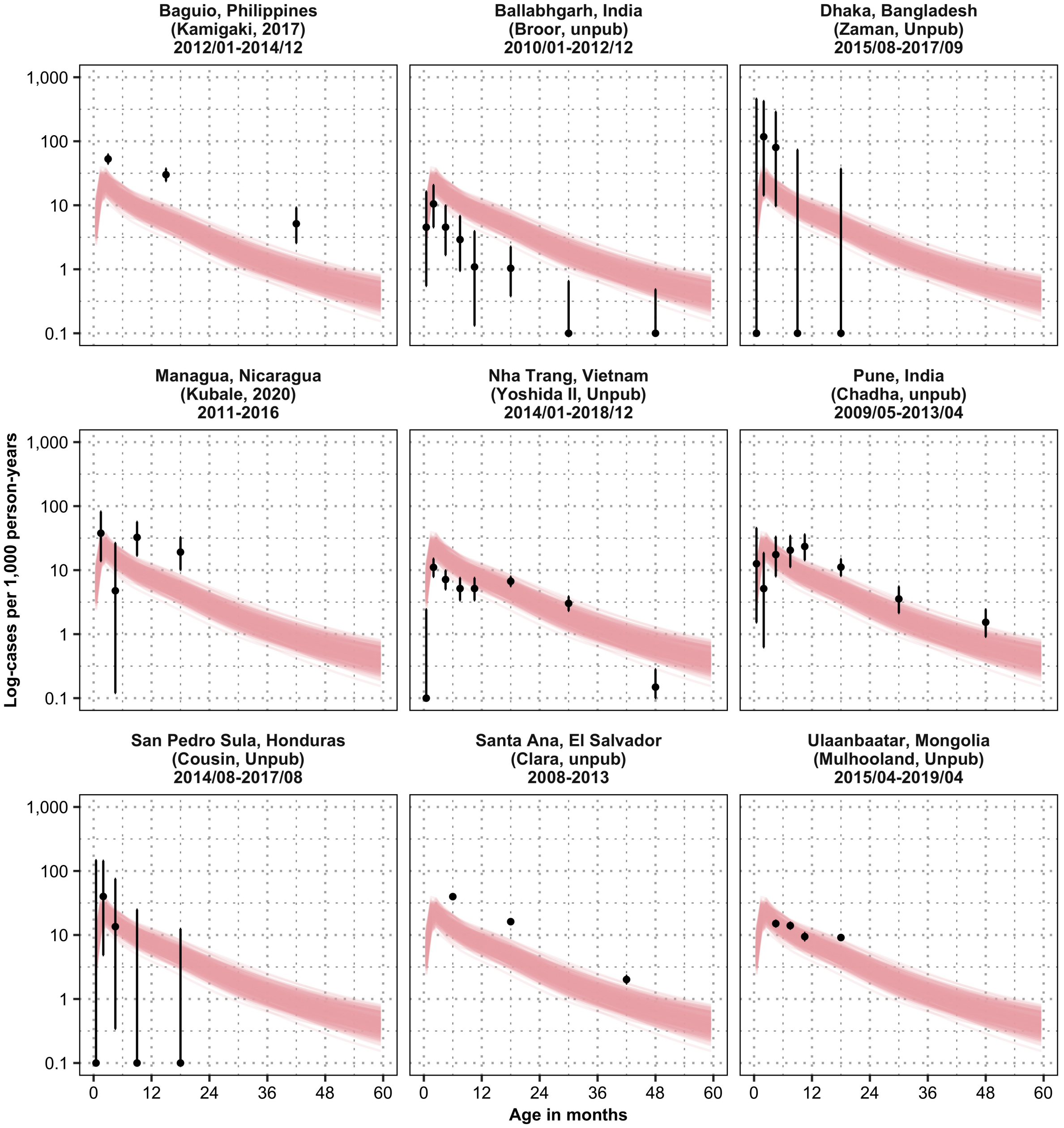

Figure D: Spline model predictions versus observed estimates of hospital-based incidence in LMIC settings. Each of the lines represents a random model prediction. The observed incidence estimated from each age group is placed at the midpoint of the age group, and the bars represent the 95% confidence interval of the incidence in that age group.

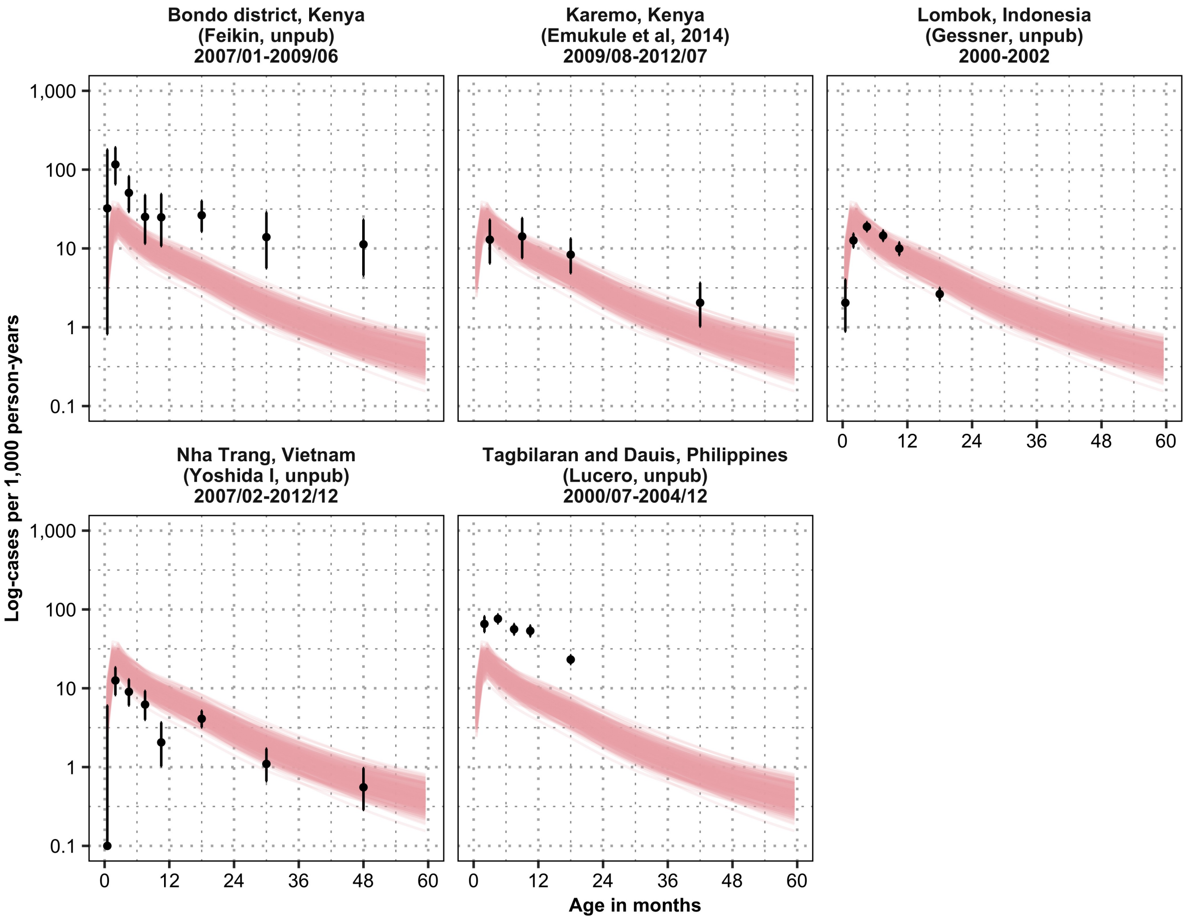

Figure E: Spline model predictions versus observed estimates of hospital-based incidence in LMIC settings. Each of the lines represents a random model prediction. The observed incidence estimated from each age group is placed at the midpoint of the age group, and the bars represent the 95% confidence interval of the incidence in that age group.

Upper middle-income countries (UMIC)

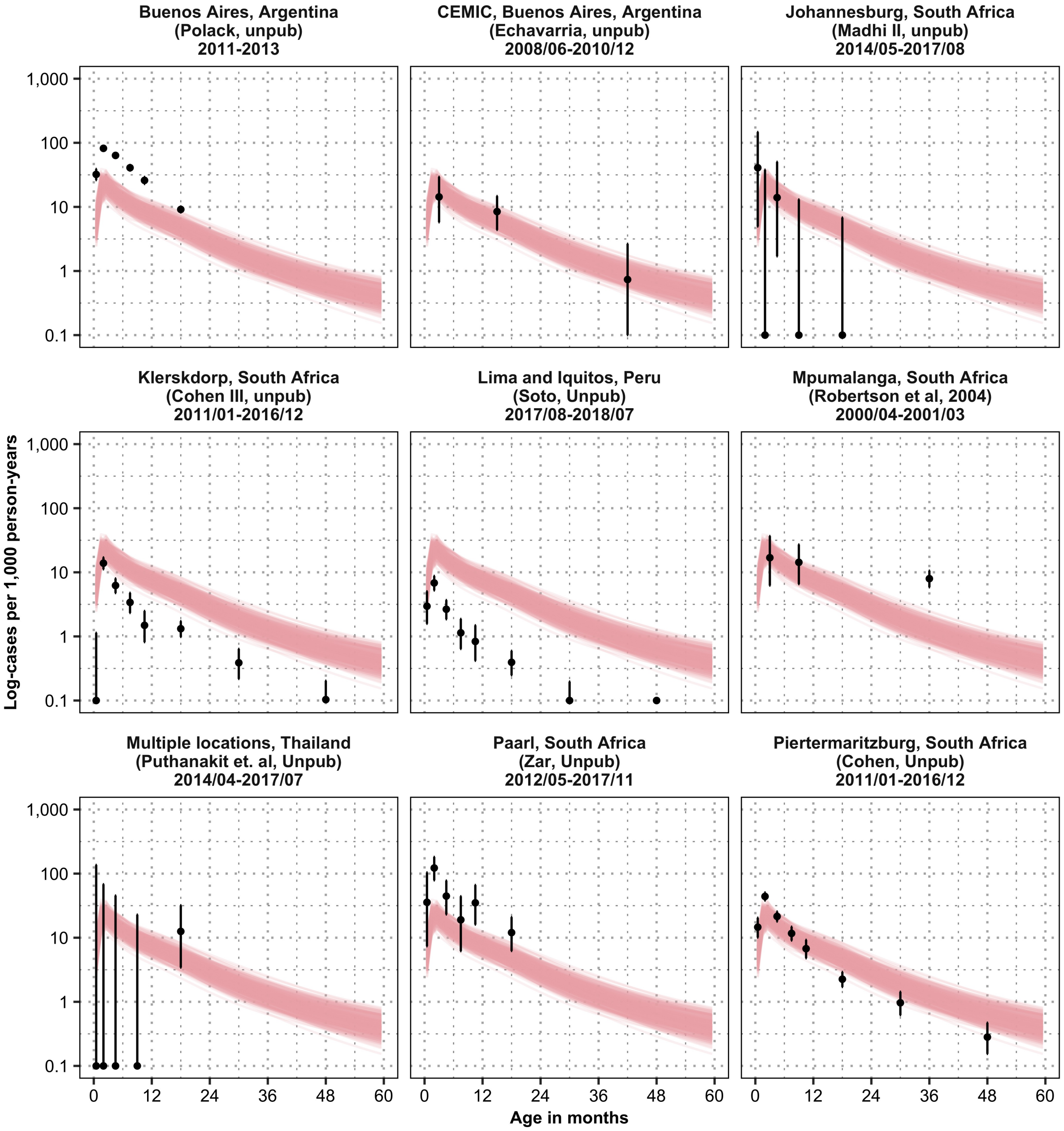

Figure F: Spline model predictions versus observed estimates of hospital-based incidence in UMIC settings. Each of the lines represents a random model prediction. The observed incidence estimated from each age group is placed at the midpoint of the age group, and the bars represent the 95% confidence interval of the incidence in that age group.

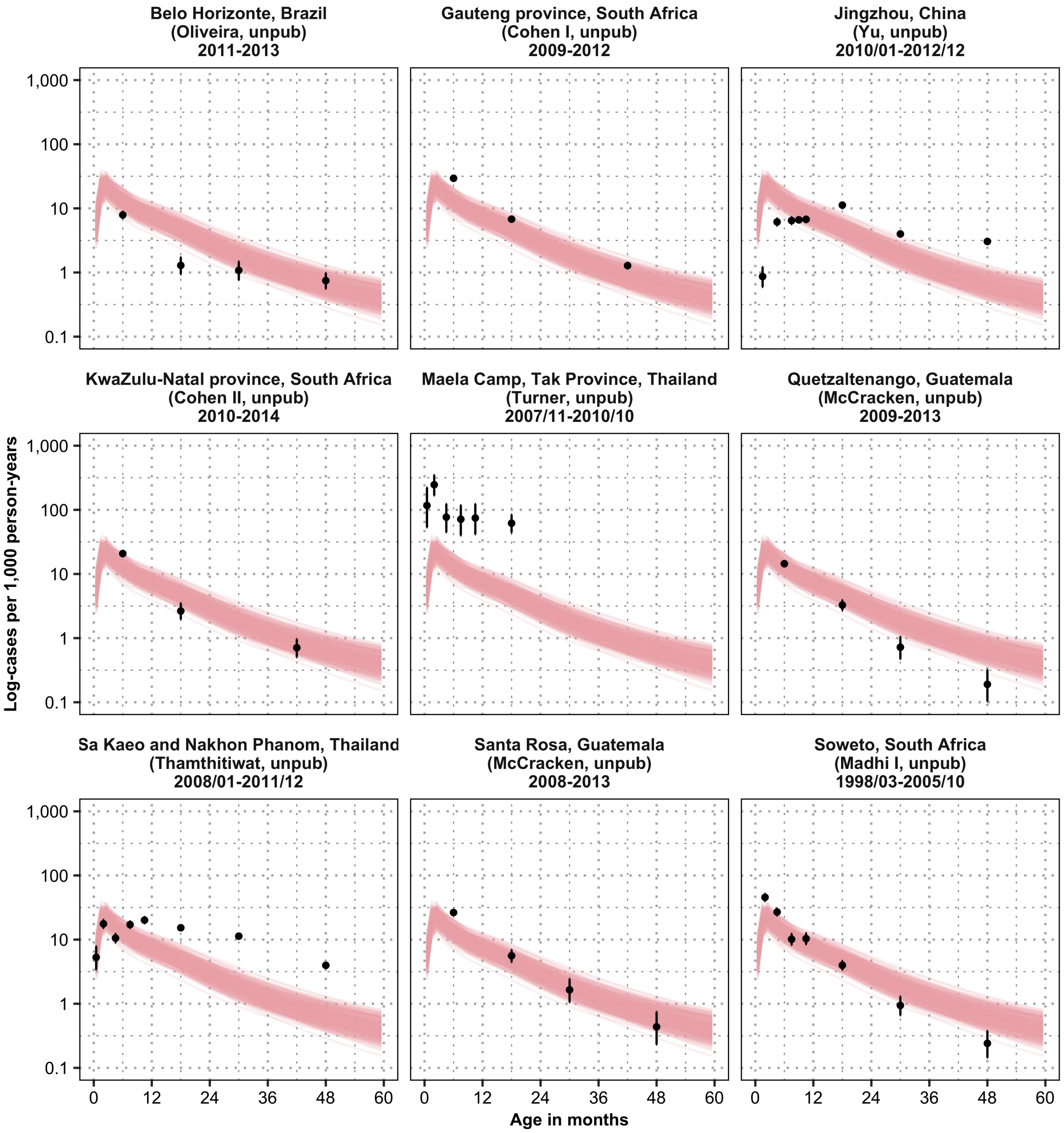

Figure G: Spline model predictions versus observed estimates of hospital-based incidence in UMIC settings. Each of the lines represents a random model prediction. The observed incidence estimated from each age group is placed at the midpoint of the age group, and the bars represent the 95% confidence interval of the incidence in that age group.

S2-1.3 Fit-vs-observed: Probability of hospitalization among cases in the community

↩ Return to the Table of Contents.

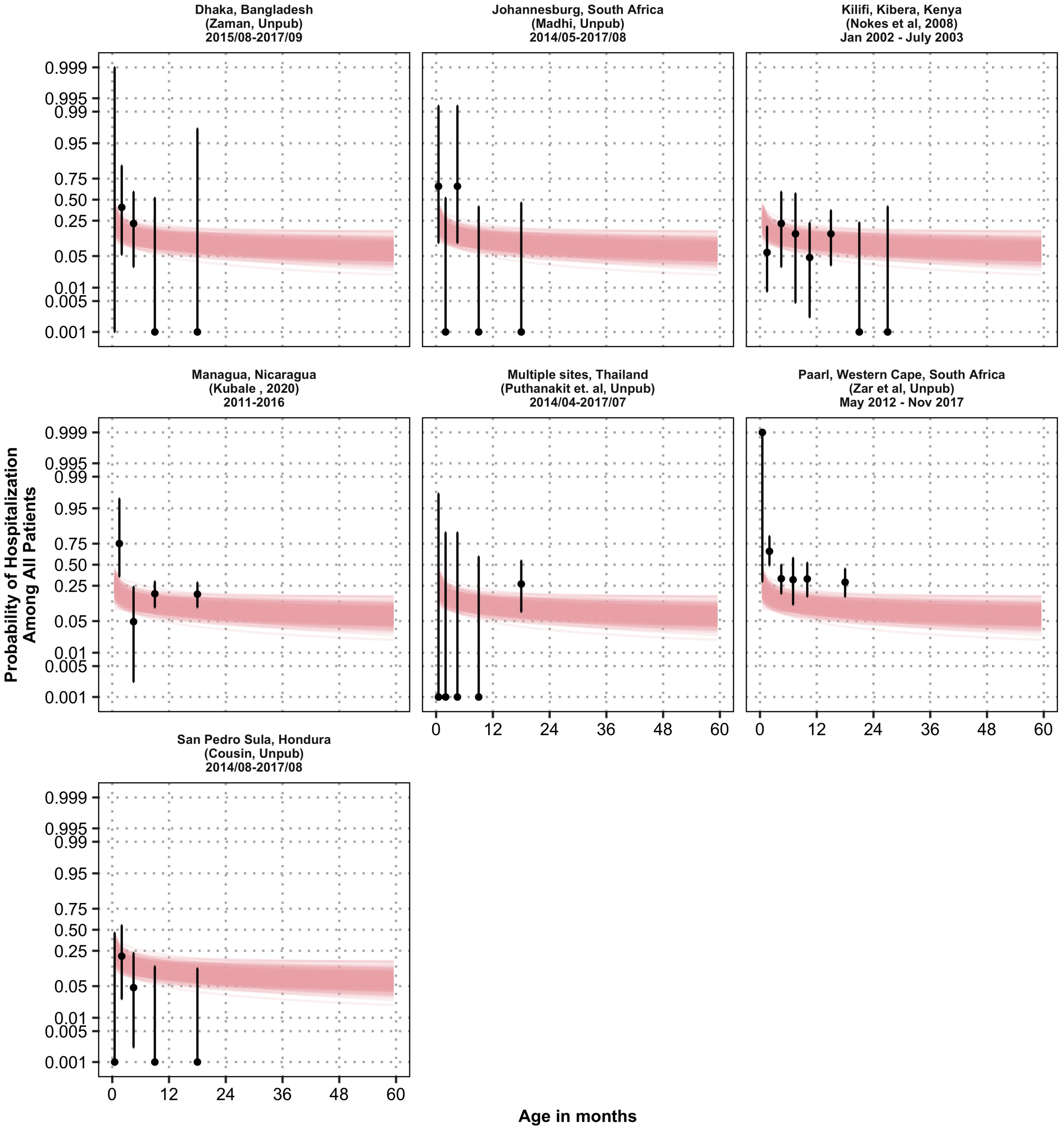

Figure H: Spline model predictions versus observed estimates of the probability of hospitalization among cases in the community in countries across all income groups. Each of the lines represents a random model prediction. The observed probability estimated from each age group is placed at the midpoint of the age group, and the bars represent the 95% confidence interval of the probability of hospitalization in that age group.

S2-1.4 Fit-vs-observed: Probability of death among hospitalized cases

↩ Return to the Table of Contents.

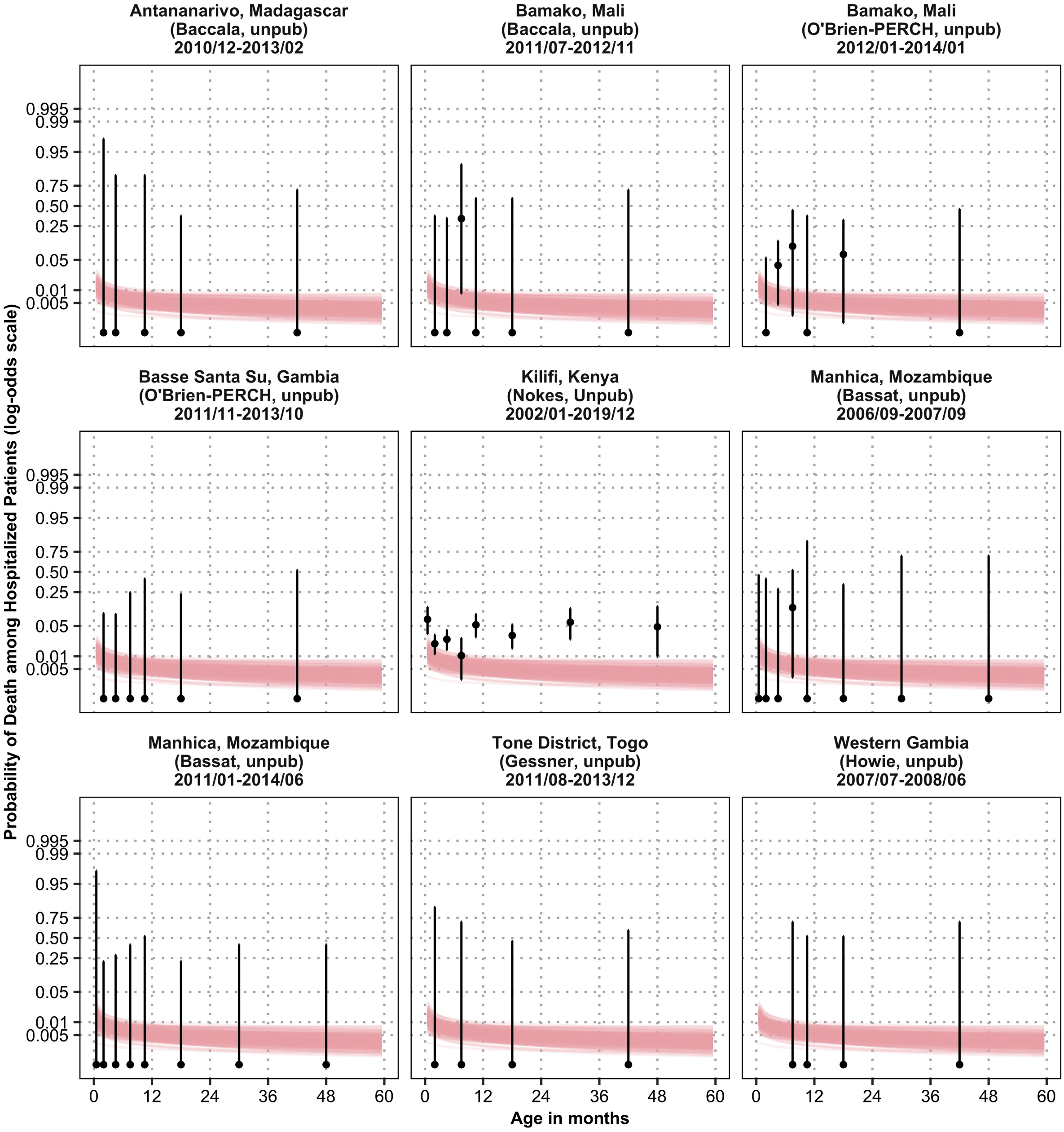

Figure I: Spline model predictions versus observed estimates of the probability of death among hospitalized cases in LIC settings. Each of the 1,000 lines represents a random model prediction. The observed probability estimated from each age group is placed at the midpoint of the age group, and the bars represent the 95% confidence interval of the probability of death among hospitalized cases of that age group.

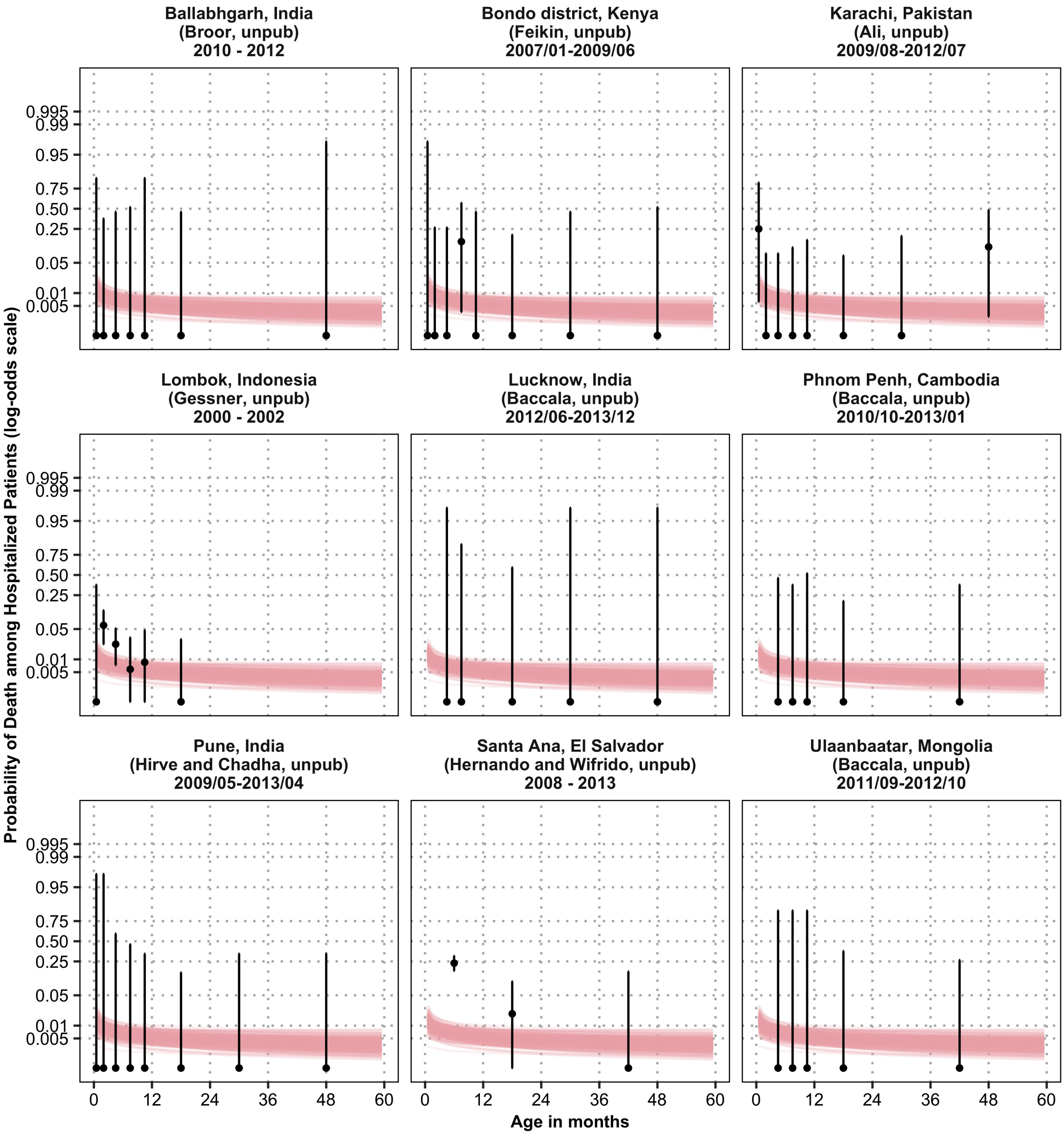

Figure J: Spline model predictions versus observed estimates of the probability of death among hospitalized cases in LMIC settings. Each of the 1,000 lines represents a random model prediction. The observed probability estimated from each age group is placed at the midpoint of the age group, and the bars represent the 95% confidence interval of the probability of death among hospitalized cases of that age group.

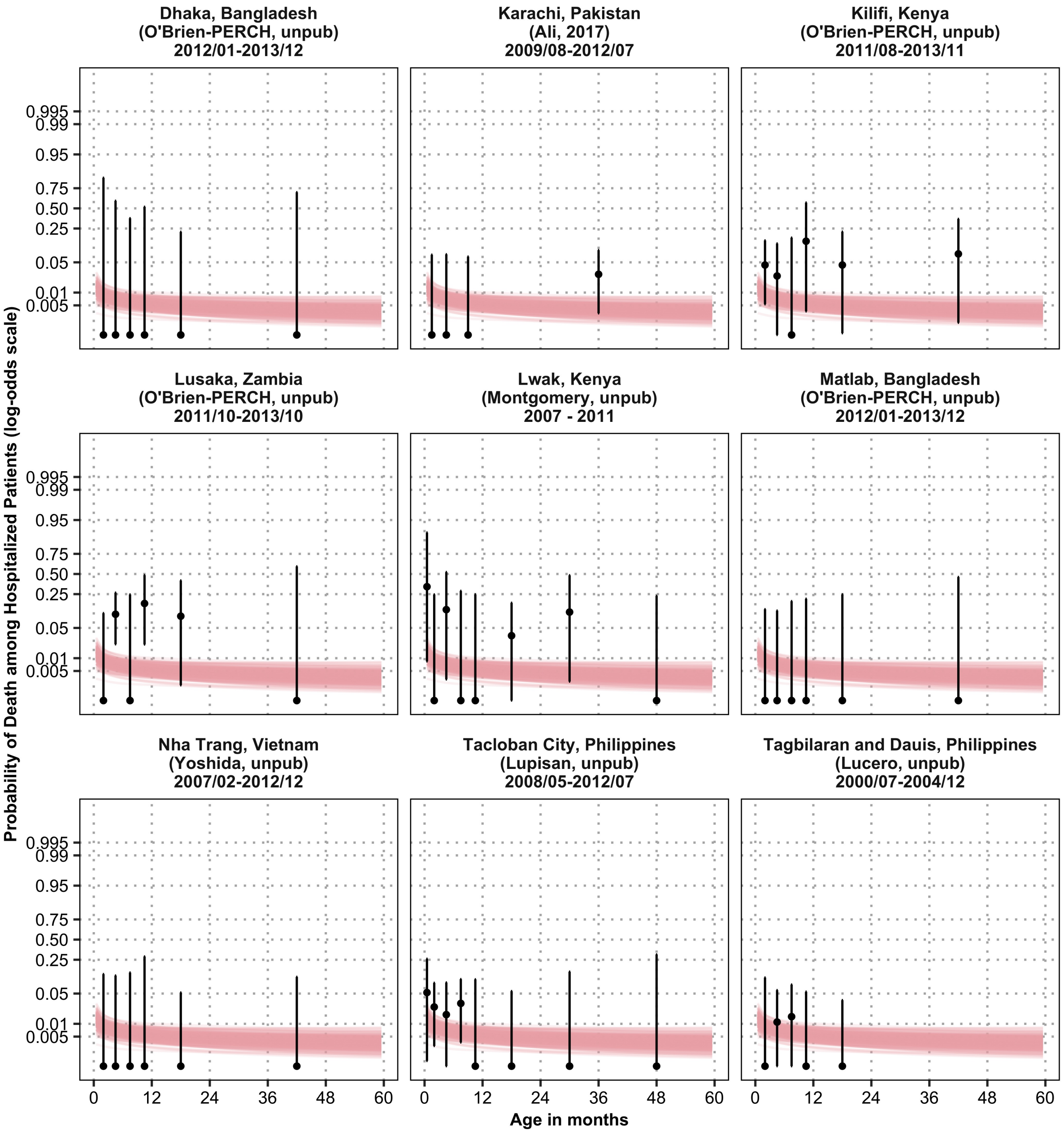

Figure K: Spline model predictions versus observed estimates of the probability of death among hospitalized cases in LMIC settings. Each of the 1,000 lines represents a random model prediction. The observed probability estimated from each age group is placed at the midpoint of the age group, and the bars represent the 95% confidence interval of the probability of death among hospitalized cases of that age group.

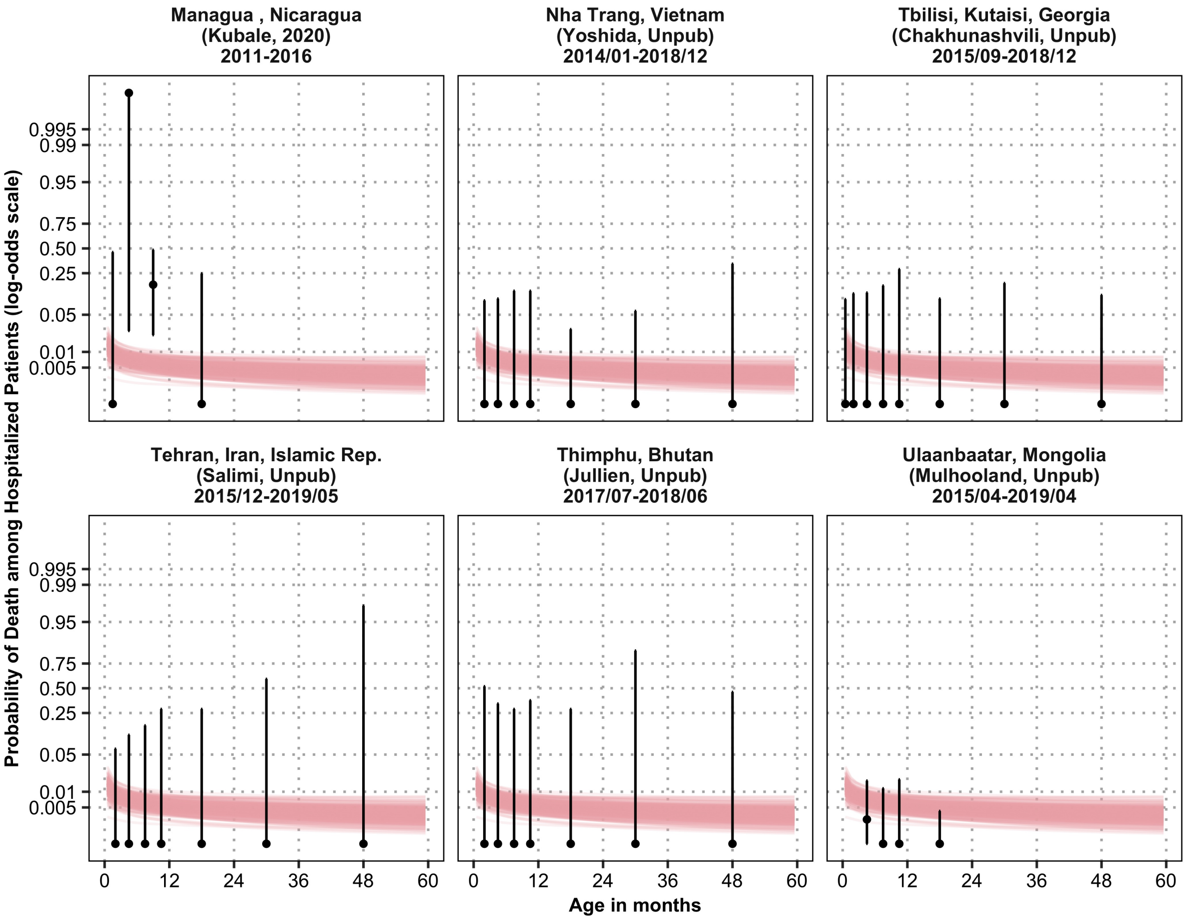

Figure L: Spline model predictions versus observed estimates of the probability of death among hospitalized cases in LMIC settings. Each of the 1,000 lines represents a random model prediction. The observed probability estimated from each age group is placed at the midpoint of the age group, and the bars represent the 95% confidence interval of the probability of death among hospitalized cases of that age group.

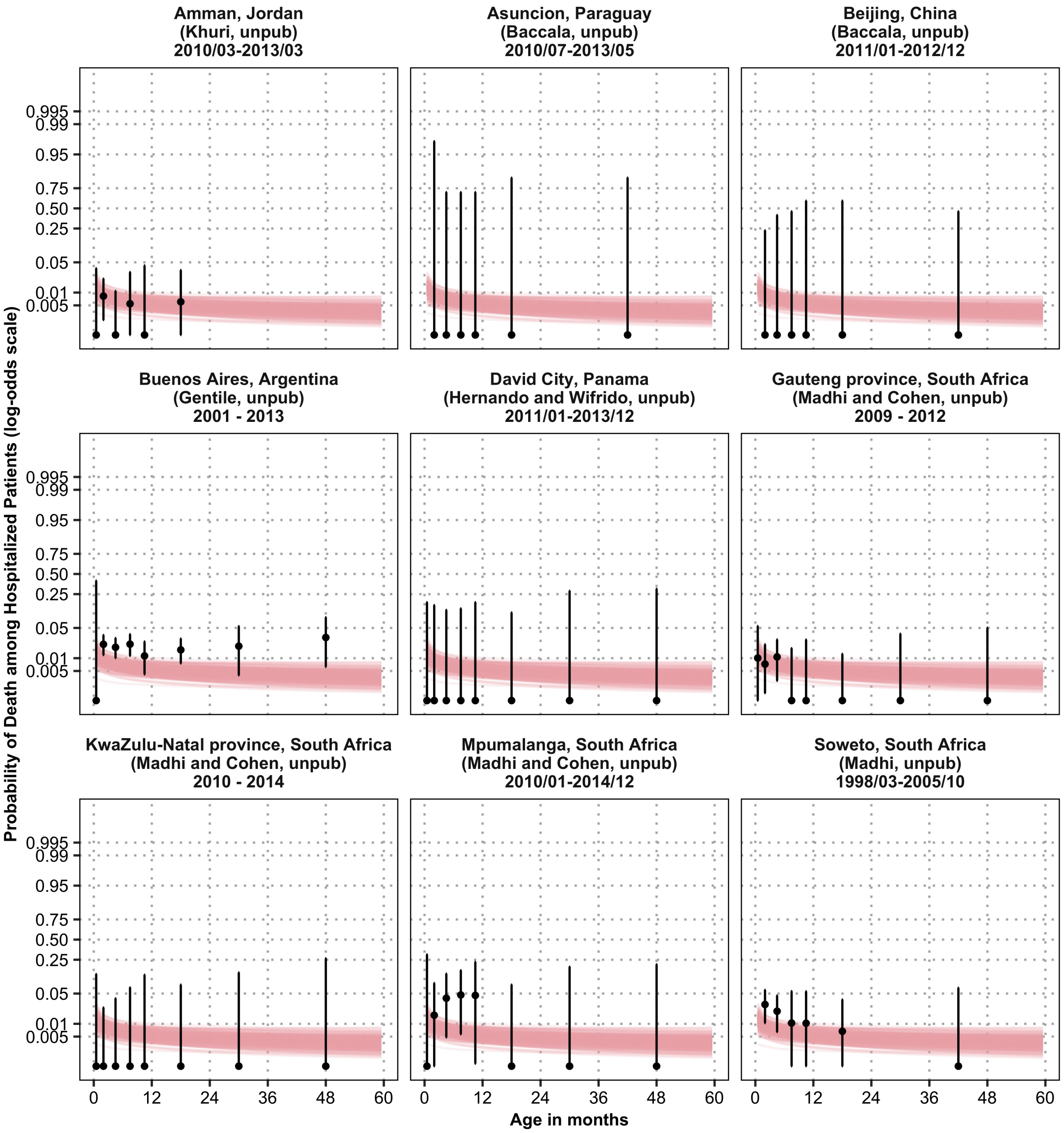

Figure M: Spline model predictions versus observed estimates of the probability of death among hospitalized cases in UMIC settings. Each of the 1,000 lines represents a random model prediction. The observed probability estimated from each age group is placed at the midpoint of the age group, and the bars represent the 95% confidence interval of the probability of death among hospitalized cases of that age group.

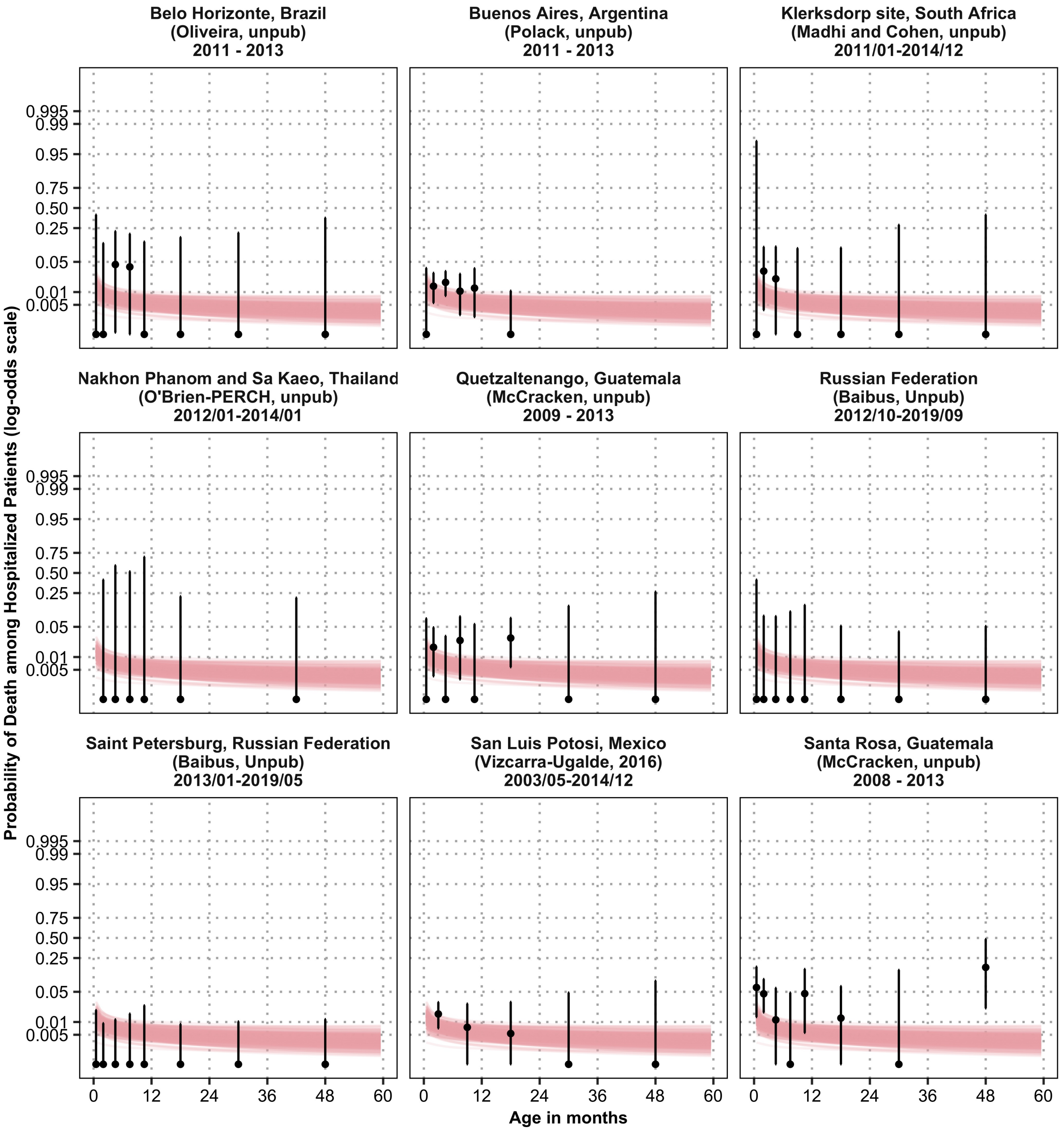

Figure N: Spline model predictions versus observed estimates of the probability of death among hospitalized cases in UMIC settings. Each of the 1,000 lines represents a random model prediction. The observed probability estimated from each age group is placed at the midpoint of the age group, and the bars represent the 95% confidence interval of the probability of death among hospitalized cases of that age group.

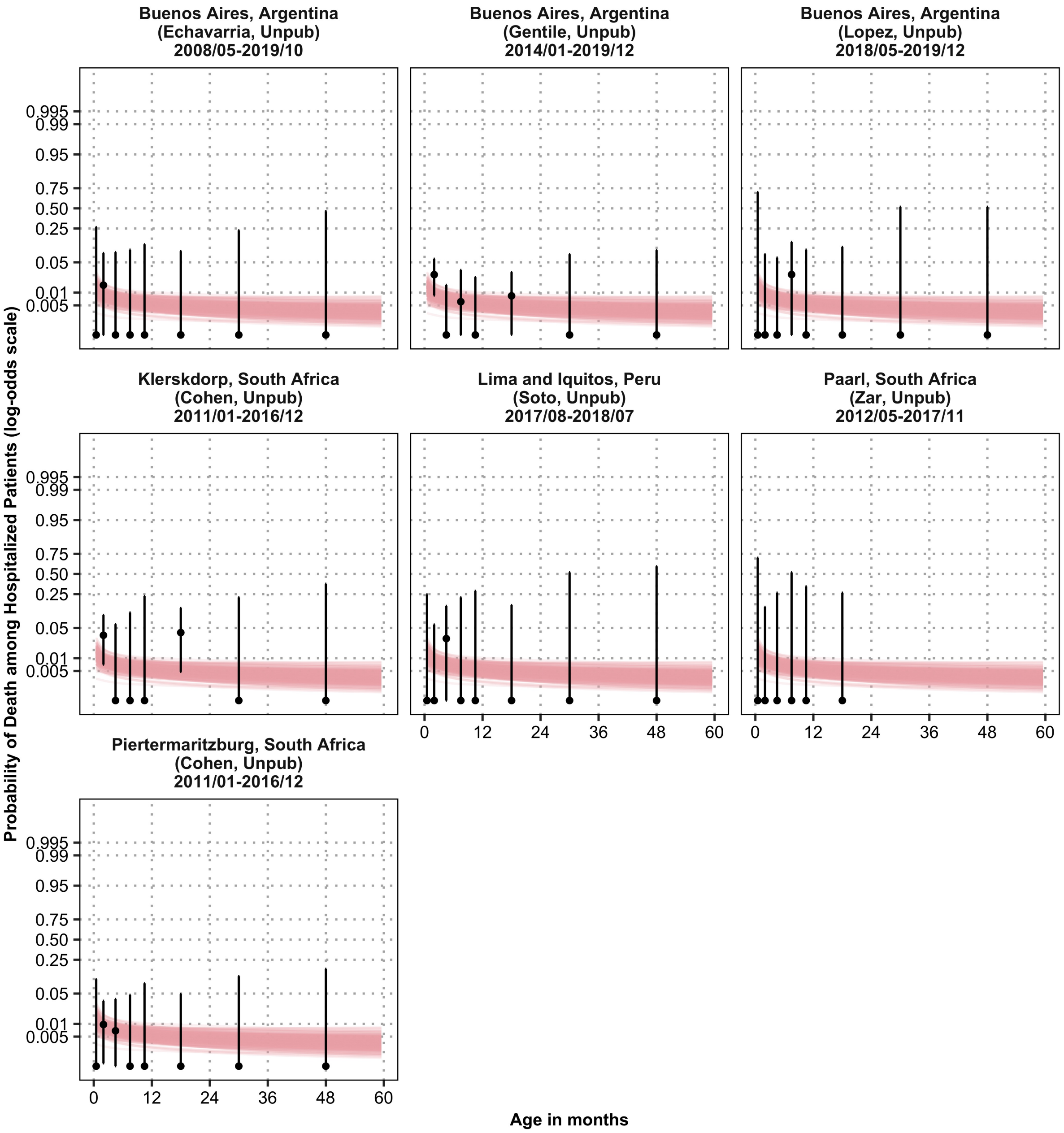

Figure O: Spline model predictions versus observed estimates of the probability of death among hospitalized cases in UMIC settings. Each of the 1,000 lines represents a random model prediction. The observed probability estimated from each age group is placed at the midpoint of the age group, and the bars represent the 95% confidence interval of the probability of death among hospitalized cases of that age group.

S2-2 Spline model predictions versus observations: out-of-sample validation

S2-2.1 Out-of-sample validation: Community-based incidence

↩ Return to the Table of Contents.

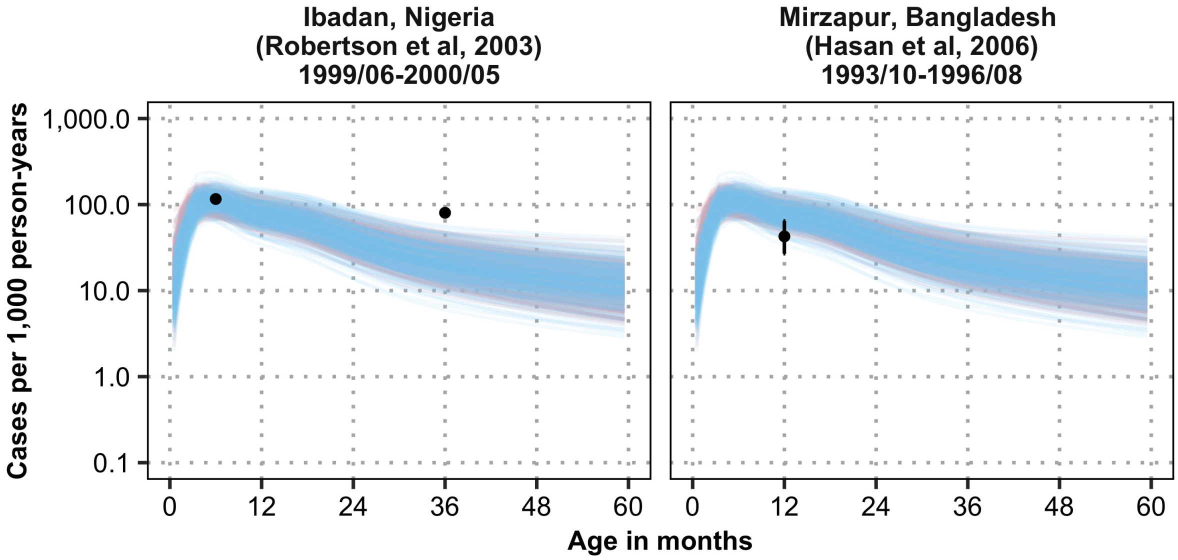

Figure P: Out-of-sample validation: Spline model predictions versus observed estimates of community-based incidence in settings that were not used to construct the incidence spline. Each of the lines represents a random model prediction. The pink lines represent predictions from the model estimated without a predictor for the country-level income group and the blue lines represent a model with the income group as a predictor. The observed incidence estimated from each age group is placed at the midpoint of the age group, and the bar represents the 95% confidence interval of the incidence in that age group. These studies were not used in constructing the incidence spline because the data were presented for two age groups only, which we judged could not adequately inform our estimates of the inflection points in the incidence trend.

S2-2.2 Out-of-sample validation: Hospital-based incidence

↩ Return to the Table of Contents.

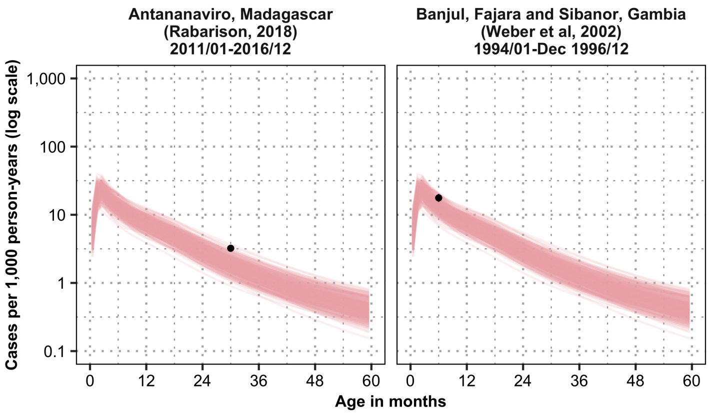

Figure Q: Out-of-sample validation: Spline model predictions versus observed estimates of hospital-based incidence in LIC settings that were not used to construct the incidence spline. Each of the lines represents a random model prediction. The observed incidence estimated from each age group is placed at the midpoint of the age group, and the bars represent the 95% confidence interval of the incidence in that age group. This study was not used in constructing the incidence spline because the data was presented for one age group only, which we judged could not adequately inform our estimates of the inflection points in the incidence trend.

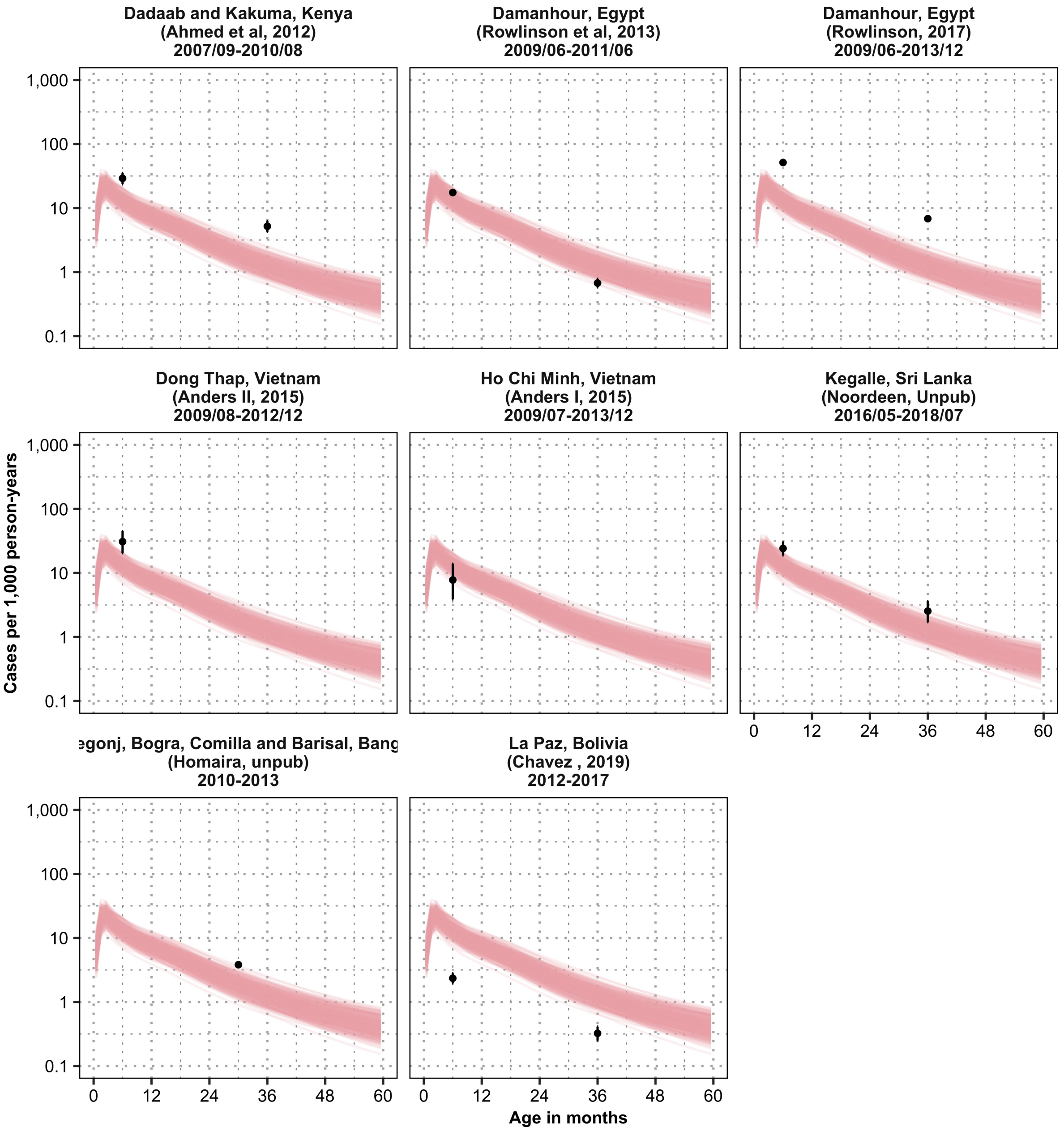

Figure R: Out-of-sample validation: Spline model predictions versus observed estimates of hospital-based incidence in LMIC settings that were not used to construct the incidence spline. Each of the 1,000 lines represents a random model prediction. The observed incidence estimated from each age group is placed at the midpoint of the age group, and the bars represent the 95% confidence interval of the incidence in that age group. These studies were not used in constructing the incidence spline because the data were presented for less than three age groups, which we judged could not adequately inform our estimates of the inflection points in the incidence trend.

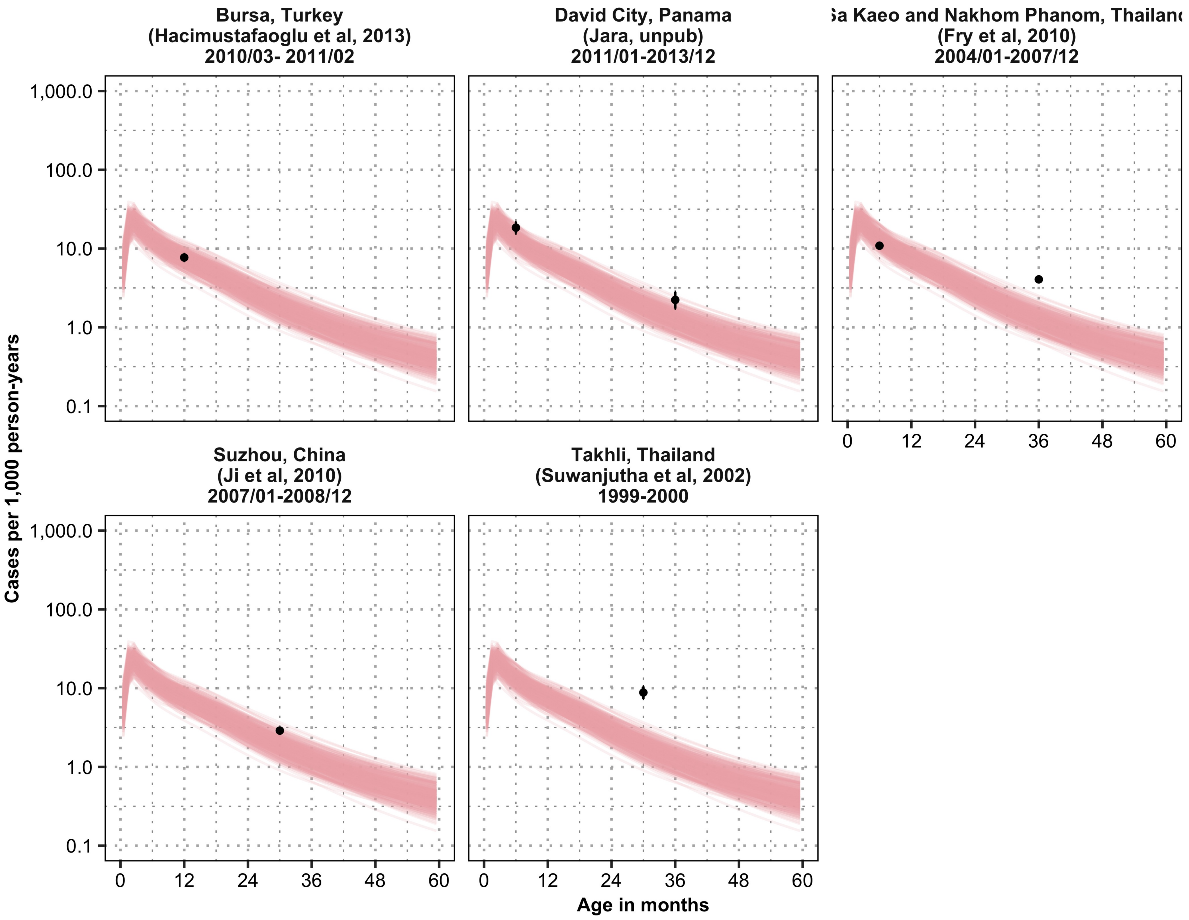

Figure S: Out-of-sample validation: Spline model predictions versus observed estimates of hospital-based incidence in UMIC settings that were not used to construct the incidence spline. Each of the 1,000 lines represents a random model prediction. The observed incidence estimated from each age group is placed at the midpoint of the age group, and the bars represent the 95% confidence interval of the incidence in that age group. These studies were not used in constructing the incidence spline because the data were presented for less than three age groups, which we judged could not adequately inform our estimates of the inflection points in the incidence trend.

S2-2.3 Out-of-sample validation: Probability of hospitalization among cases in the community

↩ Return to the Table of Contents.

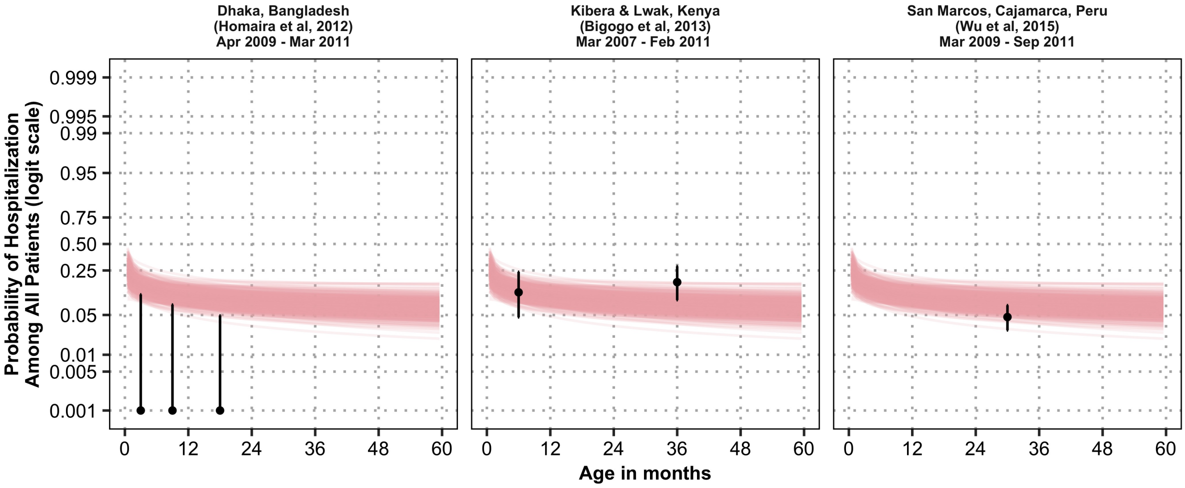

Figure T: Out-of-sample validation: Spline model predictions versus observed estimates of the probability of hospitalization among cases in the community in countries across all income groups from studies that were not used to construct the spline. Each of the 1,000 lines represents a random model prediction. The observed probability estimated from each age group is placed at the midpoint of the age group, and the bars represent the 95% confidence interval of the probability of hospitalization in that age group. These studies were not used in constructing the incidence spline because the data were presented for two age groups only, which we judged could not adequately inform our estimates of the inflection points in the trend across age groups.

S2-2.4 Out-of-sample validation: Probability of death among hospitalized cases

↩ Return to the Table of Contents.

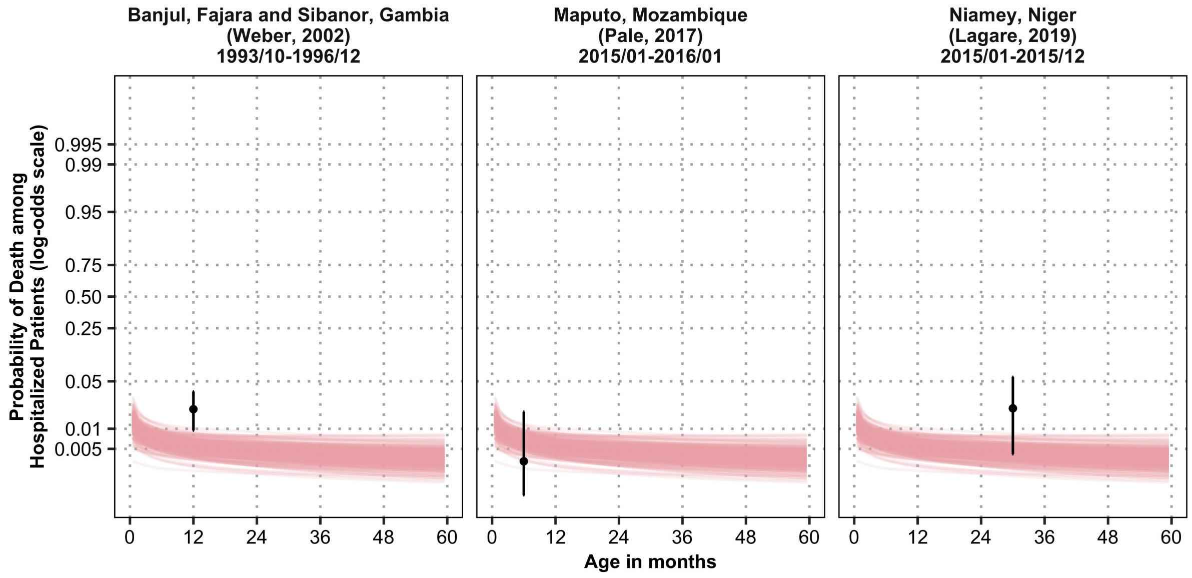

Figure U: Out-of-sample validation: Spline model predictions versus observed estimates of the probability of death among hospitalized cases in LIC settings that were not used to construct the probability spline. Each of the 1,000 lines represents a random model prediction. The observed probability estimated from each age group is placed at the midpoint of the age group, and the bars represent the 95% confidence interval of the probability of death among hospitalized cases of that age group. This study was not used in constructing the incidence spline because the data were presented for one age group only, which we judged could not adequately inform our estimates of the inflection points of the trend across age groups.

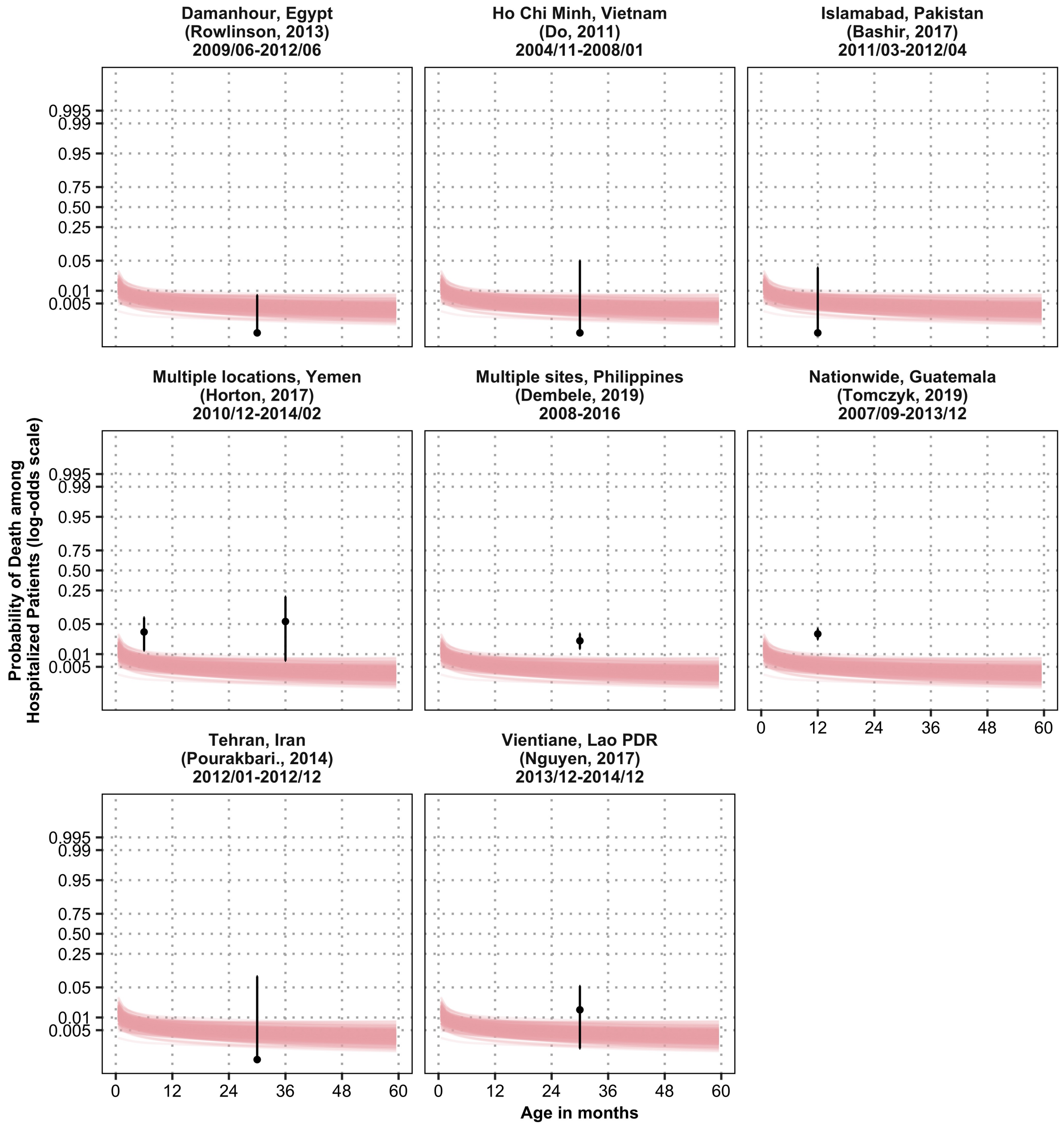

Figure V: Out-of-sample validation: Spline model predictions versus observed estimates of the probability of death among hospitalized cases in LMIC settings that were not used to construct the probability spline. Each of the 1,000 lines represents a random model prediction. The observed probability estimated from each age group is placed at the midpoint of the age group, and the bars represent the 95% confidence interval of the probability of death among hospitalized cases of that age group. These studies were not used in constructing the incidence spline because the data were presented for one age group only, which we judged could not adequately inform our estimates of the inflection points of the trend across age groups.

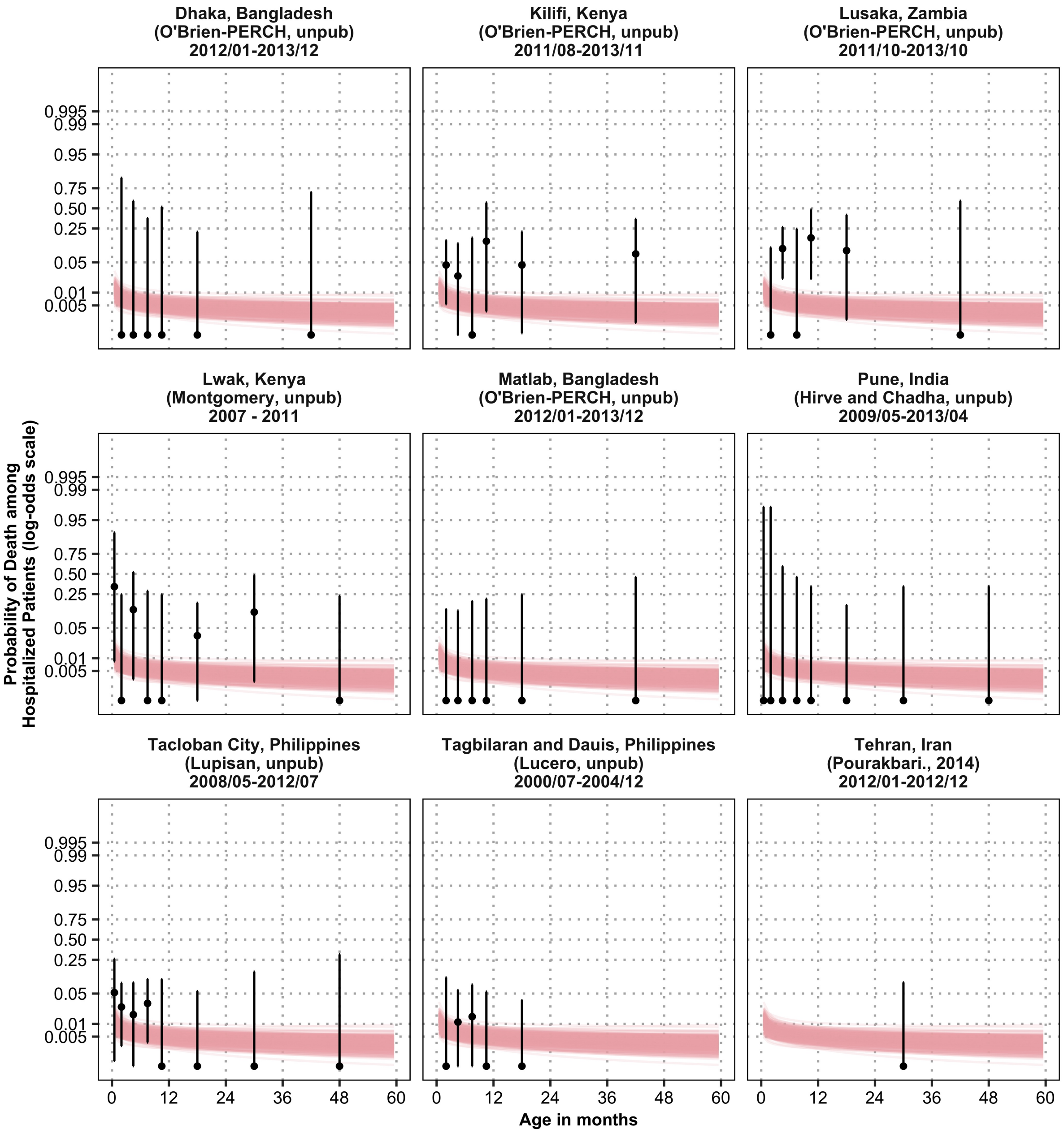

Figure W: Out-of-sample validation: Spline model predictions versus observed estimates of the probability of death among hospitalized cases in LMIC settings that were not used to construct the probability spline. Each of the 1,000 lines represents a random model prediction. The observed probability estimated from each age group is placed at the midpoint of the age group, and the bars represent the 95% confidence interval of the probability of death among hospitalized cases of that age group. These studies were not used in constructing the incidence spline because the data were presented for one age group only, which we judged could not adequately inform our estimates of the inflection points of the trend across age groups.

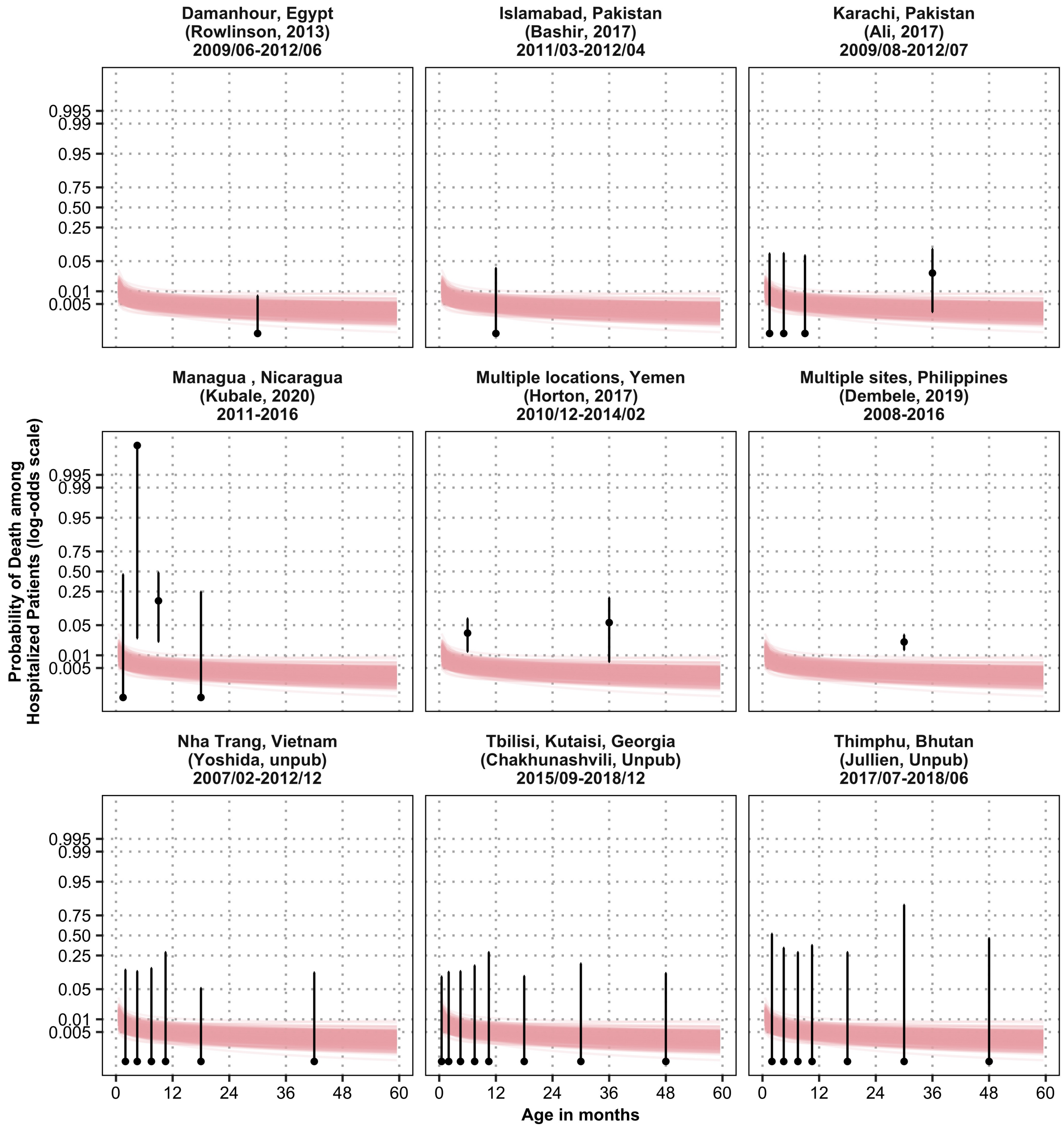

Figure X: Out-of-sample validation: Spline model predictions versus observed estimates of the probability of death among hospitalized cases in LMIC settings that were not used to construct the probability spline. Each of the 1,000 lines represents a random model prediction. The observed probability estimated from each age group is placed at the midpoint of the age group, and the bars represent the 95% confidence interval of the probability of death among hospitalized cases of that age group. These studies were not used in constructing the incidence spline because the data were presented for one age group only, which we judged could not adequately inform our estimates of the inflection points of the trend across age groups.

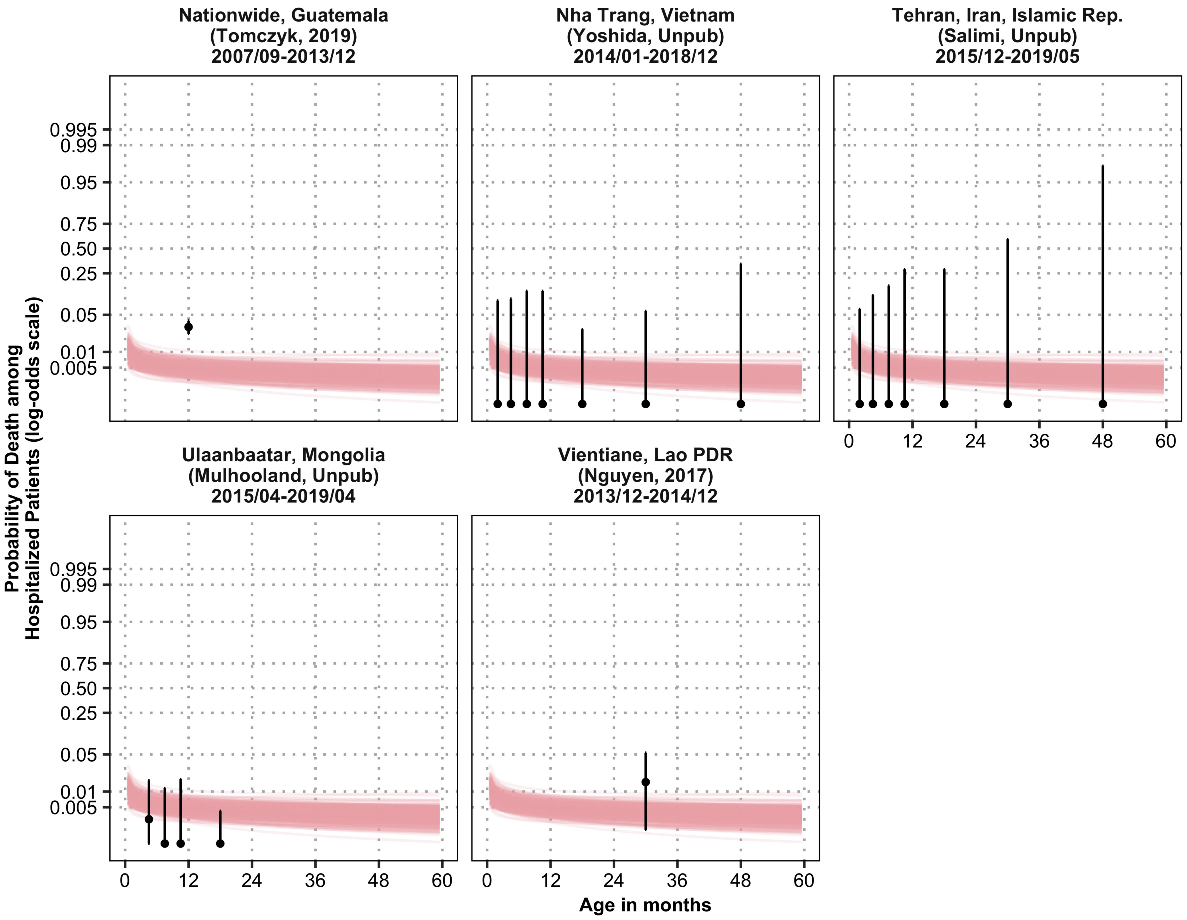

Figure Y: Out-of-sample validation: Spline model predictions versus observed estimates of the probability of death among hospitalized cases in LMIC settings that were not used to construct the probability spline. Each of the 1,000 lines represents a random model prediction. The observed probability estimated from each age group is placed at the midpoint of the age group, and the bars represent the 95% confidence interval of the probability of death among hospitalized cases of that age group. These studies were not used in constructing the incidence spline because the data were presented for one age group only, which we judged could not adequately inform our estimates of the inflection points of the trend across age groups.

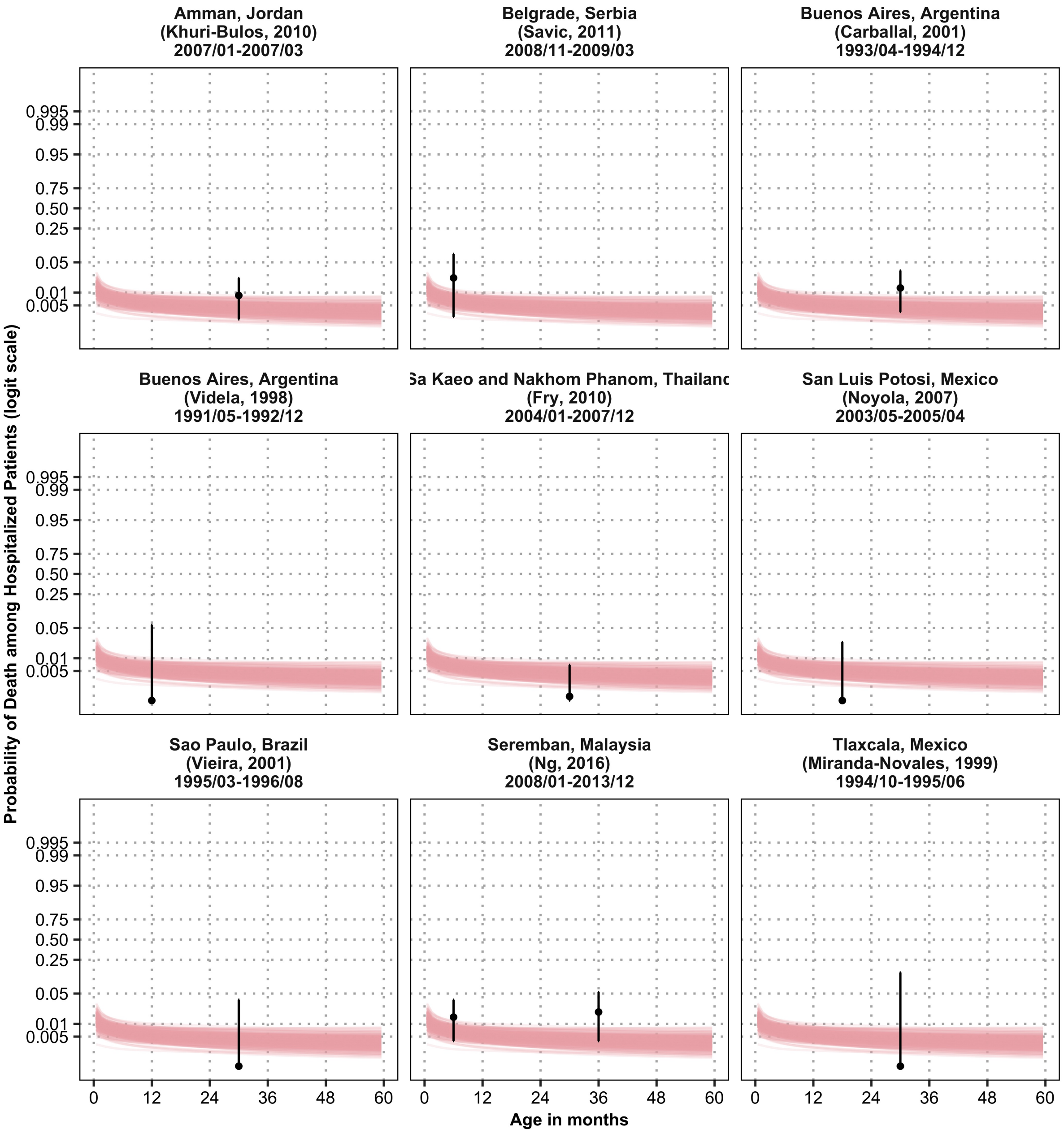

Figure Z: Out-of-sample validation: Spline model predictions versus observed estimates of the probability of death among hospitalized cases in UMIC settings that were not used to construct the probability spline. Each of the 1,000 lines represents a random model prediction. The observed probability estimated from each age group is placed at the midpoint of the age group, and the bars represent the 95% confidence interval of the probability of death among hospitalized cases of that age group. These studies were not used in constructing the incidence spline because the data were presented for one age group only, which we judged could not adequately inform our estimates of the inflection points of the trend across age groups.

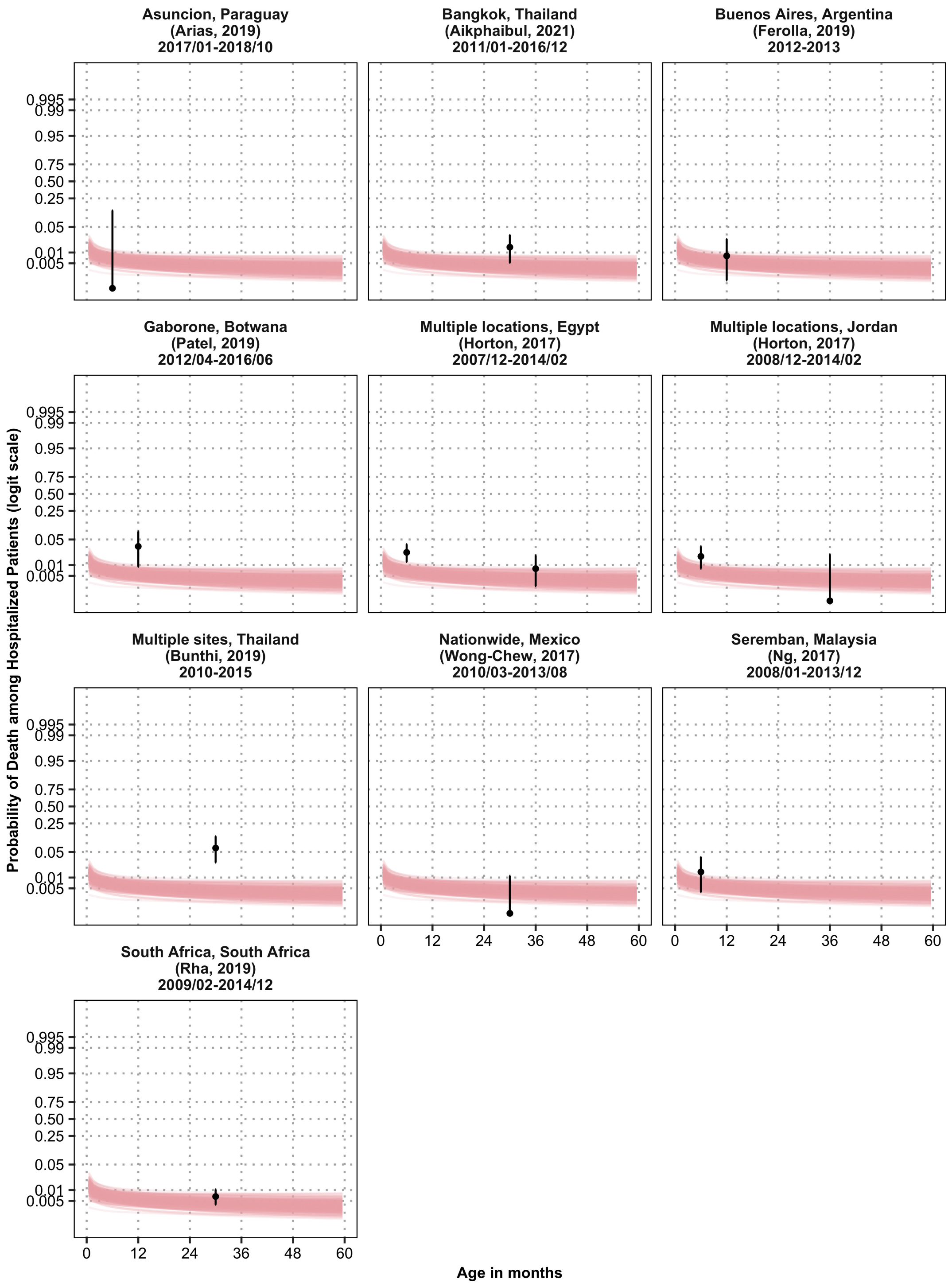

Figure AA: Out-of-sample validation: Spline model predictions versus observed estimates of the probability of death among hospitalized cases in UMIC settings that were not used to construct the probability spline. Each of the 1,000 lines represents a random model prediction. The observed probability estimated from each age group is placed at the midpoint of the age group, and the bars represent the 95% confidence interval of the probability of death among hospitalized cases of that age group. These studies were not used in constructing the incidence spline because the data were presented for one age group only, which we judged could not adequately inform our estimates of the inflection points of the trend across age groups.

S2-3 Spline model predictions versus observations: within-sample validation for severity outcomes

S2-3.1 Fit-vs-observed: Probability of severe cases among community-based incidence studies

↩ Return to the Table of Contents.

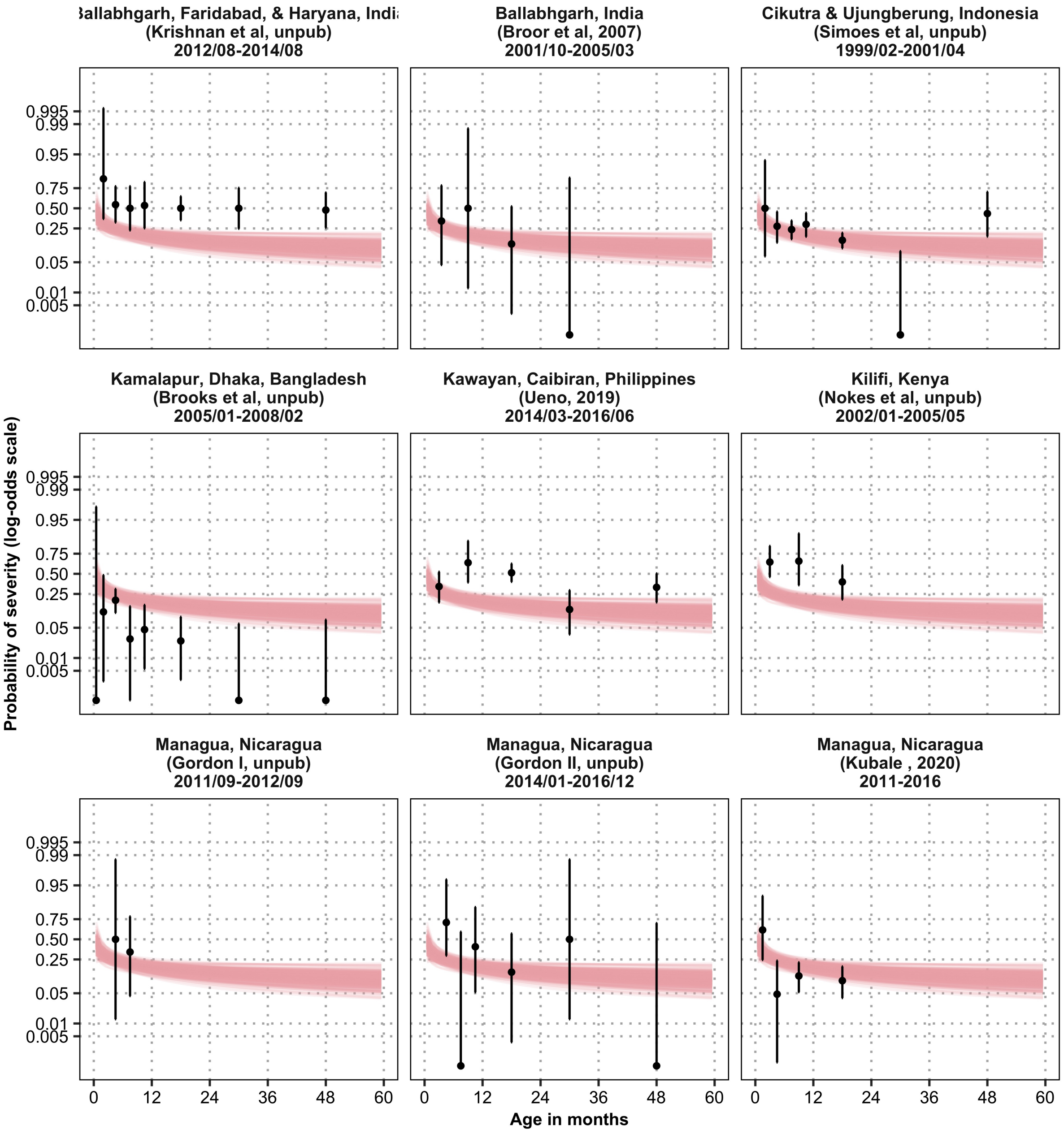

Figure AB: Spline model predictions versus observed estimates of the probability of severity among cases in community-based studies. Each of the lines represents a random model prediction. The observed probability estimated from each age group is placed at the midpoint of the age group, and the bars represent the 95% confidence interval of the probability in that age group.

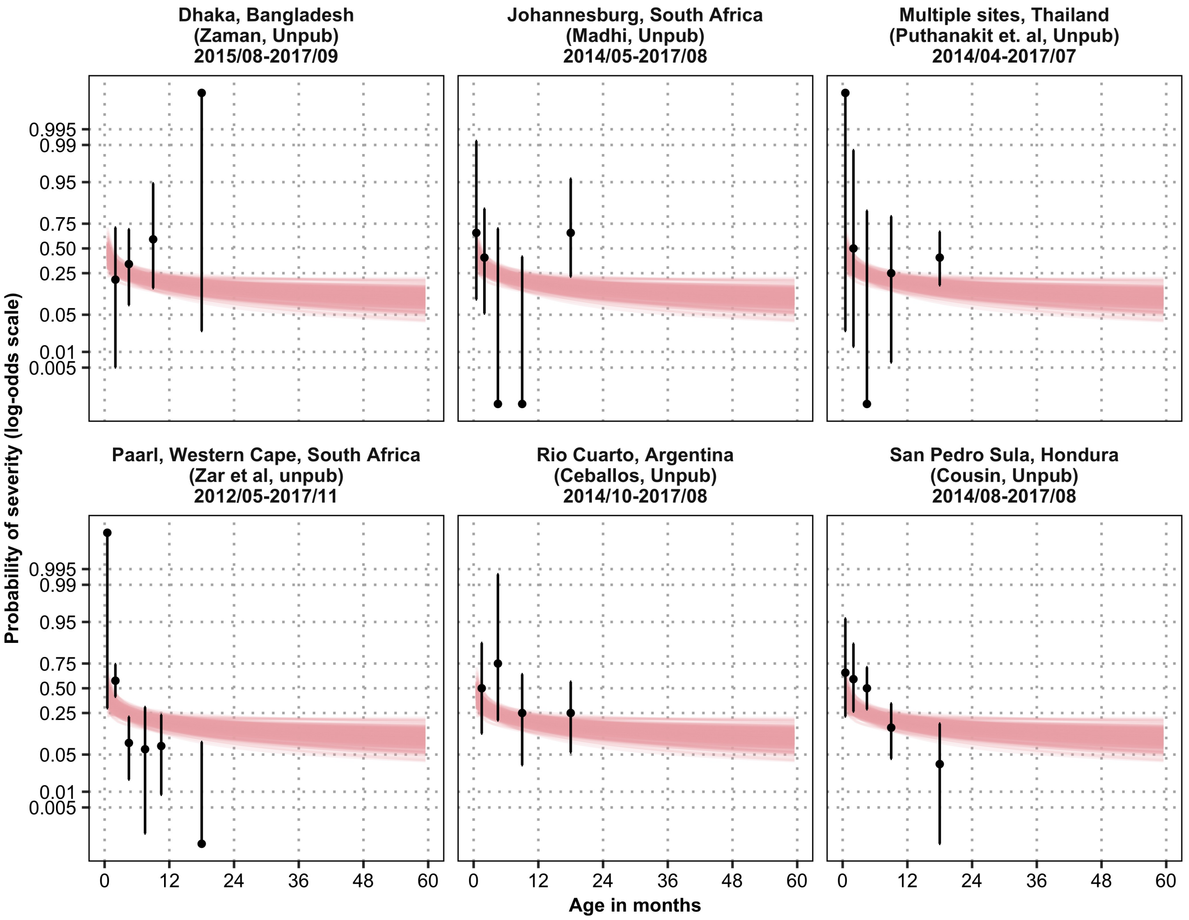

Figure AC: Spline model predictions versus observed estimates of the probability of severity among cases in community-based studies. Each of the lines represents a random model prediction. The observed probability estimated from each age group is placed at the midpoint of the age group, and the bars represent the 95% confidence interval of the probability in that age group.

S2-3.2 Fit-vs-observed: Probability of very severe cases among community-based incidence studies

↩ Return to the Table of Contents.

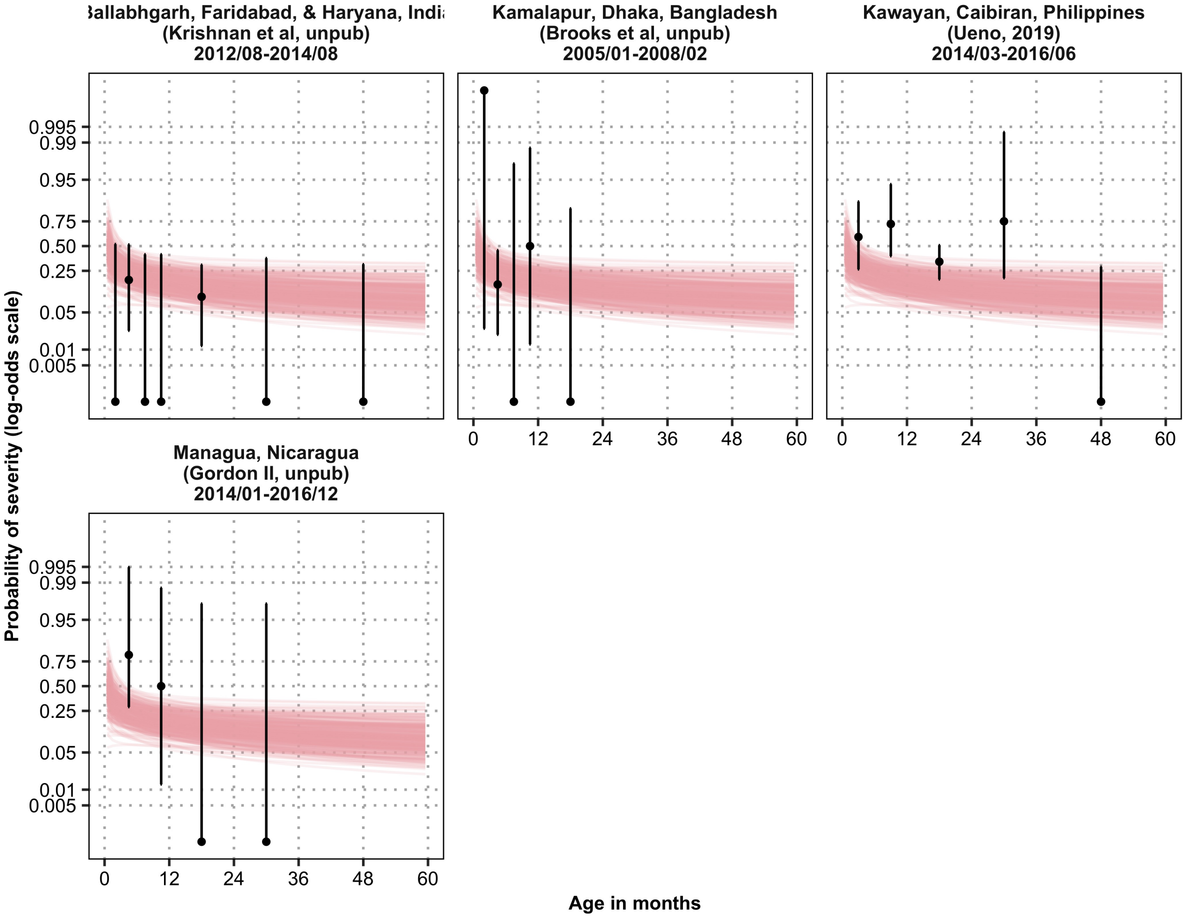

Figure AD: Spline model predictions versus observed estimates of the probability of very severe cases among cases in community-based studies. Each of the lines represents a random model prediction. The observed probability estimated from each age group is placed at the midpoint of the age group, and the bars represent the 95% confidence interval of the probability in that age group.

S2-3.3 Fit-vs-observed: Probability of severe cases among hospital-based incidence studies

↩ Return to the Table of Contents.

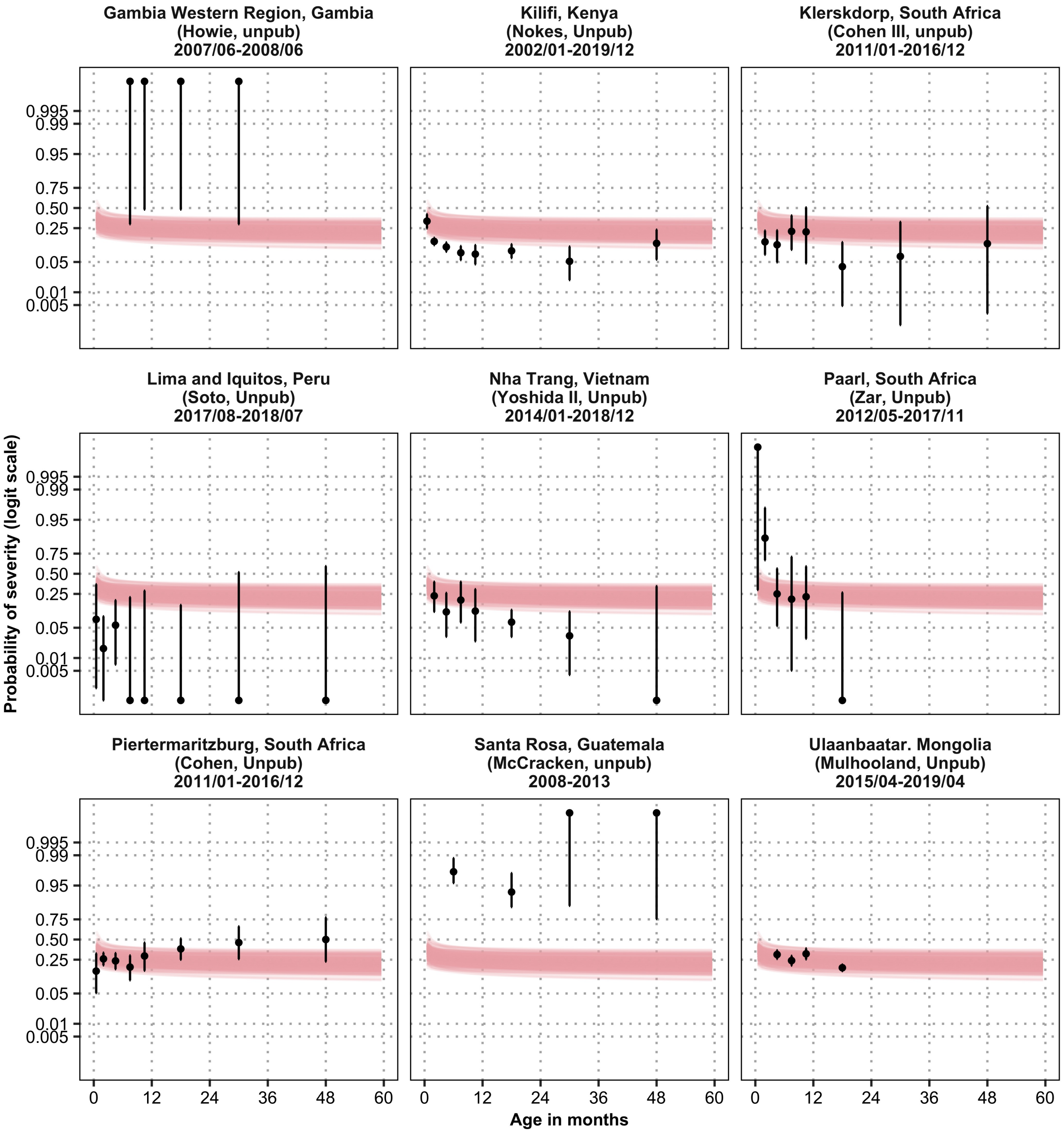

Figure AE: Spline model predictions versus observed estimates of the probability of severity among cases in hospital-based studies. Each of the lines represents a random model prediction. The observed probability estimated from each age group is placed at the midpoint of the age group, and the bars represent the 95% confidence interval of the probability in that age group.

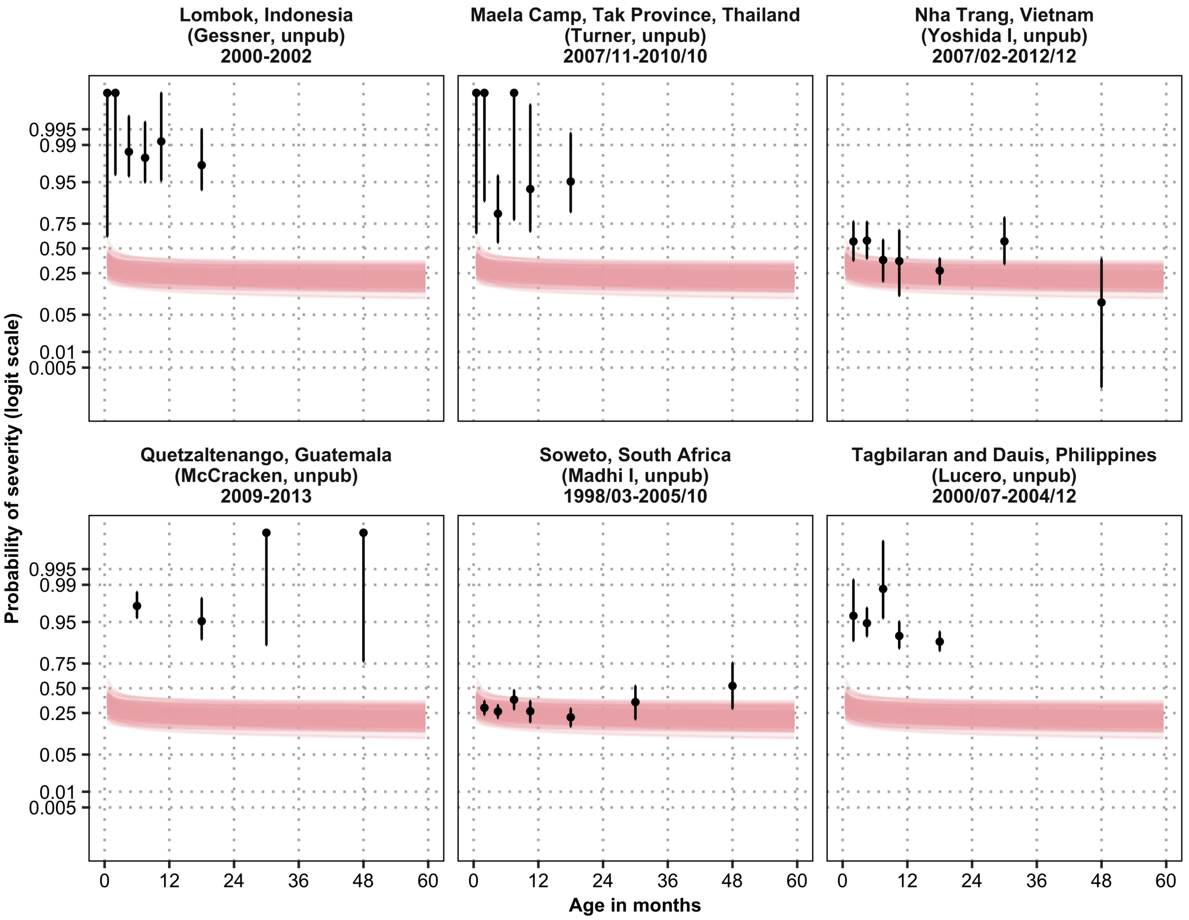

Figure AF: Spline model predictions versus observed estimates of the probability of severity among cases in hospital-based studies. Each of the lines represents a random model prediction. The observed probability estimated from each age group is placed at the midpoint of the age group, and the bars represent the 95% confidence interval of the probability in that age group.

S2-3.4 Fit-vs-observed: Probability of very severe cases among hospital-based incidence studies

↩ Return to the Table of Contents.

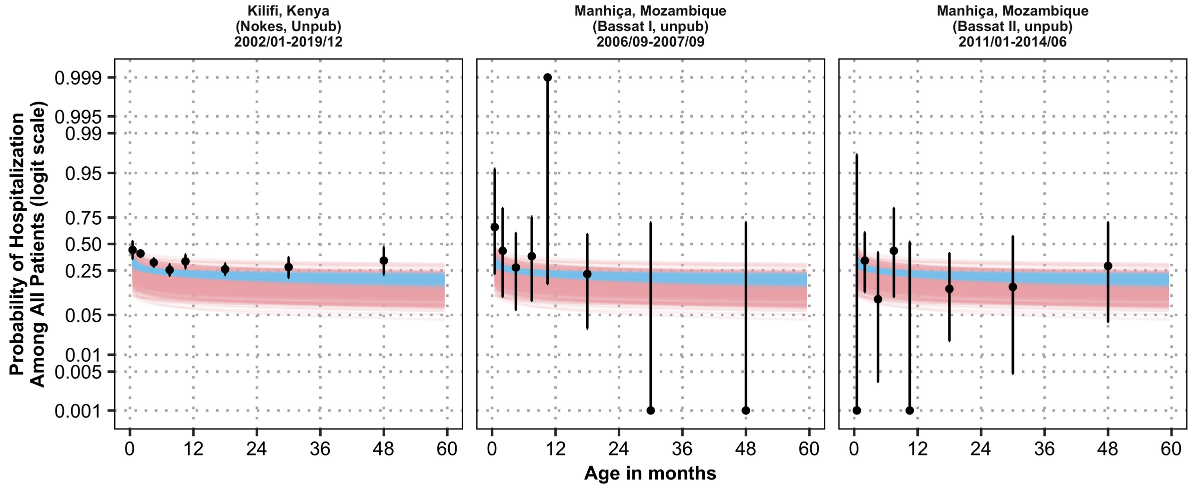

Figure AG: Spline model predictions versus observed estimates of the probability of very severe cases among cases in hospital-based studies in LICs. Each of the lines represents a random model prediction. The observed probability estimated from each age group is placed at the midpoint of the age group, and the bars represent the 95% confidence interval of the probability in that age group.

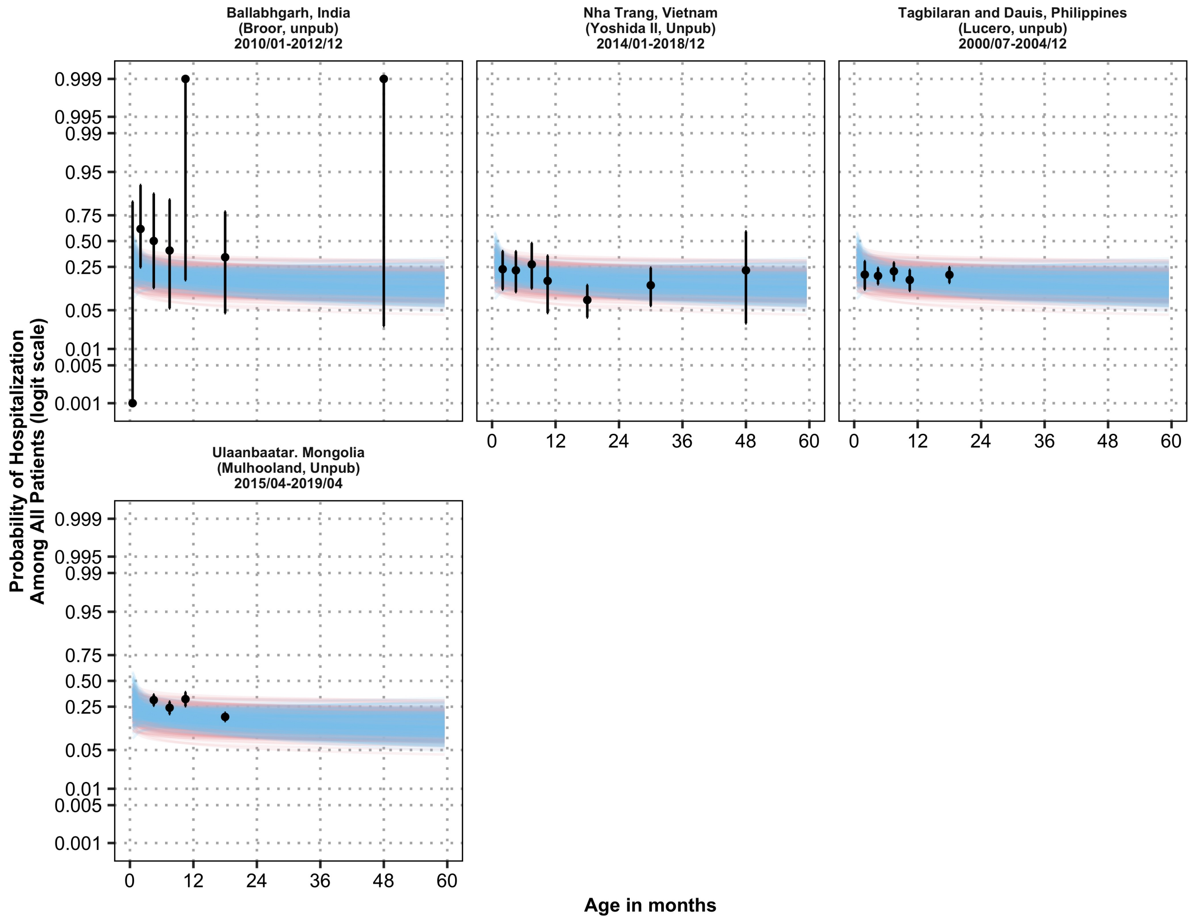

Figure AH: Spline model predictions versus observed estimates of the probability of very severe cases among cases in hospital-based studies in LMICs. Each of the lines represents a random model prediction. The observed probability estimated from each age group is placed at the midpoint of the age group, and the bars represent the 95% confidence interval of the probability in that age group.

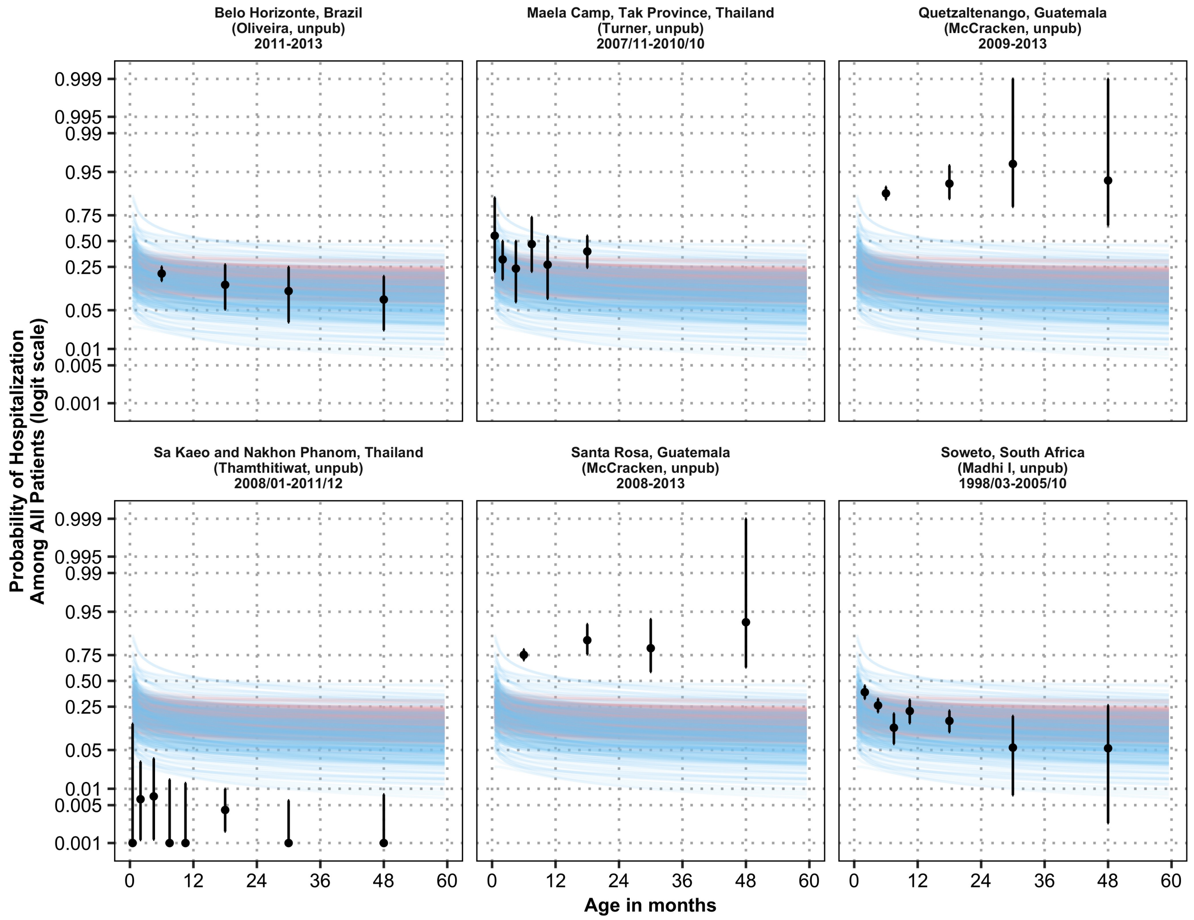

Figure AI: Spline model predictions versus observed estimates of the probability of very severe cases among cases in hospital-based studies in UMICs. Each of the lines represents a random model prediction. The observed probability estimated from each age group is placed at the midpoint of the age group, and the bars represent the 95% confidence interval of the probability in that age group.
