# Supplementary material for: The age profile of respiratory syncytial virus burden in preschool children of low- and middle-income countries: A semi-parametric, meta-regression approach
Source: PLoS Med. 2023 Jul 17;20(7):e1004250. doi: 10.1371/journal.pmed.1004250 (PMC10389726; doi:10.1371/journal.pmed.1004250)
Supplement: S3 Text — Fig A. Splines of the probability of severe and very severe disease among community-based and hospital-based cases. Fig B. Splines of the probability of severe and very severe disease among community-based and hospital-based cases. Fig C. RSV cases, hospitalizations of severe and very severe disease per 1,000 person-years according to Spline Models (SM) I and II. Table A. Model selection via the generalized likelihood ratio test for severe and very severe disease. Fig D. Mean, median, and peak age of each severe and very severe disease among community-based and hospital-based cases. Fig E. Proportions of each outcome that fall under key age brackets for severe and very severe case burden. Fig F. Sensitivity analysis of the burden of RSV cases, hospitalizations, and deaths by age in low-income countries (LICs). Fig G. Sensitivity analysis of the burden of RSV cases, hospitalizations, and deaths by age in low-income countries (LMICs). Fig H. Sensitivity analysis of the burden of RSV cases, hospitalizations, and deaths by age in low-income countries (UMICs). (PDF) [file pmed.1004250.s003.pdf]

Supporting Information

The age profile of respiratory syncytial virus burden in pre-school children of low- and middle-income countries: A semi-parametric, meta-regression approach

S3. Supplementary Results: Additional Projections

Contents

S3.1 Percent of cases, hospitalizations, and deaths under 5 in each age group - with uncertainty . . S3.2
S3.2 Severe and very severe RSV disease . . . S3.3
S3.3 Additional results of burden models (BMs) by income group and by age . . . S3.7
S3.4 Deaths in the community and total deaths . . . S3.10

List of Tables

A Model selection via the generalized likelihood ratio test. FE: fixed-effects. RE: random-effects. DF: degrees of freedom. . . . S3.3
B All deaths: deaths in the community (which did not reach a health facility), in-hospital deaths, and total deaths. . . . S3.10

List of Figures

A Splines of the probability of severe and very severe disease among community-based and hospital-based cases . . . S3.2
B Splines of the probability of severe and very severe disease among community-based and hospital-based cases . . . S3.3
C RSV cases, hospitalizations of severe and very severe disease per 1,000 person-years according to Spline Models (SM) I and II. . . . S3.4
D Mean, median, and the peak age of each severe and very severe disease among community-based and hospital-based cases . . . S3.5
E Proportions of each outcome that fall under key age brackets for severe and very severe case burden . . . S3.6
F Sensitivity analysis of the burden of RSV cases, hospitalizations, and deaths by age in low-income countries (LICs) . . . S3.7
G Sensitivity analysis of the burden of RSV cases, hospitalizations, and deaths by age in lower-middle-income countries (LMICs) . . . S3.8
H Sensitivity analysis of the burden of RSV cases, hospitalizations, and deaths by age in upper-middle-income countries (UMICs) . . . S3.9

S3.1 Percent of cases, hospitalizations, and deaths under 5 in each age group - with uncertainty

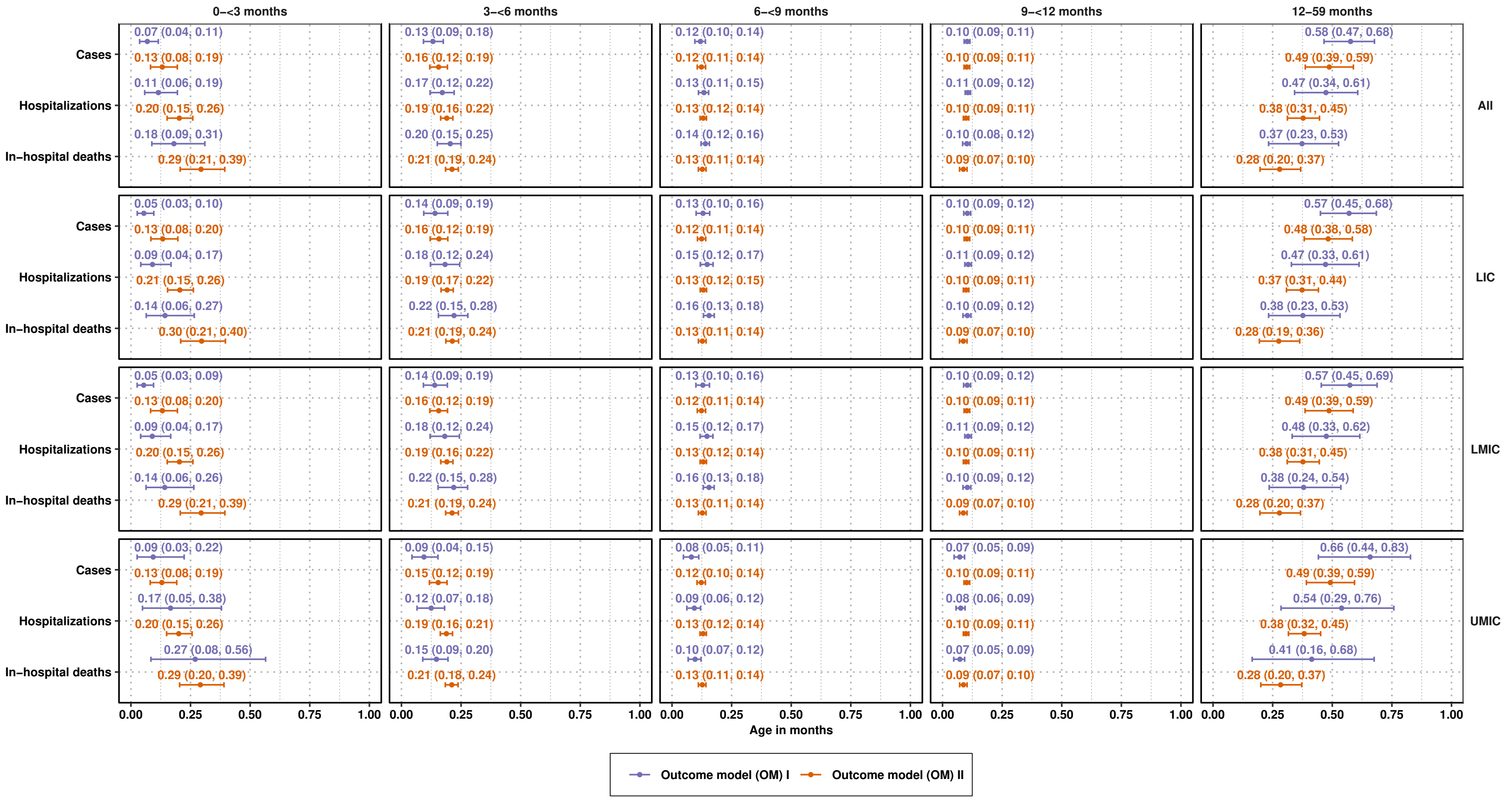

Figure A: Proportions of each outcome that fall under key age brackets, with 95% confidence intervals. Abbreviations: LIC: low-income countries, LMIC: lower-middle-income countries, UMIC: upper-middle-income countries.

S3.2 Severe and very severe RSV disease

↩ Return to the Table of Contents.

	Income Group FE	Income Group FE and RE
Spline I.S: Probability of severity among cases in the community	P=1 (DF=2, Chi2=0)	P=0.97 (DF=3, Chi2=0.24)
Spline II.S: Probability of chest indrawing among hospitalizations	P=0.58 (DF=4, Chi2=2.86)	P=0.99 (DF=7, Chi2=1.09)
Spline II.VS: Probability of extreme severity among all hospitalizations	P=1 (DF=4, Chi2=0)	P=<0.01 (DF=7, Chi2=22.6)

Table A: Model selection via the generalized likelihood ratio test. FE: fixed-effects. RE: random-effects. DF: degrees of freedom.

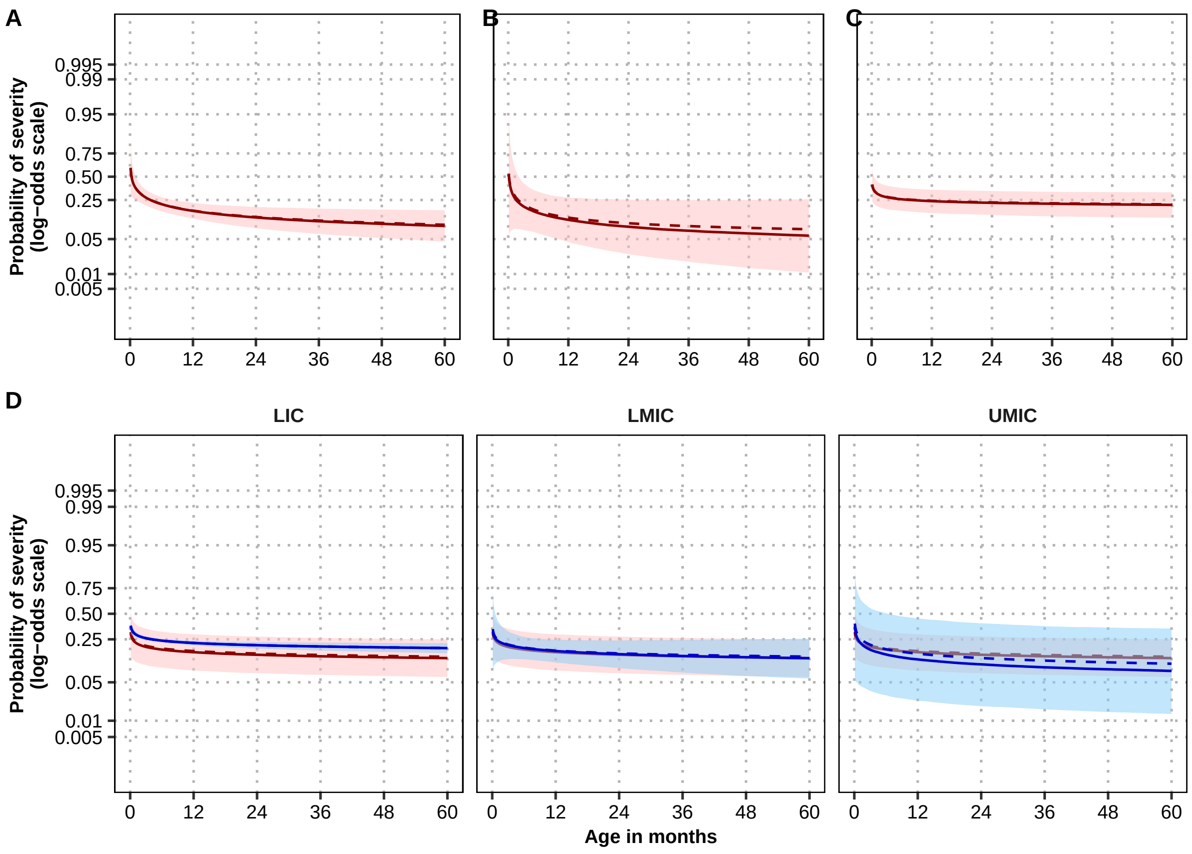

Figure B: Splines of severe and very severe disease among community-based and hospital-based cases. A) the probability of severe cases among cases in the community, B) the probability of very severe cases among cases in the community, C) the probability of severe cases among hospitalized cases, and D) the probability of very severe cases among hospitalized cases. Because the income group designation of the country where the study took place was not significantly associated with differential incidence, then only global estimates are displayed for the probability of severe and very severe cases among community cases (panels A and B, respectively), as well as the probability of severe cases among hospitalized cases (panel C). The bands correspond to the 95% confidence intervals of each parameter at each age.

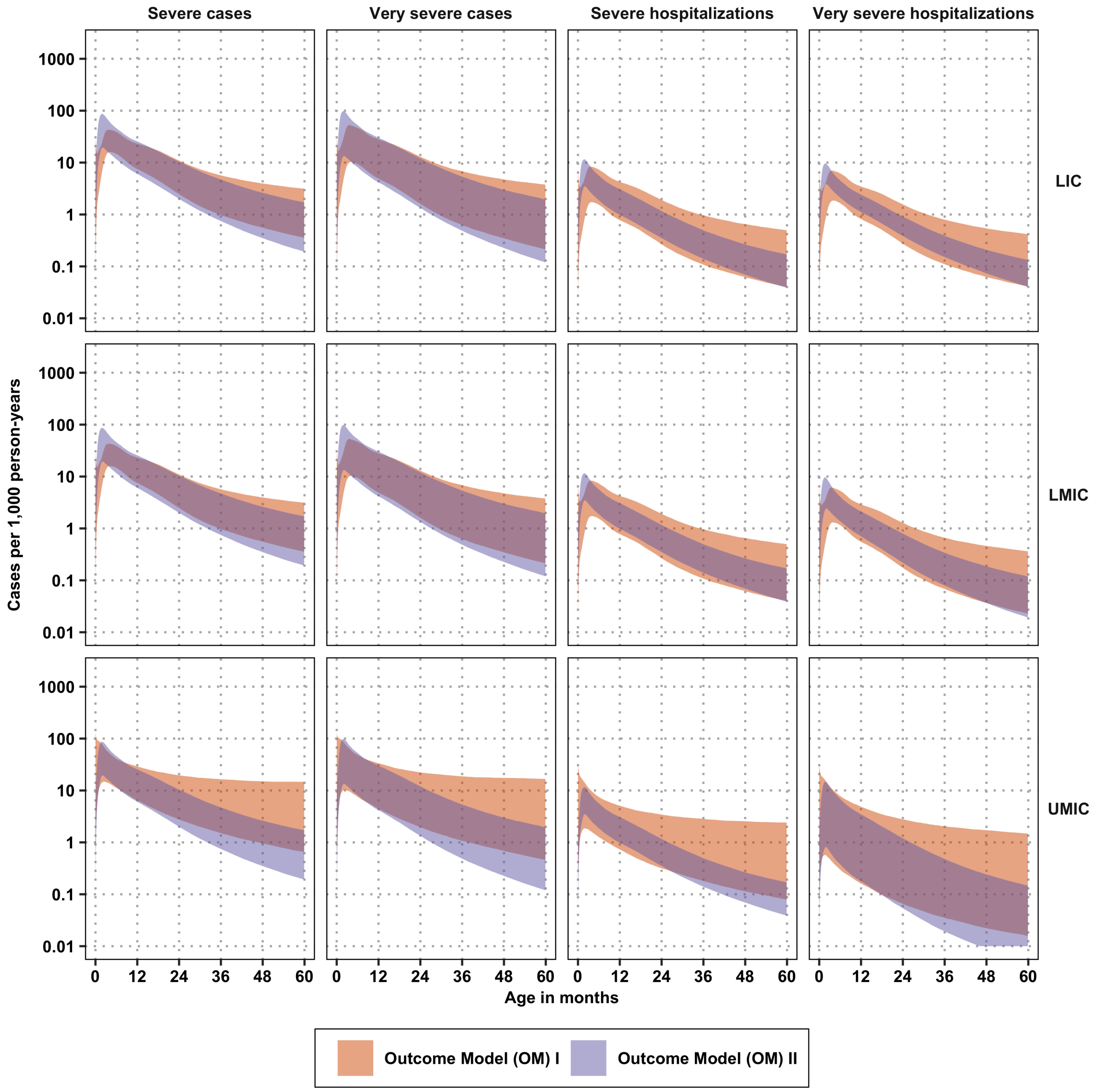

Figure C: RSV cases, hospitalizations of severe and very severe disease per 1,000 person-years according to Spline Models (SM) I and II. Number and 95% confidence intervals of hospitalizations, and deaths per 1,000 person-years according to Spline Models (SM) I and II, detailed in Fig 1 and S1 Text, Section S1.4). The green bands arise from OM I, calculated by taking the splines of community-based incidence (Spline I), probability of hospitalization (Spline III), and probability of death among hospitalized cases (Spline IV). The orange bands arise from OM II, calculated by taking the splines of hospital-based incidence (Spline II), probability of hospitalization (Spline III) to back-calculate cases in the community, and probability of death among hospitalized cases (Spline IV). Differences among income-group regions arise from differences in the underlying age distribution of cases between regions, as the trend of severity was the same across regions, except in OM II for very severe hospitalizations, as the trend for very severe cases was different between regions. Abbreviations: LIC: low-income countries, LMIC: lower-middle-income countries, UMIC: upper middle-income countries.

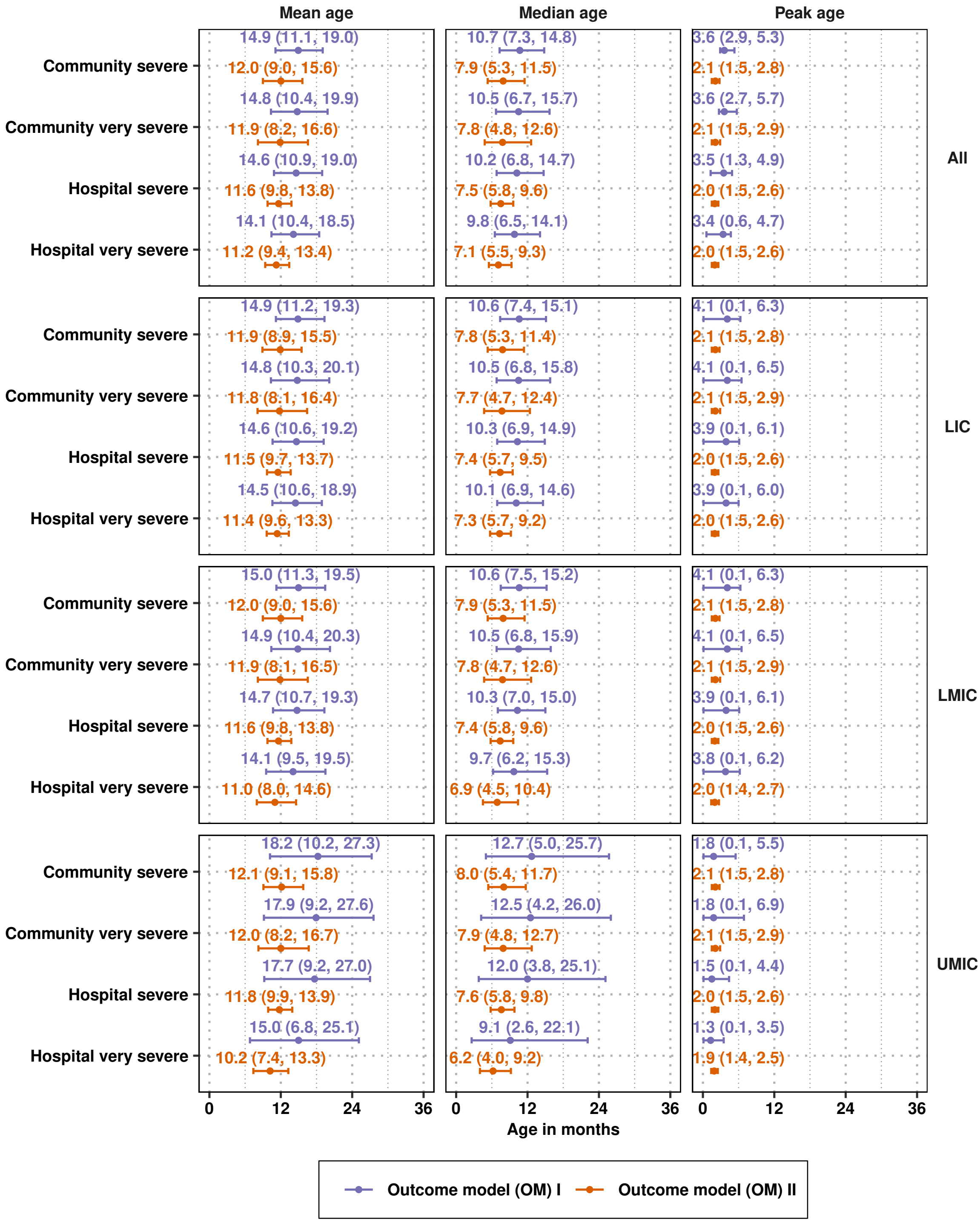

Figure D: Mean, median, and peak age of severe and very severe disease among community-based and hospital-based cases. The segments correspond to 95% confidence intervals of each of the summaries and the dots correspond to the median estimate of each of the summaries. Differences among income-group regions arise from differences in the underlying age distribution of cases and hospitalizations between regions. Abbreviations: LIC: low-income countries, LMIC: lower-middle-income countries, UMIC: upper middle-income countries.

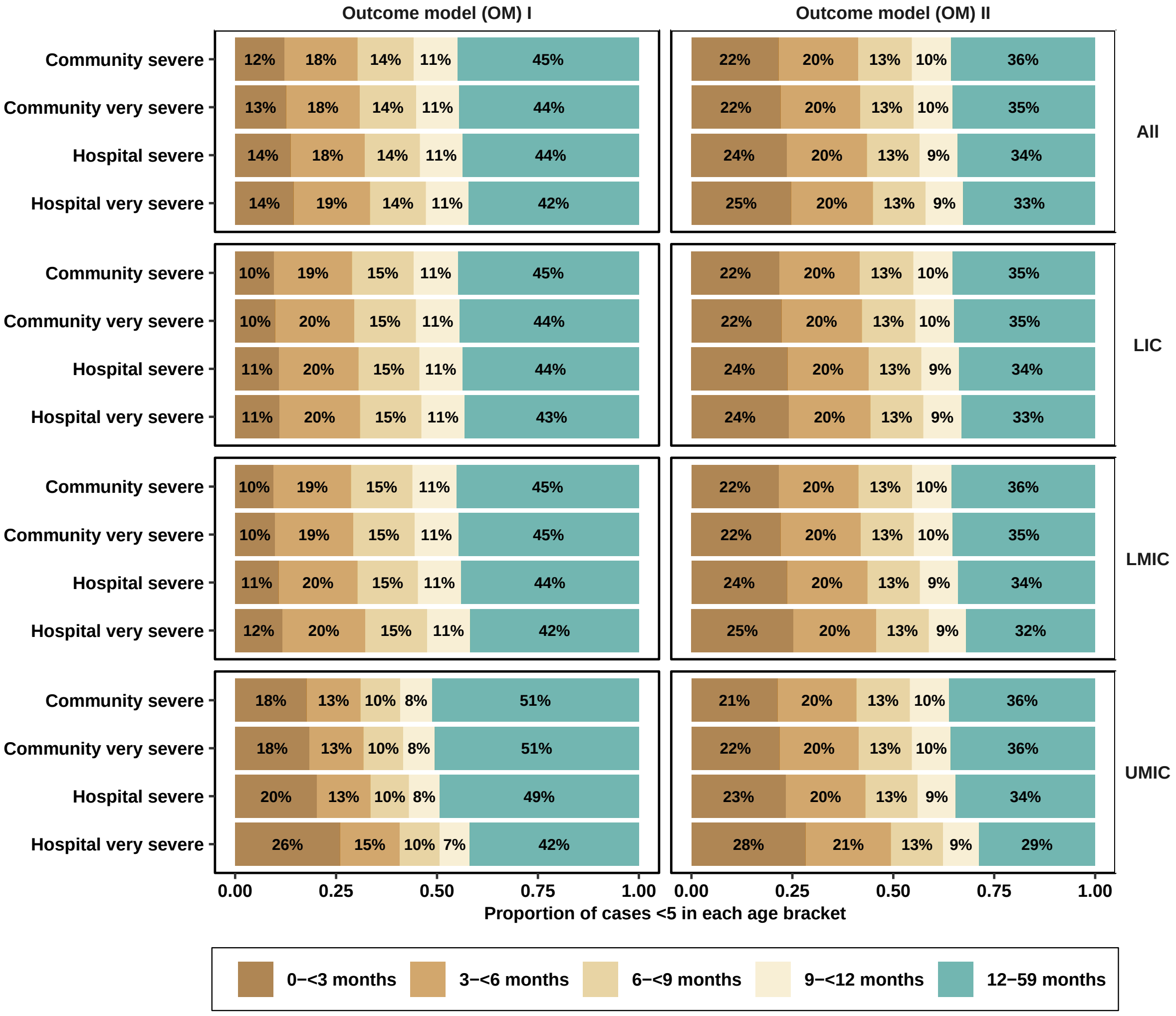

Figure E: Proportions of each outcome that fall under key age brackets for severe and very severe case burden. Differences among income-group regions in OM I arise from differences in the underlying age distribution of cases between regions. Abbreviations: LIC: low-income countries, LMIC: lower-middle-income countries, UMIC: upper middle-income countries.

S3.3 Additional results of burden models (BMs) by income group and by age

↩ Return to the Table of Contents.

Below are the results of the burden models by income group and age.

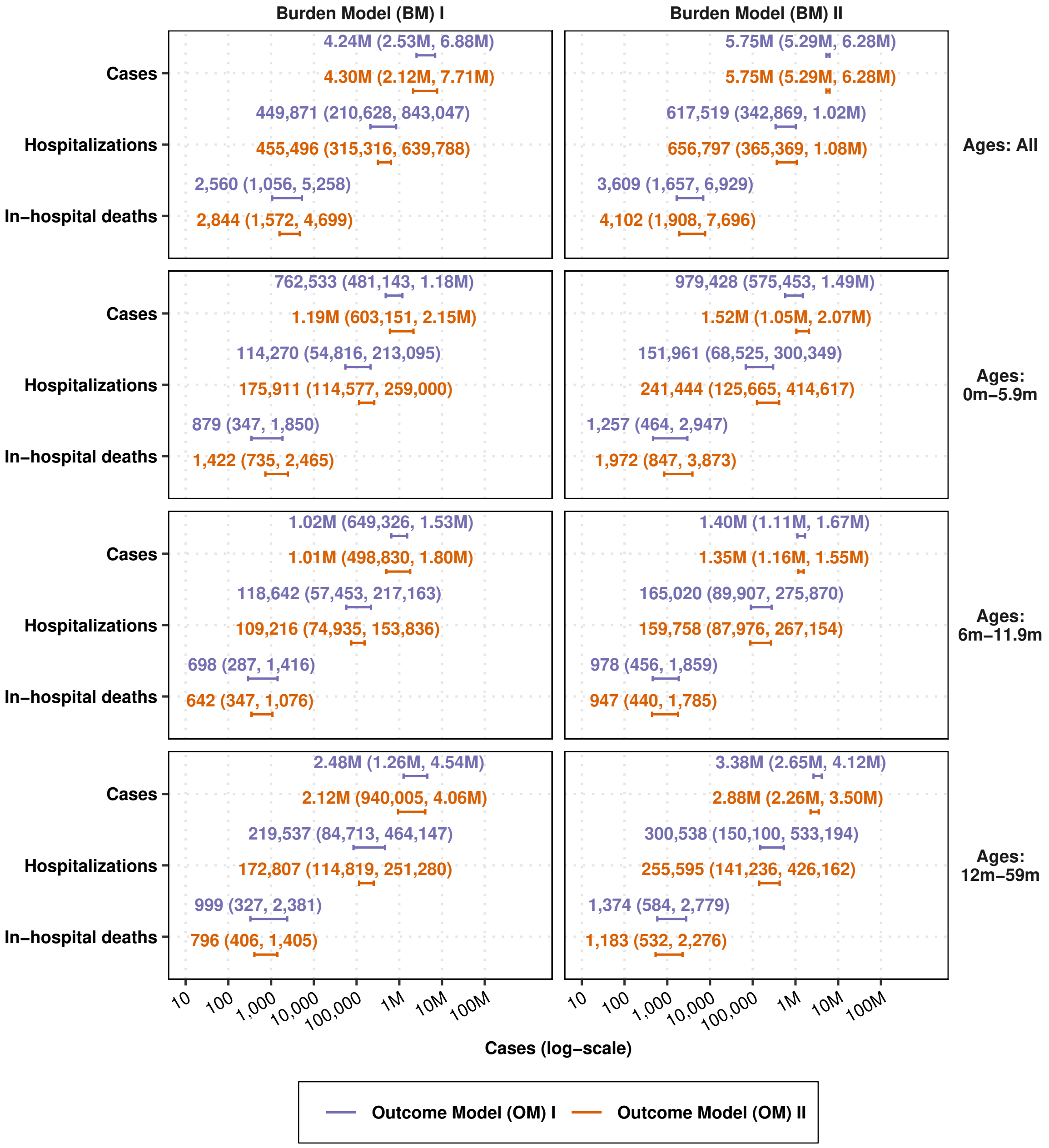

Figure F: Sensitivity analysis of the burden of RSV cases, hospitalizations, and deaths by age in low-income countries (LICs). Number and 95% confidence intervals of hospitalizations, and deaths per 1,000 person-years according to Spline Models (SM) I and II and Burden Models I and II, as detailed in Fig 1 and Sections S1.4 and S1.6 in S1 Text. Abbreviations: LIC: low-income countries, LMIC: lower-middle-income countries, UMIC: upper middle-income countries.

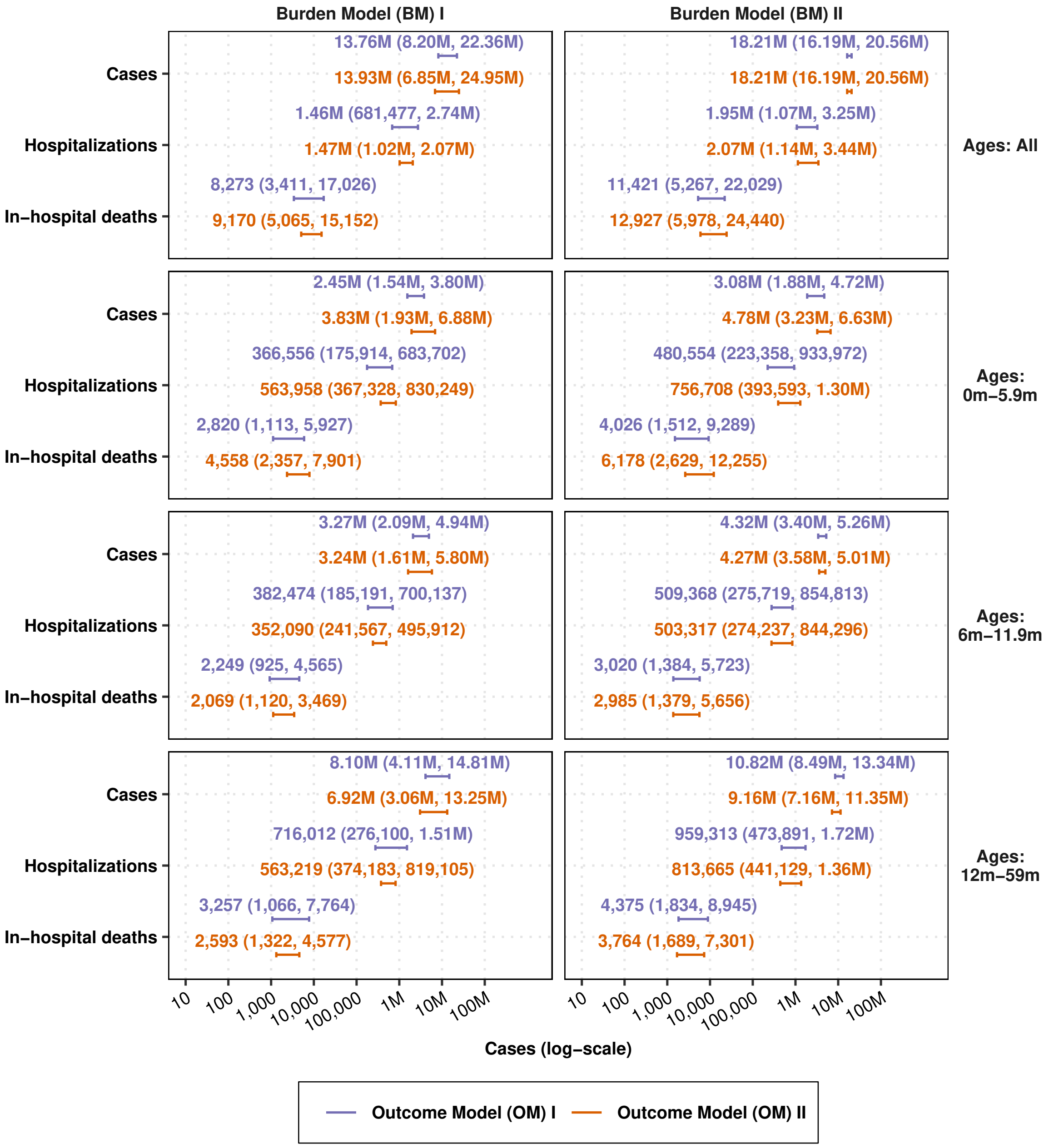

Figure G: Sensitivity analysis of the burden of RSV cases, hospitalizations, and deaths by age in lower-middle-income countries (LMICs). Number and 95% confidence intervals of hospitalizations, and deaths per 1,000 person-years according to Spline Models (SM) I and II and Burden Models I and II, as detailed in Fig 1 and Sections S1.4 and S1.6 in S1 Text. Abbreviations: LIC: low-income countries, LMIC: lower-middle-income countries, UMIC: upper-middle-income countries.

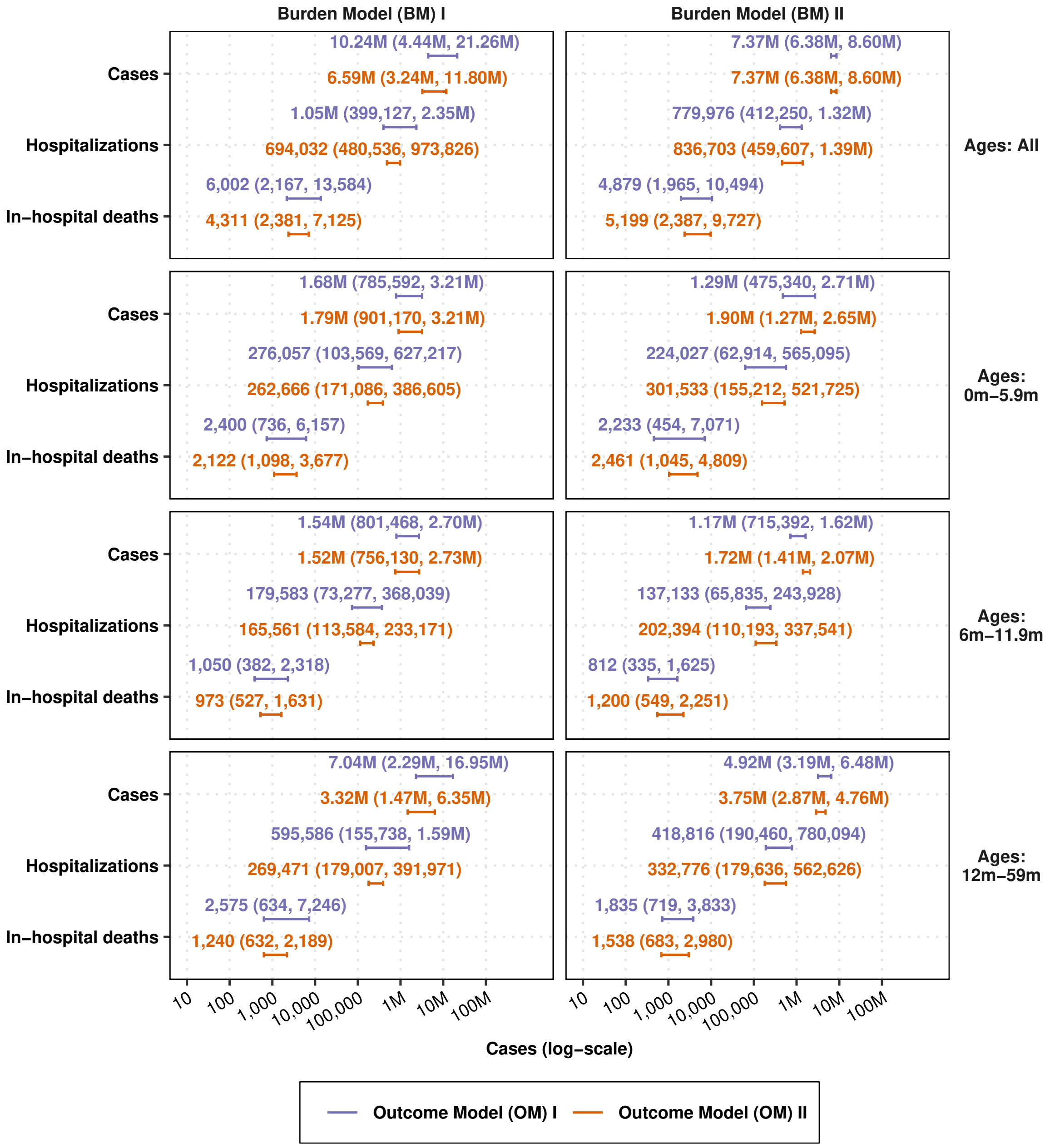

Figure H: Sensitivity analysis of the burden of RSV cases, hospitalizations, and deaths by age in upper-middle-income countries (UMICs). Number and 95% confidence intervals of hospitalizations, and deaths per 1,000 person-years according to Spline Models (SM) I and II and Burden Models I and II, as detailed in Fig 1 and Sections S1.4 and S1.6 in S1 Text. Abbreviations: LIC: low-income countries, LMIC: lower-middle-income countries, UMIC: upper-middle-income countries.

S3.4 Deaths in the community and total deaths

Burden Model	Outcome Model	LIC	LMIC	UMIC	All regions
**Community deaths**					
I	I	2,794 (744, 6,885)	9,029 (2,406, 22,252)	6,554 (1,574, 17,148)	18,376 (5,074, 43,473)
I	II	3,100 (1,008, 6,548)	9,993 (3,249, 21,145)	4,698 (1,528, 9,949)	17,790 (5,783, 37,661)
II	I	3,939 (1,161, 9,312)	12,458 (3,605, 29,455)	5,314 (1,403, 13,596)	21,711 (6,347, 51,097)
II	II	4,478 (1,319, 10,346)	14,105 (4,119, 32,967)	5,675 (1,677, 13,142)	24,257 (7,070, 55,993)
**In-hospital deaths**					
I	I	2,560 (1,056, 5,258)	8,273 (3,411, 17,026)	6,002 (2,167, 13,584)	16,835 (7,333, 33,429)
I	II	2,844 (1,572, 4,699)	9,170 (5,065, 15,152)	4,311 (2,381, 7,125)	16,326 (9,018, 26,972)
II	I	3,609 (1,657, 6,929)	11,421 (5,267, 22,029)	4,879 (1,965, 10,494)	19,909 (9,178, 38,076)
II	II	4,102 (1,908, 7,696)	12,927 (5,978, 24,440)	5,199 (2,387, 9,727)	22,229 (10,328, 41,763)
**All deaths**					
I	I	5,354 (1,966, 11,897)	17,303 (6,342, 38,405)	12,556 (4,095, 30,333)	35,212 (13,586, 74,023)
I	II	5,944 (2,857, 10,853)	19,163 (9,207, 34,976)	9,009 (4,326, 16,428)	34,116 (16,390, 62,260)
II	I	7,548 (3,093, 15,794)	23,879 (9,737, 49,971)	10,193 (3,635, 23,537)	41,620 (17,071, 86,434)
II	II	8,579 (3,521, 17,717)	27,032 (11,239, 55,338)	10,874 (4,459, 22,034)	46,485 (19,320, 94,423)

Table B: All deaths: deaths in the community (which did not reach a health facility), in-hospital deaths, and total deaths.
