# Supplementary material for: The age profile of respiratory syncytial virus burden in preschool children of low- and middle-income countries: A semi-parametric, meta-regression approach
Source: PLoS Med. 2023 Jul 17;20(7):e1004250. doi: 10.1371/journal.pmed.1004250 (PMC10389726; doi:10.1371/journal.pmed.1004250)
Supplement: Attachment — Submitted filename: Reviewer comments FINAL.docx [file pmed.1004250.s005.docx]

Responses to Reviewers

[Notes about procedures	1](#_Toc132033188)

[From the editors	1](#_Toc132033189)

[Reviewer #1 comments	3](#_Toc132033190)

[Reviewer #2 comments	5](#_Toc132033191)

[Reviewer #3 comments	7](#_Toc132033192)

## Notes about procedures

- Check resubmission guidelines:  <http://journals.plos.org/plosmedicine/s/revising-your-manuscript>
- upload any figures associated with your paper as individual TIF or EPS files with 300dpi resolution at resubmission; please read our figure guidelines for more information on our requirements: <http://journals.plos.org/plosmedicine/s/figures>
  - A note about using PACE: <https://pacev2.apexcovantage.com/>
- Change title to **The age profile of respiratory syncytial virus burden in pre-school children of low- and middle-income countries: A semi-parametric, meta-regression approach**
- Tell the editor about the title change
- The new figure and the move of the last table to the supplement.

## From the editors

| Comment | Response |
| --- | --- |
| Comments from the Academic Editor: | |
| Please rewrite the text a bit to make it less technical and add a bit more interpretation of the results. | Thanks. We condensed the methods and added more language in the discussion to connect our findings with the wider implications.  We also added figure 5 to make the results easier to interpret in policy discussions. |
| I found it a bit confusing that in the methods section there was a section entitled "Window of protection" and in the results section "Window of infection". Is that the same? Also, term window seemed a bit misleading, as it is about certain aspects of a distribution. I think the authors could do more to clarify what they mean by window of protection and how the results can be interpreted in that way. | Both have now been changed to “Age profile of RSV burden” since we are not analyzing the prophylactic itself. |
| One of the reviewers remarked on that the estimates were for the year 2020 and that the authors should comment on the impact of the pandemic. It was not clear for me that the estimates were meant to be for 2020, I thought that the authors implicitly assumed a sort of endemic (albeit seasonal) state. But I agree that some comments on the impact of the pandemic would be important. | We now refer to this as “an average year” and make clear that we are assuming an endemic equilibrium in the discussion, and that we have not analysed (and don’t have data from enough countries to analyse) a potentially lasting impact of the pandemic, especially on the seasonal pattern which we sidestep in this analysis. |
| Another reviewer suggested to not use two ways to calculate outcomes, but I found this actually good, because it gives additional validity to the estimates. | Thank you, we have retained this, but we clarified why we have done this in the methods: page 4 line 92. |
| Requests from the Editors: | |
| Please ensure that all numbers presented in the abstract are present and identical to numbers presented in the main manuscript text. | We have double checked this. |
| In the last sentence of the Abstract Methods and Findings section, please describe the main limitation(s) of the study's methodology. | That’s a great suggestion, we have added this sentence to the abstract:  *Our study is limited mainly due to data limitations, especially for low-income countries. The lack of information for some populations makes detecting heterogeneity between income groups difficult, and differences in access to care may impact the reported burden and limit its transferability.* |
| At this stage, we ask that you include a short, non-technical Author Summary of your research to make findings accessible to a wide audience that includes both scientists and non-scientists. The Author Summary should immediately follow the Abstract in your revised manuscript. This text is subject to editorial change and should be distinct from the scientific abstract. Please see our author guidelines for more information: <https://journals.plos.org/plosmedicine/s/revising-your-manuscript#loc-author-summary> | An author summary has been provided in page 2. |
| Please remove the Code Availability, Role of the funding source, and Author Contributions sections from the main text and ensure the relevant information is captured in the Submission Form questionnaire. | Thanks for pointing it out. This has now been done. |
| For internet-derived references, please include the date accessed. |  |
| S1 Supporting Text, Figure A: Please confirm that the appropriate usage rights apply to the use of this map. Please see our guidelines for map images: <https://journals.plos.org/plosmedicine/s/figures#loc-maps> | We have made sure the new map doesn’t have any usage rights issues. |

## Reviewer #1 comments

Antillon and colleagues reported a secondary analysis of the burden of RSV infections in infants of LMICs based on two global systematic reviews published in 2017 and 2022. The authors leveraged flexible generalized additive mixed models (GAMM) to estimate the age-dependent incidence and mortality of RSV infections, which would allow more flexibility for understanding the age distribution of RSV infections of different severity. The authors went ahead and estimated the global number of RSV infections and deaths in 2020, and compared those estimates with the two global systematic reviews. The authors showed that depending on the study settings, the peak, mean and median age of RSV infections could vary; these findings could help optimize the timing (of age) for RSV immunisation products given the anticipated short duration of protection.

Overall, this analysis has its merits for an improved understanding of the age distribution of RSV cases and potentially deaths. The methodology for estimating the age-specific incidence and probability appears to be sound in general. However, the second part that estimated the absolute number of infections and deaths was problematic (see the comments below), and reading through the manuscript, it looks more like a validation of the model rather than a formal estimate; it is not essential to the main message of the manuscript.

| # | Reviewer comment | Response |
| --- | --- | --- |
| Comments for Part 1 - age distribution | | |
|  | The authors assumed in their base model that the probability that a case in the community becomes a case in the hospital and that the hospitalized case becomes a fatality is itself age-dependent. This holds partly true - the probability is also expected to be dependent on country's income.   - Accessibility and availability of hospital care is likely to be different across income groups (e.g., UMICs better than the other two), so is quality of health-care that is associated with in-hospital CFR. Although the authors explored income group as a fix effect in secondary model (and tested the added value albeit not being able to rule out false negative), I would consider swapping the two models and making income-specific splines the primary model. This is important especially if the age distribution of RSV cases and deaths differed by income (as seen in RSV GOLD studies). - I appreciate that the authors might have limited data from certain income regions (e.g., LIC) but that should not be the sole reason for not going for income-specific model as the primary model. If necessary, LIC and LMIC can be combined. | To be clear, the income group was retained for the community-based incidence, but not for the hospital-based incidence nor the conditional probability of hospitalization nor death in the hospital. In fact, for the community-based incidence, we did combine LIC and LMIC, as there were no LIC community-based incidence studies. Moreover, it is interesting that the analysis could detect a difference between income groups for community-based incidence, which was based off of only 18 studies, but not the far-better powered hospital-based incidence or hospital-based CFR, for which we had 37 and 58 studies respectively from LICs, LMICs, and UMICs.  The potential inclusion of country-level World Bank income group is a valid factor to explore, but there doesn’t seem to be any statistical evidence to suggest that such a model is more appropriate, even if a different dataset found this association.  By RSV GOLD studies, we are assuming you mean Lowensteyn 2023 and Mazur 2021 specifically, which showed the median age of death was 1.5 months in LMICs and 5 months in UMICs and that this difference was statistically significant. The RSV GOLD registry is an incredible source of information, but because it is a “convenience” sample with voluntary participation, we do not want to constrain our overall incidence according to the RSV GOLD data. However, we have cited those studies in the discussion. |
|  | Deaths in the community - I would leave out the deaths in the community (or move to the appendix at least) for two reasons. First, as the authors pointed out, data are sparse (compared to incidence and hospitalisation data) and are not suitable for GAMM models. Second, the alternative approach (that applied an inflation factor, Shi et al.) was based on an additional assumption about the proportion of RSV episodes that could access hospital care. The resulted estimates would not be as robust as the estimates of incidence and hospitalisations and undermined the overall quality of the work. | We have decided to move it to the appendix. Although this was not the most exciting result, it is provided for completeness for the burden secondary analysis and death is a main outcome of interest. |
|  | I am confused by the decision to estimate the absolute number of global and regional RSV infections in the year of 2020 - the COVID-19 pandemic year when RSV epidemiology changed substantially (e.g., incidence, severity and age distribution). I do not see any additional efforts for accounting for the impact of the pandemic nor any justifications.   - Reading through the manuscript, this part looks more like an informal validation of the GAMM models used above - then I would use the years of 2015 and 2019, respectively for validating the estimates by Shi et al and Li et al. This part could potentially go to the appendix. | That is an excellent point. We assumed an endemic level of RSV with a population structure equivalent to estimates of the population in countries in 2020. You are right that 2020 was a very unusual year and we should have remarked on the fact that our results do not apply for that specific year but for a “normal” year with the population sizes seen in 2020.  Wherever we talk about the cases in 2020 we are now mentioning an “average” year.  Predicting how RSV may have changed with the pandemic is beyond the scope of this paper, and one cannot yet know if those changes are permanent or will just last a handful of seasons. Still, we missed adding some perspective on this in the discussion/limitations section and do so now:  *Fourth, the RSV seasonality has been impacted by the Covid-19 pandemic, and continuous surveillance is essential to monitor RSV seasonal patterns across global regions \cite{Mosscrop2022, Mohebi2022, Billard2022}.* |
| Comments for "window of protection" | | |
|  | I believe this is the meat of the paper and deserves more attention and work. Can the authors consider estimating the proportion of the overall burden in <1y or <5y for different RSV prophylactic products and for different schedules (e.g., birth dose or later in life)? This provides more straightforward practical information for decision-makers in addition to peak, mean and median age. | Yes, because this is indeed quite a useful number and it resonates with another comment from another reviewer, we added Figure 5, which describes the % of cases, hospitalizations, and deaths by 3-month age groups in the first year of life to provide an easy estimate of how different products with different durations may help. We also added language to the 3^rd^ paragraph of the discussion to connect what our results imply about current strategies and products:.  *Moreover, our findings imply that prophylactics that offer protection until 4-6 months of age will target the peak age of deaths but are unlikely to avert even 50\% of deaths (indicated by the median in Figure 4 and Figure 5). The longest lasting form of passive immunity -- in the form of a monoclonal antibody, Nivursimab -- so far protects for 150 days \cite{Hammitt2022}. 20\%-29\% of cases occur in children under 6 months of age, but for deaths, the proportions are much higher: 38\%-50\%. As protection from such a product wanes in the fifth and sixth month, our findings indicate that a second product would be helpful as well. This is consistent with cost-effectiveness analyses, that show that the use of a single prophylactic must be priced competitively in order to prove cost-effective in most low- and middle-income settings \cite{Li2020, Laufer2021}. While the data on severity was less clear and was relegated to supplemental analysis, severe RSV disease showed a higher concentration in younger infants as well (S3 Text, Figure~\ref{fig:window_supplement_sev}-\ref{fig:percent_covered_sev}).* |
| Other comments | | |
|  | Writing style - while I appreciate that the authors described their methodology in great details, the overall text looks a bit too technical, especially considering the readerships of PLoS Med. This is for both the method and discussion sections. I spent almost half a day each to navigate through the amount of technical details.   - In the discussion section, I saw large amount of texts that compared numbers but few interpretation and appraisal of the estimates. | The reviewer’s suggestion is well-received and we have revised the text in the results and in the discussion to be more straightforward. Because this concerns changes throughout, we refer the reviewer to the tracked changes version of the new manuscript.  The Estimation Approach section of the Methods was removed as many details were redundant to other sections and some of the language was condensed and placed elsewhere. |

## Reviewer #2 comments

This study addresses an important research question about the age profile of RSV disease burden in developing countries. It has relevance for understanding the potential impact of various RSV vaccination strategies. Overall, the study is well-done and clearly written. The manuscript is lengthy and presents multiple results. However, this manuscript can be better summarized and emphasize on the most important findings. Specific comments are as follows:

| # | Reviewer comment | Response |
| --- | --- | --- |
| Major comments: | | |
|  | Lack of a clear description of the methods for different outcome models. Figure 1 panel A is very condensed, but the figure legend is more informative enough. The method description on page 6 is still not clear why the authors chose two different outcome models. I recommend the authors to phrase this part, including the following two burden models, as a base model and sensitivity analyses. | The information from the literature allows to calculate total cases in two different ways, and these two ways may arrive at different answers. That’s why we have two models. Moreover, the model using hospital-based incidence biases the average age of infection down.  We added this sentence (line 92, page 4) in order to be more forthcoming about our motivations:  *Moreover, we compare two outcomes models (OM): OM I, in which the starting point is community-based incidence, and then the conditional probability of hospitalization given infection and in-hospital death are applied, and the second, OM II, in which the starting point is hospital-based incidence, and the conditional probability of hospitalization is used to back-calculate community incidence and the probability of death is applied to calculate fatalities. In the absence of any reason to choose one model over another, we look at the results of both side-by-side.*  We also added this to the discussion (page 18, line 437)  *Because there is no way to weigh these models in a statistically principled manner (unless one arbitrarily assigns equal weights) we did not combine the models.*  Similarly, we have no way of deciding which of the two models is better, so we have avoided labeling one a base and the other a sensitivity model. |
|  | The section window of protection and the burden stratified by age in results should be combined and emphasized. This is the most significant and innovative finding this paper compared with others. Instead of presenting point estimates like mean, median, and peak age of infection, the authors should consider describing the distribution of the age profile and the impact of different vaccination strategies. Many previous papers already concluded the mean and median age of infection. In my opinion, what the author wrote in page 14 to 15, "…about a quarter to a half of all hospitalizations happen in the first 6 months of life…", is the most important finding for this manuscript. The author should consider expanding this part and discussing what percentage of children at risk can be protected with different type of vaccination strategy. | We left the age-specific burden section where it was, but we took the reviewer’s advice to describe the distribution of cases, hospitalizations, and deaths more clearly by adding figure 5, which breaks down the proportion of the burden by 3-month age groups in the first year of life to provide an easy estimate of how different products with different durations may help. We also added language to the 3^rd^ paragraph of the discussion to connect what our results imply about current strategies and products. |
| Minor comments: | | |
|  | The current title does not reflect the content of the manuscript. I would suggest changing the title to "The detailed age distribution of RSV burden in infants of low- and middle-income countries: a generalized additive mixed effect model approach" | We changed it to  *The age profile of respiratory syncytial virus burden in pre-school children of low- and middle-income countries: A semi-parametric, meta-regression approach*  (We used “profile” rather than “distribution” to avoid confusion with probability distributions) |
|  | The background of the abstract is disjoint from the following abstract. I would suggest adding a sentence to explain why estimate the age profile of RSV burden will help optimize the upcoming vaccination strategies. | The following sentence has been added:  *A notable challenge with many of the upcoming prophylactic interventions against RSV is their short duration of protection, making the age profile of key interest to the design of prevention strategies.* |
|  | The probability of hospitalization is a conditional probability. The author should make it clear in the first place. It should be "the probability of hospitalization given infection". | This is a good point, we cleared it up at the beginning of the methods section:   - age-specific conditional probability of hospitalization given (measured in community-based studies) - age-specific conditional probability of death given hospitalization (hCFR) (measured in hospital-based studies) |
|  | Page 2 line 17, statement: "maternal vaccines protect children no more than four months". Pfizer announced the results of a phase 3 clinical trial on maternal RSV vaccine and the protection is beyond 150 days. | This has now been cited and mentioned in the introduction. |
|  | Page 2 line 25: The word "temporal resolution" is confusing. This paper does not measure RSV burden over calendar time as a time-series analysis but provide the detailed age profile of RSV burden. | The sentence has been changed to:  In light of the importance of the duration of protection and the presence of a potential high cost, it is imperative to develop detailed age-specific estimates of incidence, hospitalization, and mortality that can be incorporated with careful economic analyses that weigh the costs and the benefits of options for using these products. |
|  | Page 4 line 81: The author should clarify that the "month-specific epidemiology" is age in month in pediatric populations. | The sentence is now:  Since the data on RSV epidemiology in infants and pre-school children is usually presented in age groups spanning several months or up to a year, making the month-by-month age-specific epidemiology a partially observed process, we sought a method to estimate monthly interpolations of incidence, hospitalization, and mortality before the age of 5. |
|  | Page 8: "The scenario analysis: severity of RSV' seems to be disjoint from the main manuscript. The author should consider moving this section into the supplementary document and acknowledging the lack of severity measures as a limitation. | The issues with severity are now listed in the limitations, although we have left the mention of them in the main paper because it would be of interest to many medical practitioners. |
|  | Page 9 line 245 is unclear. What is the meaning of "the trend overall is higher"? | The incidence at most ages. To be accurate we now write:  *and the overall incidence is higher in UMICs than in LMICs* |
|  | Page 9 line 254. Large "amount". | We have removed those two words.  *The peak incidence at UMICs was difficult to ascertain because of the heterogeneity in age profiles across countries.* |
|  | Page 9 line 259. What is the peak incidence? Hospitalization incidence? Community-based incidence? | Hospital-based incidence. We have added the clarification. |
|  | Page 9 line 263. For "12,77%", the decimal symbol should use the same format across the paper. | Those decimals have been removed as they were not substantially informative. |

## Reviewer #3 comments

Thanks for the opportunity to review your manuscript. My role is as a statistical reviewer, so my review concentrates on the study design, data, and analysis that are presented. I have put general questions first, followed by queries relevant to a specific section of the manuscript (with a page/line reference).

This manuscript estimates incidence of RSV (community, hospitalisations, and deaths from) across early childhood across a selection of lower and middle-income countries. Suitable data (with sufficient numbers of age-groups and from 200 onwards) were taken from a previous systematic review. An interesting approaching using generalised additive mixed models was applied - this enabled a country specific estimate of age-specific incidence of RSV with a random parameter for the spline effect of age. Sub-group effects were considered in the incidence and probability parts of the model, e.g. heterogeneity by country income group. Aggregated age-group data was dealt with my considering the total incidence/probability for an age-group to be representative of the mid-point of the interval. That is probably a reasonable approach for population level data. Shrinkage was employed to find an optimal number of knots for each of the splines. Two approaches to estimating burden were considered - these give very similar point estimates with OM generally having wider CI than OM I. This is a fairly complex approach - the detailed appendices (and github repository) were a great help for this review. The explanation and figures are well presented in terms of explaining the approach. The GAMMs used are a good way to estimate the target epidemiological model (e.g. incidence in community->hosp->death), but whether this model of burden is reasonable representation of the process, or if the underlying data from the systematic review is of sufficient quality to be used in this way is something I don't have the expertise to judge myself.

I have worked with population level RSV data (a long time ago) and at the time I was surprised by the discrepancy between the burdens it represented in my own country compared to the relative level of attention paid towards this disease. I think that the overall aims and research questions proposed here are good (albeit this judgement comes from someone without substantial subject-matter expertise on RSV).

| **#** | **Reviewer comment** | **Response** |
| --- | --- | --- |
|  | One query I had was around generalisability of the findings from the included countries to all low and middle-income countries, e.g. good amounts of coverage for Central America, but limited data available for North Africa and Central Asia. The results should be internally valid to the countries included in the data, but I wonder if some of the language that generalises the results to all LMICs might not be appropriate? | We have added more prominent language in the abstract and in the limitations of the discussion to highlight the impact of limited data in many countries – in particular with regards to the lack of data in those countries termed “low-income” countries.  *Our study is limited mainly due to data limitations, especially for low-income countries. The lack of information for some populations makes detecting heterogeneity between income groups difficult, and differences in access to care may impact the reported burden and limit its transferability.* |
|  | Abstract, Methods and findings. I would reword the description of the secondary analysis so that it's clear that total RSV burden was estimated, at the moment the difference between the primary and secondary analyses is hard to distinguish because the phrases 'across different settings 'and 'across settings' look similar but actually mean completely different things. | While we are not sure what the reviewer meant, we have removed the first instance of “across different settings” and changed the second to low-and middle income countries to be specific.. |
|  | S.1.4 For the incidence part of the model, was over dispersion checked? Were residuals also examined for excess 0's? Given population is an offset I would guess this would unlikely to be an issue | We have checked overdispersion and added a note about this in page S1.9 of the supplement. |
|  | S1.4.1 "…we excluded studies that fewer than three studies…" - should the last 'studies' here be 'age-groups'? | You are completely right. This has been rectified. |
|  | S.1.4.2 What is the advantage of 5,000 draws from the MVN distribution compared to taking predictions directly from 5 and 95th percentile (or whatever limits are appropriate)? | We used samples from the MVN distribution of each outcome (incidence, pr. hospitalization given infection, and CFR given hospitalization) to multiply the outcomes from the three models to get the total number of hospitalizations and in-hospital deaths. We wouldn’t want to multiply the 2.5^th^ and 97.5^th^ percentiles of the incidence and probability models to get the numbers of outcomes, as this would have given us far wider distributions that would not be statistically sound. |
