# Supplementary material for: The age profile of respiratory syncytial virus burden in preschool children of low- and middle-income countries: A semi-parametric, meta-regression approach
Source: PLoS Med. 2023 Jul 17;20(7):e1004250. doi: 10.1371/journal.pmed.1004250 (PMC10389726; doi:10.1371/journal.pmed.1004250)
Supplement: Attachment — Submitted filename: Reviewer comments.docx [file pmed.1004250.s006.docx]

**Requests from Editors:**

GENERAL
Thank you for detailed and considered responses to previous editor and reviewer comments.  Please see below for further comments which we require that you address prior to publication.
Please start your numbering at line 1 of the abstract (as opposed to the introduction).

**The numbering now begins at the abstract.**

ABSTRACT
Final paragraph of methods and findings and the beginning of the conclusions, please temper the language used when reporting your results, the phrase, ‘we estimate...’ or similar may be helpful.

**These changes have been made.**

AUTHOR SUMMARY
Thank you for including an author summary. The author summary should consist of 2-3 succinct bullet points under each of the following headings:
• Why Was This Study Done? Authors should reflect on what was known about the topic before the research was published and why the research was needed.
• What Did the Researchers Do and Find? Authors should briefly describe the study design that was used and the study’s major findings. Do include the headline numbers from the study, such as the sample size and key findings.    
• What Do These Findings Mean? Authors should reflect on the new knowledge generated by the research and the implications for practice, research, policy, or public health. Authors should also consider how the interpretation of the study’s findings may be affected by the study limitations. In the final bullet point of ‘What Do These Findings Mean?’, please describe the main limitations of the study in non-technical language.
We encourage you to review published articles on our website for examples. Please also see our author guidelines for more information: <https://journals.plos.org/plosmedicine/s/revising-your-manuscript#loc-author-summary>

**The author summary has been restructured to fit the new format.**

STATISTICAL REPORTING
Page 9 onwards - when reporting 95% CIs, we suggest the use of commas to separate upper and lower bounds instead of hyphens as these can be confused with the reporting of negative values. Please check and amend throughout including all sections of the main manuscript text, figures (and tables) and supplementary files where relevant.

**These changes have been made in the text, the figures, and the supplement.**

FIGURES
Please consider using/confirm usage of a colour palette suitable to those with colour blindness (i.e. avoiding red and/or green) to improve accessibility of your figures.

**The color palettes have been changed to remove green. Pink or red has been kept in figures where no green was also found so that the two colors would not be confused. We checked them against a colorblindness website. It was not possible to avoid red or green entirely as there were a lot of figures and a couple of different color palettes to indicate different kinds of results, but we made sure that the figures were at least still informative even for colorblind readers.**

DISCUSSION
Please revise the structure of your discussion which should begin with a short, clear summary of the article's findings; what the study adds to existing research and where and why the results may differ from previous research; strengths and limitations of the study; implications and next steps for research, clinical practice, and/or public policy; one-paragraph conclusion. Please do not include any sub-headings in the text such that your discussion reads as a single piece of continuous prose.

**The structure has been revised. This required us to enter three short new paragraphs to summarize the results.**

REFERENCES
For in-text reference callouts please remove spaces between citations, for example, line 24 (introduction) ‘…[20, 28, 29]…’ should read ‘…[20,28,29]…’ Please check and amend throughout all sections of the manuscript.

**The references have been revised.**

SUPPORTING INFORMATION
S1.4.1 and S1.8 – contains red text which may not be accessible to those with colour blindness. Please consider revising the use of red (and/or green).
S1 page 19 – please ensure that referencing format follows that of PLOS Medicine’s guidance which can be found here <https://journals.plos.org/plosmedicine/s/submission-guidelines#loc-references>
Please ensure that journal name abbreviations are those found in the National Center for Biotechnology Information (NCBI) databases. 
Please ensure that up to but no more than 6 author names are listed followed by et al, if more than 6 authors contribute to an individual study.
Please ensure that all web references include an access date.

**These changes have been made. Thanks for pointing these out. It was not possible to avoid red or green entirely as there were a lot of functions, but we made sure that the figures were at least still informative even for colorblind readers.**

SOCIAL MEDIA
If not already done so, to help us extend the reach of your research, please detail any Twitter handles you wish to be included when we tweet this paper (including your own, your co-authors’, your institution, funder, or lab) in the manuscript submission form when you re-submit the manuscript.

**We will enter these handles: @RESCEUproject, @LanderWillem, @BeutelsPhilippe, @markjit, @swisstph**
